# Supplementary material for: Coproduced, arts interventions for nurturing care (0–5 years) in low-income and middle-income countries (LMICs): a realist review
Source: BMJ Open. 2024 May 17;14(5):e083093. doi: 10.1136/bmjopen-2023-083093 (PMC11103195; doi:10.1136/bmjopen-2023-083093)
Supplement: Supplementary data [file bmjopen-2023-083093supp001.pdf]

"Co-produced, arts interventions for nurturing care (0-5 years) in Low- and Middle-Income Countries (LMICs): a realist review."

Gale et. al (2024) BMJ OPEN

Supplementary materials

Supplementary Table 1: Search Strategy and Terms

Database	Outcomes	Arts	Community	LMIC
ProQuest – ASSIA and Education databases	In all fields:<br><br>(‘Child health’ OR ‘infant health’ OR ‘baby health’ OR ‘early years health’ OR ‘nurturing care’ OR ‘child care’ OR ‘child feeding’ OR ‘breastfeeding’ OR ‘hygiene’ OR ‘diarrhoea’ OR ‘sanitation’ OR ‘maternal health’ OR ‘maternal care’ OR ‘paternal health’ OR ‘paternal care’ OR ‘child development’ OR ‘child education’ OR ‘child wellbeing’ OR ‘maternal wellbeing’ OR ‘maternal mental health’ OR ‘child mental health’ OR ‘social cohesion’ OR ‘community cohesion’ OR ‘trust’ OR ‘community support’ OR ‘community development’ OR ‘social norms’ OR ‘health practices’ OR ‘care practices’) AND	In abstract:<br><br>(‘Arts’ OR ‘drama’ OR ‘theatre’ OR ‘theater’ or ‘performative’ OR ‘creative’ OR ‘ music’ OR ‘story’ AND NOT 'antiretroviral') AND	In abstract:<br><br>(‘Community engagement’ OR ‘community mobilisation’ OR ‘community mobilization’ OR ‘community intervention’ OR ‘community-led’ OR ‘community campaign’ OR ‘group mobilisation’ OR ‘group mobilization’ or ‘participatory’ or ‘co-production’ OR ‘coproduction’ OR ‘coproduce’ OR co-produce’ OR ‘mobilisation’ OR 'mobilization') AND	In all fields:<br><br>(‘Low-income country’ OR ‘middle-income country’ OR ‘low income country’ OR ‘middle income country’ OR ‘LMIC’ OR ‘developing country’ OR ‘global south’ OR Afghanistan OR ‘Albania’ OR ‘Algeria’ OR ‘Angola’ OR ‘Antigua’<br>….<br>OR ‘Yemen’ OR ‘Zambia’ OR ‘Zimbabwe’<br>)

Gale NK, *et al. BMJ Open* 2024; 14:e083093. doi: 10.1136/bmjopen-2023-083093

SCOPUS	As above	As above	As above	As above but with countries selected in the filter by region or country option.
OVID: HMIC and Medline	As above	As above	As above	As above, but used OVID filter for country.
Web of Science	As above	As above	As above	As above, but Web of Science restricts the amount of keywords you can add to search query so filtered by country or region.
Child Education & Adolescence Studies	As above	As above	As above	As above, but for country used EBSCO filter.

Gale NK, *et al. BMJ Open* 2024; 14:e083093. doi: 10.1136/bmjopen-2023-083093

Supplementary Table 2: Relevant Criteria for Inclusion

Include	Exclude
Study type:<br>- Performs and reports an intervention, whether in an randomized control trial (RCT) design or in other forms (Quantitative or qualitative)<br>- Theoretical or critical articulation of community-based arts-based interventions	Not opinions, letters or editorials
Intervention is co-produced (can be led by community or public health, or equally balanced)	Conducted over 20 years ago
Focuses on early years water/food sanitation (0-5) or child development	Any language other than English, French, Spanish
Intervention takes place in LMIC	***Added after discussion:***<br>Health or nurturing care not a primary outcome, but as an unintended (if positive) outcome
Intervention is arts-based	Maternal health only as outcome
Added after discussion:<br>Intervention is arts-based, toward public health and normative behaviour change	'Community health' does not contain measure(s) using specific to under 5s
	Theory only (though useful for background section)
	Arts/story-based methodological evaluation only
	Arts-based intervention is individual therapy (no participatory aspect)

Gale NK, *et al. BMJ Open* 2024; 14:e083093. doi: 10.1136/bmjopen-2023-083093

Supplementary Table 3: Prompt questions used for appraisal of the interventions during data synthesis

appraise **co-production** by:	appraise **contextual adaptation** by:	appraise **arts-based implementation** by:
Whose input was sought/heard at which stage?	How was the context attended to by different phases or forms of contextual learning (e.g. observations, focus groups)?	What type of dramatic arts were utilised and who delivered/facilitated their use?
Was there balanced and reciprocal participation of stakeholders, researchers, local agents and community members in design/ implementation decisions?	Was there feedback (formal or informal) between design and implementation phases?	Was the intervention flexible? in the room? Was it adaptable logistically? i.e. time and place of delivery (to improve attendance)
Were any tensions or differences in goals noted between partners? If so, were causes and/or resolutions explored?	Was there explicit incorporation of local knowledge, local infrastructure or networks, local history and social/ economic/ geographic/ political differences or inequalities?<br>Note: beyond assuming this would happen automatically through participation of local people	Were any community groups excluded from participation? Were any groups notably less engaged, untrusting or sceptical upon delivery? Did any drop-out?
Intermediary-outcome hypotheses/ mid-range theories:<br>Is there evidence these brought about partnership synergy, partnership trust, targeting of equitably representative health/care/development issues, in a manner that are broadly supported and seen as effective. And, if so, to what extent?	*Intermediary-outcome hypotheses/ mid-range theories:*<br>Is there evidence these brought about effective, targeted health/care/ development information, and sustainable transmission of such information, equitable targeting of health/care issues. And, if so, to what extent?	*Intermediary-outcome hypotheses/ mid-range theories:*<br>Is there evidence these brought about explicit or experiential knowledge of health information, shared/communal experience of health information delivery, engaging with or challenging social norms, community alignment/ social binding or synergy, affective impact of health information delivery (e.g. motivation, shame, or empowerment). And, if so, to what extent?

Gale NK, *et al. BMJ Open* 2024; 14:e083093. doi: 10.1136/bmjopen-2023-083093

Supplementary Table 4: Search results breakdown

DB search engine	Database(s)	Results
EBSCO	Anthropology Plus, ASSIA, CINAHL Plus, EconLit, Education Abstracts (H.W. Wilson), Ergonomics Abstracts, ERIC, Humanities Abstracts (H.W. Wilson), Humanities Full Text (H.W. Wilson)	516
OVID	APA PsycArticles, Embase, HMIC, CAB Abstracts and Global Health, Ovid MEDLINE(R) and In-Process, APA PsycInfo, Social Policy and Practice, BooksOvid, journalsOVID	211
Proquest	Arts & Humanities Database, Biological Science Database,East & South Asia Database, East Europe, Central Europe Database, Education Collection, Environmental Science Database, Health & Medical Collection, India Database, International Bibliography of the Social Sciences (IBSS), Middle East & Africa Database, Nursing & Allied Health Database, Political Science Database, Psychology Database, Public Health Database, Religion Database, Social Science Database, Sociology Collection	568
Scopus		266
Web of Science		42
Child Adolescence and Education Studies		38
	Total	1641
	Duplicates removed	940
	Titles/abstracts screening	175

Supplementary Figure 1: Search strategy process overview

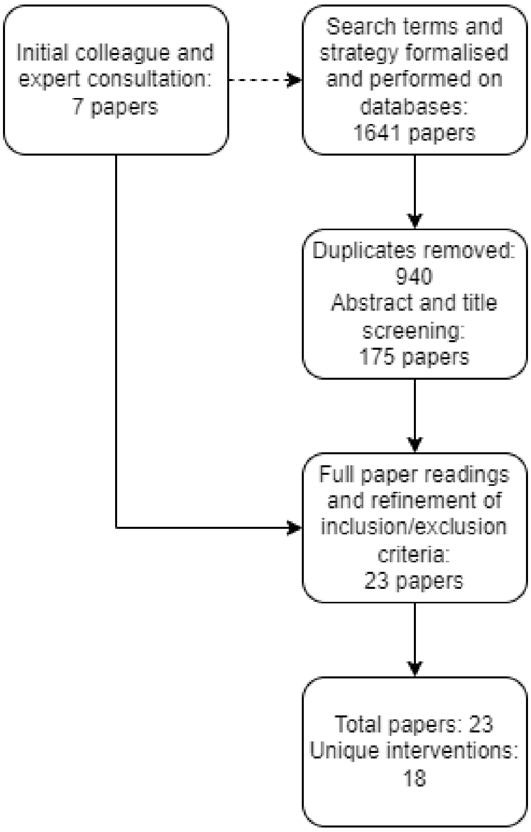

Supplementary Table 5: Summary of included papers

Author (Date)	Inter-vention Date	Title (s)	Country of intervention	Country of first author	Primary Discipline	Evaluation methods used	Intended Nurturing Care/Health Outcomes	Main Findings
Akter et al. (2020)	2017	Adaptation and Integration of Psychosocial Stimulation, Maternal Mental Health and Nutritional Interventions for Pregnant and Lactating Women in Rural Bangladesh	Bangladesh	Bangladesh	Public Health (Infectious Disease)	Qualitative evaluation (interviews) of a multi-phase adaptation process for an intervention.	Early childhood development	Most participants reported willingness to attend the sessions if extended for 1 year, and recommended additional visual cues and interactive role-play activities to make the sessions more engaging. Participants and community health workers found it difficult to understand the concept of “unhealthy thoughts” in the curriculum. This component was then revised to include a simplified behavior-focused story. Community health workers reported difficulty balancing the required content of the integrated curriculum but were able to manage after the contents were reduced. The revised intervention is likely feasible to deliver to a group of pregnant and lactating mothers in a low-resource setting.
Al-Delaimy et al. (2014)	2012-2013	Developing and evaluating health education learning package (HELP) to control soil-transmitted helminth infections among Orang Asli children in Malaysia	Malaysia	Iraq	Public health (Health Education & Community Medicine)	Mixed-methods approach: literature review, community discussions & impact evaluation of an open-label controlled intervention trial	Knowledge about soil-transmitted helminth infections	The developed package consists of a half day workshop for teachers, a teacher’s guide book to STH infections, posters, a comic book, a music video, a puppet show, drawing activities and an aid kit. The package was well-received with effective contributions being made by teachers, children and their parents. The incidence rates of hookworm infection at different assessment points were significantly lower among children in the intervention school compared to those in the control school. Similarly, the intensity of trichuriasis, ascariasis and hookworm infections were found to be significantly lower among children in the HELP group compared to those in the control group ($P < 0.05$). Moreover, the package significantly improved the knowledge, attitude and practices (KAP) of Orang Asli people and the knowledge of teachers towards STH infections.

Callery et al. (2020)	2018	Engaging ethnic minority communities through performance and arts: health education in Cambodian forest villages	Cambodia	Thailand	Tropical Medicine	Qualitative evaluation (interviews)	Health information retention; assessing contextual-appropriateness	Respondents were able to recall the key health messages about malaria, antenatal care and infant vaccination. They also showed good awareness of malaria transmission and prevention and described how they enjoyed the events and appreciated the efforts of the project team. In isolated communities in Cambodia, a health education programme harnessing performance and arts engaged the whole community and its messages were readily recalled and prompted reflection.
Flax et al. (2014)	2011-2012	Integrating Group Counseling, Cell Phone Messaging, and Participant-Generated Songs and Dramas into a Microcredit Program Increases Nigerian Women's Adherence to International Breastfeeding Recommendations	Nigeria	US	Public Health Nutrition	Cluster randomised control trial evaluation of an intervention, interviews, and survey	Early breastfeeding initiation; exclusive breastfeeding	Among the clients who completed the final survey (n = 390), the odds of exclusive breastfeeding to 6 mo (OR: 2.4; 95% CI: 1.4, 4.0) and timely breastfeeding initiation (OR: 2.6; 95% CI: 1.6, 4.1) were increased in the intervention vs. control arm. Delayed introduction of water explained most of the increase in exclusive breastfeeding among clients receiving the intervention. In conclusion, a breastfeeding promotion intervention integrated into microcredit increased the likelihood that women adopted recommended breastfeeding practices. This intervention could be scaled up in Nigeria, where local organizations provide microcredit to >500,000 clients. Furthermore, the intervention could be adopted more widely given that >150 million women, many of childbearing age, are involved in microfinance globally.
Goodman and Dent (2019)	2014	A Story Grows in Rural Uganda: Studying the Effectiveness of the Storytelling/Story-Acting (STSA) Play Intervention on Ugandan Preschoolers' School Readiness Skills	Uganda	US	Psychotherapy	Randomised control trial and qualitative evaluation (caregiver interviews)	School readiness skills	Overall, participants benefited significantly from a story-reading activity with or without STSA. When examining both groups together (N = 121 post-intervention), school readiness skills significantly improved. Caregiver variables also predicted these three child outcome variables at baseline, suggesting that caregivers play a significant role in the development of their children's school readiness skills.

Gautam et al. (2017) Gautam and Curtis (2021)	2012-2013	Trial of a Novel Intervention to Improve Multiple Food Hygiene Behaviors in Nepal<br>Food Hygiene Practices of Rural Women and Microbial Risk for Children: Formative Research in Nepal	Nepal	UK	Tropical Medicine	Cluster randomised control trial evaluation of an intervention; Process evaluation and mixed-method analysis of an intervention adaptation process	Food hygiene behaviors	The five targeted food hygiene behaviors were rare at baseline (composite performance of all five behaviors in intervention 1%[standard deviation (SD)= 2%] and in control groups 2%[SD= 2%]). Six weeks after the intervention, the target behaviors were more common in the intervention than in the control group (43%[SD= 14%] versus 2%[SD= 2%], P= 0.02) during follow-up. The intervention appeared to be equally effective in improving all five behaviors in all intervention clusters. This study shows that a theory-driven, systematic approach employing emotional motivators and modifying behavior settings was capable of substantially improving multiple food hygiene behaviors in Nepal.
Ghosh et al. (2006)	2001	A community-based health education programme for bio-environmental control of malaria through folk theatre (Kalajatha) in rural India	India	India	Epidemiology (Malaria research)	Impact assessment (interviews assessing knowledge and behaviour) comparing intervention with control villages	Control of malaria	The exposed respondents had significant increase in knowledge and change in attitude about malaria and its control strategies, especially on bio-environmental measures (p < 0.001). They could easily associate clean water with anopheline breeding and the role of larvivorous fish in malaria control. In 2002, the local community actively co-operated and participated in releasing larvivorous fish, which subsequently resulted in a noteworthy reduction of malaria cases. Immediate behavioural changes, especially maintenance of general sanitation and hygiene did not improve as much as expected.
Kim et al. (2018)	2010-2014	Large-Scale Social and Behavior Change Communication Interventions Have Sustained Impacts on Infant and Young Child Feeding Knowledge and Practices: Results of a 2-Year Follow-Up Study in Bangladesh	Bangladesh	US	Nutrition	Cluster randomized nonblinded impact evaluation and comparison between more and less intensive intervention packages, via cross-sectional household survey	Infant and young child feeding knowledge and practices	In intensive areas, exposure to IPC decreased slightly between endline and follow-up (88.9% to 77.2%); exposure to CM activities decreased significantly (29.3% to 3.6%); and MM exposure was mostly unchanged (28.1–69.1% across 7 TV spots). Exposure to interventions did not expand in nonintensive areas. Most IYCF indicators in intensive areas declined from endline to follow-up, but remained higher than at baseline. Large differential improvements of 12–17 percentage points in intensive, compared with nonintensive areas, between baseline and follow-up remained for early initiation of and exclusive breastfeeding, timely introduction of foods, and consumption of iron-rich foods. Differential impact in breastfeeding knowledge remained between baseline

								and follow-up; complementary feeding knowledge increased similarly in both groups.
Kumar et al. (2018)	2011-2015	What it takes: evidence from a nutrition- and gender-sensitive agriculture intervention in rural Zambia	Zambia	USA	Food policy	Cluster-randomised, controlled, non-blinded, impact evaluation (cross-sectional household survey and child stunting measurements)	Child undernutrition (via women's empowerment, IYCF knowledge and practices and child anthropometry)	The RAIN project had positive effects on women's empowerment, IYCF knowledge, child morbidity and weight-for-height z-scores, but had little impacts on IYCF practices, and no impact on stunting. Strengthening programme implementation and fostering higher participation rates could support greater impacts on child nutrition outcomes.
Limaye et al. (2018)	2017	Nuestras Historias-Designing a novel digital story intervention through participatory methods to improve maternal and child health in the Peruvian Amazon	Peru	USA	Pediatrics	sequential exploratory mixed-method study design (qualitative: process of digital story curriculum creation; quantitative: acceptability)	Maternal mortality rates	According to the PhotoVoice workshops, pregnancy-related problems included: lack of partner support, domestic violence, early pregnancies, difficulty attending prenatal appointments, and complications during pregnancy and delivery. Over 30 stories on these themes were recorded. Seven were selected based on clarity, thematic relevance, and narrative quality and were edited by a professional filmmaker. The acceptability survey showed that local participants found the digital stories novel (M = 89.4%, F = 83.3%), relatable (M = 89.4%, F = 93.2%), educational (M = 91.5%, F = 93.3%) and shareable (M = 100%, F = 100%). Over 90% of respondents rated the digital stories as "Excellent" or "Good", found the videos "Useful" and considered them "Relevant" to their communities.

Manjang, et al. (2018) Manaseki-Holland et al. (2021)	2014-2015	Promoting hygienic weaning food handling practices through a community-based programme: intervention implementation and baseline characteristics for a cluster randomised controlled trial in rural Gambia. Effects on childhood infections of promoting safe and hygienic complementary-food handling practices through a community-based programme: A cluster randomised controlled trial in a rural area of The Gambia	The Gambia	The Gambia/ UK	Public Health/ Paediatrics	Cluster randomised control trial	Complementary-food contamination and disease rates	For the primary outcome, the rate was 662/4,351(incidence rate [IR] = 0.15) in control villages versus 2,861/4,378 (IR = 0.65) in intervention villages (adjusted incidence rate ratio [aIRR] = 4.44, 95% CI 3.62–5.44, p < 0.001), and at 32 months the aIRR was 1.17 (95% CI 1.07–1.29, p = 0.001). We found that low-cost and culturally embedded behaviour change interventions were acceptable to communities and led to short- and long-term improvements in complementary-food safety and hygiene practices, and reported diarrhoea and acute respiratory tract infections.
McMichael & Robinson (2016)	2008-2012	Drivers of sustained hygiene behaviour change: A case study from mid-western Nepal	Nepal	Australia	Geography	Qualitative case-study evaluation (interviews, group discussions, drawings and household observation)	Water and sanitation-related diseases	Findings indicate the intervention has supported development of new norms around hygiene behaviours. Key drivers of sustained hygiene behaviour were habit formation, emotional drivers (e.g. disgust, affiliation), and collective action and civic pride; key constraints included water scarcity and socio-economic disadvantage. Identifying and responding to the drivers and constraints of hygiene behaviour change in specific contexts is critical to sustained behaviour change and population health impact.

Nguon et al. (2018)	2016-17	Art and theatre for health in rural Cambodia	Cambodia	Cambodia	Epidemiology (Parasitology)	Description & Reflection on lessons learnt	Dissemination of key health messages and supplementing existing engagement activities conducted by local authorities	We learnt the following lessons from our experience in Cambodia: involving local people including children from the beginning of the project and throughout the process is important; messages should be kept simple; it is necessary to take into consideration practical issues such as location and timing of the activities; and that the project should offer something unique to communities.
Olaide (2010)	Not stated	Maternal mortality and theatre intervention: A case study of Oluyole	Nigeria	Nigeria	Performing Arts	Descriptive case study evaluation	Maternal mortality rates	The paper discusses the practice of theatre for development as an information and enter-educative medium to the people most of whom are western educationally disadvantaged.

Sanghvi et al. (2013) Sanghvi et al. (2016) Nguyen et al. (2019)	2010-2016	Tailoring communication strategies to improve infant and young child feeding practices in different country settings; Achieving behaviour change at scale: Alive & Thrive's infant and young child feeding programme in Bangladesh; Information Diffusion and Social Norms Are Associated with Infant and Young Child Feeding Practices in Bangladesh	Bangladesh Ethiopia & Vietnam	USA	International Development	Cross country comparison of intervention design, adaptation phases and outcomes; Process description of multi-method intervention design and scale-up phases and evaluation via preliminary comparisons of intensive vs non-intensive treatment groups; Household surveys - analysis of paths from intervention exposure to IYCF practices through networks, diffusion, and norms (multiple regression analysis)	Exclusive breastfeeding and complementary feeding practices	Key messages: Well-designed and well-implemented large-scale interventions that combine interpersonal counselling, community mobilization, advocacy, mass communication and strategic use of data have great potential to improve IYCF practices rapidly. Formative research and ongoing studies are essential to tailor strategies to the local context and to the perspectives of mothers, family members, influential community members and policymakers. Continued use of data to adjust programme elements is also central to the process. Scale-up can be facilitated through strategic selection of partners with existing community-based platforms and through mass media, where a high proportion of the target audience can be reached through communication channels such as broadcast media. Sustaining the impacts will involve commitments from government and capacity building. The next step for capacity building would involve understanding barriers and constraints and then coming up with appropriate strategies to address them. One of the limitations we experienced was rapid transition of staff in key positions of implementing agencies, in government leadership, donors and other stakeholders. There was a need for continued advocacy, orientation and teaching related to strategic programme design, behaviour change, effective implementation and use of data.

Sloand and Gebrian (2006)	1994-2005	Fathers Clubs to Improve Child Health in Rural Haiti	Haiti	US	Nursing	Literature Review	Child and family health	Public health practitioners continually face grave challenges when addressing the health care needs in less developed countries. One such case is Haiti, where crushing poverty, poor infrastructure, a failing economy, natural disasters, and chaotic sociopolitical conditions compound these challenges. Public health practitioners, including nurses, must seek creative, culturally-appropriate, low technologic approaches to improve the health of the children and families in the remote villages of Haiti. The institution and support of village fathers clubs is one approach that has been ongoing since 1994. Fathers meet together on a regular basis for health education, support, and community building activities. The curriculum is health-based and facilitated by nurses, with participation by young and old men alike. Participants and organizers believe that family and child health is improved as a result of the groups.
Thuita et al. (2015) Mukuria et al. (2016)	2010-12	Engaging fathers and grandmothers to improve maternal and child dietary practices: planning a community-based study in Western Kenya<br>Role of Social Support in Improving Infant Feeding Practices in Western Kenya: A Quasi-Experimental Study	Kenya	Kenya/ USA	Public Health	Quasi-experimental, non-equivalent comparison group design with pre- and post-testing (surveys, formative assessment and process evaluation);<br>Quasi-experimental, multi-method, multi-phase intervention description and evaluation (survey)	Maternal and child dietary practices; Complementary feeding practices	We surveyed 554 people at baseline (258 mothers, 165 grandmothers, and 131 fathers) and 509 participants at endline. The percentage of mothers who reported receiving 5 or more social support actions (of a possible 12) ranged from 58% to 66% at baseline in the 3 groups. By endline, the percentage had increased by 25.8 percentage points (P=.002) and 32.7 percentage points (P=.001) more in the father and the grandmother intervention group, respectively, than in the comparison group. As the number of social support actions increased in the 3 groups, the likelihood of a mother reporting that she had fed her infant the minimum number of meals in the past 24 hours also increased between baseline and endline (odds ratio [OR], 1.14; confidence interval [CI], 1.00 to 1.30; P=.047). When taking into account the interaction effects of intervention area and increasing social support over time, we found a significant association in the grandmother intervention area on dietary diversity (OR, 1.19; CI, 1.01 to 1.40; P=.04). No significant effects were found on minimum acceptable diet.

Winter et al. (2021)	2017-2018	The potential of school-based WASH programming to support children as agents of change in rural Zambian households	Zambia	USA	Civil and Environmental Engineering	Quasi-experimental, prospective cohort intervention impact evaluation (open and closed surveys)	Sharing of WASH-related messages (washing hands and using the latrine) with caregivers	Student knowledge increased significantly, but primarily among students in grade 1. Overall rates of students reporting that they shared messages from the curriculum with their caregivers rose from 7 to 23% ($p < 0.001$). Students in grade 4 were 5.2 times as likely as those in grade 1 to report sharing a WASH-related message with their caregivers (ARR = 5.2, 95% C.I. = (2.3, 8.9); $p < 0.001$). Although we measured only modest levels of student dissemination of WASH UP! messages from the school to the home, students in grade 4 showed significantly more promise as agents of change than those in grade 1.

Supplementary Table 6: Summary of analysis and review findings

Author (Date)	Co-production	Adaptation to context	Use of dramatic arts	What works, for whom, & in what circumstances? Was it sustainable?	Scalability/ Transferability
Akter et al. (2020)	Researcher-led process, with feedback from participants, community members and community health workers.	Three existing interventions were integrated and culturally adapted via researcher knowledge and situation analysis (direct observation).<br><br>Two models of delivery were piloted - one in 'courtyard groups' and the other in individual home visits. Used local school infrastructure for delivery.	Arts-based approaches were not part of the original intervention but added after community feedback. Health messages were conveyed through use of visual cues, interactive role play and storytelling.<br><br>Delivered to communities by community health workers.	Participants reported willingness to attend the sessions if they were extended for 1 year. Changes recommended included additional visual cues and interactive role-play activities to make the sessions more engaging, a simplified behaviour-focused story (rather than the concept of 'unhealthy thoughts'), and to reduce the amount of content.<br><br>The study did not collect long-term data.	Small scale limits generalisability.<br><br>A systematic participatory approach was taken in intervention design, and a revised intervention was proposed which was likely feasible to deliver to a group of pregnant and lactating mothers in a low-resource setting.<br><br>Group sessions were as effective as combined ones and therefore potentially more cost-effective and scalable.
Al-Delaimy et al. (2014)	Researcher-led process, with engagement of a range of academic disciplines (e.g. public health, parasitology, education), local public health and community development officials, as well as residents, community members and service providers.<br><br>Limited opportunities for feedback during or after delivery.	Local adaptation of health education messages, via literature review and direct observation (household survey) by researchers of the Orang Asli communities, responding to a history of 'failed' interventions in the region.	Posters/comic books, nursery rhymes and songs (lyrics on posters and songs on YouTube and school computers) and a puppet show were used alongside more traditional learning materials.<br><br>Delivered to child-participants by teachers, who were trained in relevant health content.	Knowledge and behaviour improved (with variable onset, longevity and extent), and school infrastructure was inclusive of range of children.<br><br>No long-term outcomes, beyond 6 months (where control group had lower infections, but below statistical significance threshold). Long-term funding unclear.	The intervention used teachers making it scalable, but how willing or able teachers are to help will vary between individuals and places. The arts elements depended on availability of school materials e.g. computers, so may not be reproducible in all contexts.

Callery et al. (2020)	Researcher-led and stakeholder-funded. Involvement of a range of stakeholders at different levels, including Deputy District Governor, commune and village leaders, primary school principals, health centre chiefs, the police chief and village malaria workers.	A existing intervention was adapted to local context via a large stakeholder orientation meeting (n=~50) at a community health centre, where the activities, target villages and topics for community engagement were discussed.<br><br>This was followed by additional local, informal adaptation in each village, through meeting with commune and village leaders, the principal of the local school and village malaria workers to discuss with them the activities and inviting the students to participate.<br><br>TV was used to promote the events and celebrate.	Intervention was delivered by a community engagement team and members of a professional theatre group experienced in community/ village drama.<br><br>Participatory art and theatre workshops were delivered to school children to prepare for a village performance, which included wider members of the community. The workshops with schoolchildren were facilitated by teachers, with parental consent.<br><br>The performance included games, singing and dancing, health quizzes, a fashion show (showcasing traditional costumes, dance and musical instruments) and a comedy sketch about malaria. The closing event involved speeches, competitions, performances from professional singers, local traditional dances and an amateur singing competition.	Qualitative interviews suggested that knowledge of health messages had improved, with general recall of messages but not all details. Some participants shared the messages with non-participants. Involvement of children was noted to boost investment of families and teachers (and to require mastery of health messages for the performance).<br><br>Ethnic minority groups were specifically engaged and asked to be special guests at arts events in other villages. Showcasing elements helped the participants and the audience to gain a sense of ownership of the performances and this element was particularly popular among the local Kaviet and Lao populations.<br><br>No long term outcomes reported. Unclear whether further funding would be required or available.	This intervention was an adapted reproduction of preceding interventions, so can be considered reproducible. No immediate barriers to scaling were noted.

Flax et al. (2014)	Researchers partnered with a US NGO (Partners for Development) and 4 local, Nigerian community-based organizations.<br><br>Community members were recruited into microcredit groups of 5-7 friends, neighbours, or relatives who guaranteed each other's loans.	The intervention was delivered within an established social/economic grouping - women's micro-credit groups - that met monthly.<br><br>It was unclear whether there was any local adaptation or feedback process to the intervention.	Small groups of residents living near each other (in the microcredit groups) were asked to perform one of the key messages in a song or dramatic form.	Knowledge and behaviour improved, and intervention groups did breastfeed more at 3 and 6 months.<br><br>No longer term outcomes documented.	The microcredit aspect of this intervention may be difficult without American developmental organisation support. Could also cause social disruption instead of solidarity if poorly managed. Other aspects are low-cost and use local organisations.
Goodman and Dent (2019)	Researcher-led process, whereby researchers identified the problem and designed the intervention then, using translators, trained the local project coordinators to deliver the intervention.	The team ensured that the intervention was low cost and used local school infrastructure for delivery.	Storytelling and story-acting were used in the intervention. The story development was entirely child-led and 'spontaneous', integrated into play.	No difference overall between intervention and control group (who did drawing instead of story-telling). Some evidence that lower-income students and girls benefitted more.<br><br>No long-term outcomes reported. It was unclear whether further funding would be required or available.	Storytelling is low-resource and but limited evidence of effectiveness. The use of local schools for delivery would be scalable.

Gautam et al. (2017) Gautam and Curtis (2021)	Researcher-led process, drawing on literature and theoretical models of behaviour change (Behaviour-Centered Design), but also engaging a range of stakeholders and local communities. Government and non-governmental organizations' advised that the intervention adapted from preceding interventions in Mali and India would be replicable and scalable in Nepal. A local creative team was assembled to design the intervention. Local women with a similar profile to Nepal's ubiquitous Female Community Health Volunteers (FCHVs) an intervention package was designed and delivered, with a local creative team with expertise in marketing, design, innovation, program development, and behaviour change.	The intervention design was informed by formative research conducted by 4 local research field data collectors and five local women from different castes/ethnic groups using handheld cameras, and analysed by the research team. Following this group discussions with community members and health officials.<br><br>Several pilots took place for feedback from those delivering the intervention and the communities. Final feedback was from government officials and NGO stakeholders.	Local rallies, folk songs, games, rewards, storytelling, drama, cookery demonstrations and "kitchen makeover parties" were explicitly linked to the motivational drivers of behaviour identified in the guiding programme theory (Affiliation, Disgust, and Status).	The intervention changed five key behaviours observed in formative research to improve the microbiological environment. Women who engaged undertook public pledging and were celebrated with a public display of photos. The intervention was effective in differing socio-economic settings, although it is unclear if there were inequalities within settings in the women who did and did not participate.<br><br>No long term outcomes observed.	That the intervention was equally effective across targeted behaviours and across clusters is a positive indicator that the improvements are reproducible. Health workers found that it was feasible to deliver the intervention on top of their existing workload, suggesting potential scalability.

Gale NK, *et al. BMJ Open* 2024; 14:e083093. doi: 10.1136/bmjopen-2023-083093

Ghosh et al. (2006)	Led by an intersectoral committee (involving ten governmental and non-governmental organisations), and delivered by local agents. Funded by the government, whose ministers provided visibility for the programme. Religious leaders contributed by offering free accommodation and hospitality for the period of one month as a token of solidarity in the fight against malaria. Local press and radio helped in wider dissemination of health education messages.<br><br>Local and regional organisations supported the intervention, e.g. the education department deputed five teachers and the Child and Women's Welfare Department deputed ten Anganwadi workers for one month.	Informal adaptation through a two-day visit from the intervention team prior to the final event.<br><br>Unclear if there were opportunities for feedback once the intervention had started.	Local educators, artists, religious leaders and community members collaborated to deliver the intervention.<br><br>Kalajatha is a popular, traditional art form of folk theatre depicting various life processes of a local socio-cultural setting . A local scriptwriter wrote 8 songs, two dramas and 4 rupakas (musical dramas) based on various aspects of malaria. These were then translated into skits using local dialects, musical styles and theatre traditions.<br><br>Thirty local artists (15 males and 15 females) from different occupational background were selected. Female artists were involved in the team, which resulted in better engagement from the women in the community.	Immediate behaviour changes were not evident but knowledge and attitudes of participants improved.<br><br>No long term outcomes were directly observed, but in a subsequent malaria prevention study via biocontrol in the area the year after this intervention, a dramatic improvement in malaria incidence was observed and in the study area people still refer to the Kalajatha events and the messages delivered to them, suggesting long term knowledge retention.	This intervention was implemented through and with existing local and regional organisations. The theatre performances themselves should not be highly expensive and require few materials.

Kim et al. (2018)	Donor-led initiative, working with researchers (from outside the country). The focus of the intervention shifted somewhat with new donors, who scaled up and adapted the programme. This is a top-down change (or feedback process).<br><br>The community-based infection and prevention control work was delivered by a international development organisation based in Bangladesh. Local health workers/volunteers/artists and regional/local infrastructures and organisations were used to deliver Infant and Young Child Feeding (IYCF) messages in regular meetings, community video and theatre shows, as well as radio and TV.	Broad consideration of context in intervention design (media, situation analysis, communications) concept testing, leading to creative development of prototype materials, (6) material pretests followed by revisions<br><br>The campaign implementation was subjected to ongoing monitoring, which was reported to lead to early and mid-course adjustments. However, evidence of bottom up changes/feedback incorporation in substantive content, or form of its delivery were not explicit.	Arts were only one component of a comprehensive programme of health promotion. Community video and theatre shows focused on infant and young child feeding. TV spots were broadcast nationally and regionally with intervention relevant messages.	This study offers evidence of decreased yet sustained impacts on IYCF practices even under modifications, suggesting program drift. Continued exposure infection prevention and control messages and sustained impacts on infant and young child feeding practices indicate lasting benefits from these interventions, as they underwent major scale-up and adaptations after termination of initial external donor support. At 2 y after endline, intervention exposure had decreased, but home visits by frontline workers remained moderately high in the intensive areas and significantly higher compared with the non intensive areas.<br><br>The programme is reliant on significant funding from external (non-governmental and internation) organisations, so sustainability is dependent on this continuing.	This project involved lots of government and non-government organisations. The practices of community arts-based health messaging is reproducible given low cost when working alongside existing organisations and with existing infrastructures.<br><br>In other contexts, where perhaps there was not be a broad political consensus on health priorities, expensive aspects of the current intervention may not be replicable, such as the TV ads. Reproducing this kind of comprehensive project at scale is challenging and demands significant collaboration.

Kumar et al. (2018)	International humanitarian organisation established partnerships with relevant government line ministries and with local implementing NGOs who were to deliver programme content across the different project components. Evaluation was conducted by International Food Policy Research Institute.	Used existing community health volunteers and women's groups, and existing government IYCF guidelines and materials to implement a gender-sensitive homestead food production programme with nutrition behaviour change communication.<br><br>Contextual issues were discussed in the paper and and initial gender analysis informed the intervention, but it is unclear whether there was any further adaptation informed by stakeholders.	Drama-based approaches were part of broader range of activities, including agricultural interventions (home gardening, providing seeds and equipment), and behaviour change components (group activities, radio messaging, posters, brochures) and women's empowerment (supply of fuel-saving stoves and fuel trees as labour-saving technologies). Community sensitisations were undertaken with assistance from drama groups, particularly around gender equality and its importance for improved nutrition. It is unclear whether these were participatory in delivery.	Positive effects were observed on women's empowerment, IYCF knowledge, child morbidity and weight-for-height z-scores, but little impact was seen on IYCF practices, and no impact on stunting. Areas that could be strengthened were programme implementation and fostering higher participation rates.<br><br>Long term outcomes were not observed.	The project was initially funded by international research organisations. It used local infrastructure and at times altered it, or offered incentives for participation (e.g. bicycles and/or agricultural inputs). The processes of adaptation to context (in design and delivery) are not very explicit.
Limaye et al. (2018)	Design of a digital story curriculum was participatory: the researcher ran three community-based PhotoVoice workshops with Community Health Workers and women who were invited by the CHWs; then they were involved in participatory digital storytelling activities. Stories were selected by the research team, who worked with a professional filmmaker and a professional midwife to develop these stories into a final digital story curriculum, where each story was combined with question-based teaching prompts.	The programme worked with existing community health workers, and the curriculum was iteratively developed with a variety of stakeholders.<br><br>There were limited opportunities later in the project for local storytellers to influence the final product (due to limitations of needing to process the digital stories in a city).	Photography (photo-voice) and community-based digital storytelling activities were used to inform the development of the curriculum. Participants were encouraged to think of personal stories and recorded themselves on mobile devices narrating their stories. Then participants did a storyboarding activity, in which they broke down the components of their story, and determined what types of photos would fit with each component of the story.	The digital story curriculum was highly acceptable to the target population, both men and women. They were considered novel, relatable, educational, and shareable.<br><br>Long term outcomes were not observed.	Solar-powered mobile phones with cameras are becoming cheaper and so the participatory process for designing a curriculum in this way is perhaps likely more replicable over time. Reproducibility was not investigated directly, but if able to utilise existing networks of local health workers and secure funding, there are no obvious issues.

Manjang, et al. (2018) Manaseki-Holland et al. (2021)	Local researcher-led, with wide consultation with health promotion agencies who were represented on a Local Scientific Advisory Committee in the Gambia (MOH, Unicef, WHO, University of the Gambia, National Nutrition Agency (NANA) and the MRC Gambia).<br><br>Locally, traditional communicators, community leaders and traditional birth attendants or other experienced women/ grandmothers were engaged. Women's achievements were celebrated individually (as MaaChampions) and communally (through villages achieving MaaChampion status).	The intervention was based on theoretical models of behaviour change and findings from previous similar intervention studies (in Nepal and India) and were adapted to the local context, based on formative research and consultation with stakeholders. The intervention material was translated into the three local languages (Mandinka, Wolof and Fula), field-tested with mothers and villagers and piloted iteratively by the intervention team.<br><br>TV was used to promote the events.<br><br>Women's networks were used to convey the messages 'onward' from the intervention, finding more volunteers. These women visited the families between the visits from the wider team to help establish the practices within the cultural norms of the community.	Performing arts (using culturally ingrained styles of drama and songs), animation, and stories.<br><br>Delivery team included traditional communicators (male and female, who use traditional African drumming, singing and acting), public health officers, a driver and a trained female volunteer, usually a traditional birth attendant (TBA).<br><br>Villagers reported fond memories of a 'joyous' programme with songs and activities of traditional performing artists, and they 'treasured' the achievements of MaaChampions in their family and community.	After intervention, behaviours improved (higher soap use in domestic kitchens and latrines) and there was a significant reduction in reported diarrhoea episodes and hospital admissions in the rainy season. No information was available about any inequalities in its effect.<br><br>Improved knowledge of the key behaviour messages was sustained at 6 months.<br><br>Qualitative data at 32 months suggested that mothers in intervention villages felt it their duty to inform mothers about complementary-food safety and hygiene practices when visiting other villages.	Cost-minimising tips were discussed toward replicability. Local structures and personnel used, with a low cost intervention.<br><br>This study builds on a smaller cluster RCTs of similar interventions in Nepal and India. This suggests the potential for reproducibility across different LMICs, and scope for scaling.<br><br>This low-cost intervention was not only acceptable to the mothers and the communities at large but was, at least in part, self-sustaining since new mothers reported practicing the behaviours at 32 months.

McMichael & Robinson (2016)	Researcher-led, in partnership with Australian and Nepal Red Cross. Two non-local researchers led the data collection with the assistance of a Nepali bilingual research assistant and two Nepali interpreters (one male, one female). Local residents were involved in some elements, e.g. children in participatory hygiene education and activities, group/committee formation (e.g. junior/youth Red Cross circles, women's groups, Water User Committees) and employment of local Village Health Motivators to conduct community-based hygiene education and home visits. Significant changes were made as a result of community feedback: for example, the original intervention employed Community-Led Total Sanitation triggering tools to create demand for latrines and improved hygiene (e.g. "walk of shame", flagging faeces) which were found to be 'difficult' and removed when all used the toilets. Positive motivational drivers were reported to be more effective.	Feedback was sought in focus group discussions and in depth-interviews. These were used to evaluate intervention impact. Ongoing program monitoring was noted but is unclear how this may have impacted adaptation beyond the informal adaptation through participatory activities.<br><br>Arts-based elements used as part of a broader intervention programme addressing different dimensions of the socio-economic and public health context: including provision of partially subsidised hardware; group/committee formation; hygiene and sanitation education; distribution of WASH information and education materials; employment of local Village Health Motivators to conduct community-based hygiene education and home visits; peer-led WASH awareness raising in schools/community; hygiene and sanitation campaigns; faeces calculation and fining of open defecation; and establishment of community funds that provide small loans for toilet construction.	Children and community members were engaged through the arts to raise awareness of WASH - through street drama and song.	Qualitative evidence showed changes in hygiene knowledge, behaviour and development of new social norms around hygiene. There was some anecdotal evidence of improvements in health.<br><br>Contextual limitations included the prohibitive cost of further latrine construction, and an inconsistent water supply which limited flushing and handwashing practice.<br><br>This paper presents qualitative reports on the interventions impact two years after beginning. The project's impact appears to have been supported by the diverse and intensive behaviour change activities that were repeated over a long period time by peers as well as community leaders.	Small-scale and intensive nature limits the applicability of these findings to large-scale WASH interventions.<br><br>Latrine construction could not persist due to the cost.

Nguon et al. (2018)	Led by local researchers, in partnership with the provincial health authorities, who selected and trained a local drama group to deliver the intervention based on a competitive process.<br><br>Community members were involved through attending the workshops, which led to the local tailoring of content for the public performances, and in quizzes/competitions.<br><br>High trust and synergy was reported between performers and the community.	This project (a pilot science-arts initiative) supplemented existing engagement activities conducted by local authorities and a community engagement team supporting a malaria elimination study (clinicaltrials.gov NCT01872702).<br><br>There was no formative research or formal adaptation process, but workshops were held in each village by the drama team, to adapt the performance using real stories of locals' experiences with malaria.	In each village there was a two and a half-day workshop led by a team consisting of local drama groups and health workers. There was a 'memorable' public performance on the third evening, incorporating local legends and real stories from the community. Community members, including children, were engaged using comedy, drama, drawing, singing competitions and quizzes about the intervention messages with prizes offered.	The drama storylines and health messages were remembered across the groups, and there was media interest in the project.<br><br>Attendance was lower proportionally in larger villages.<br><br>No long-term outcomes reported.	Intervention attracted large audiences in the villages, and offered something unique to remote, rural villages - especially involvement of professionals and the opportunity for children to be performing on stage alongside professional actors and seeing themselves in newspapers and on television. This is likely reproducible in other contexts, but not necessarily scalable as it would need to be tailored.

Gale NK, *et al. BMJ Open* 2024; 14:e083093. doi: 10.1136/bmjopen-2023-083093

Olaide (2010)	Researchers, local drama groups, participants, stakeholders included government, health/medical and environmental establishment figures were involved in the design process.<br><br>The drama was intended to become the 'theatre of the people, by the people and for the people' (based on the Boalian concept of forum theatre) which aims not to find the 'best' solution but to produce a variety of options that could be used in the type of situation.	Communal research was conducted early in the process and identified that the most appropriate approach would be an 'inside-out approach' which would enable facilitators to interact within the community, gaining their trust and confidence and identify a 'joint panacea to the problems'.<br><br>The first step was the involvement of an existing and popular community-based theatre group in the dramatic endeavour data analysis, scenario development, improvisation rehearsal, performance and post-performance discussion. Also involved are some individuals within the community in the play presentation and the relevant themes were discussed after the performance in a live Q&A the programme took three days to put together.	Theatre performances, storytelling, later involved were drumming, singing and dancing which also made use of the songs that were known to them, most especially the nursing mothers and the pregnant women.<br><br>The performance comprised of 5 different but complementing sketches that were based on the causes and preventive measures of maternal mortality. The sketches were spiced up and linked together with different maternal songs that were folkloric, especially to the community members of the local government area.	The study appears to have caught the attention of and galvanised the community around issues of maternal mortality based on the qualitative reports cited in the paper.<br><br>The cost as with other local theatre groups should be low.<br><br>Long term outcomes were not observed.	No obvious barriers to scalability or reproduction were noted given low cost of theatre and success of other similar interventions.

Gale NK, *et al. BMJ Open* 2024; 14:e083093. doi: 10.1136/bmjopen-2023-083093

Sanghvi et al. (2013) Sanghvi et al. (2016) Nguyen et al. (2019)	Partnerships with stakeholders were vital to expand the program's reach and to sustain program activities after initial funding ended. Partners were engaged in the research and design process to give implementing partners ownership of the strategy.<br><br>Government staff were trained by national and master trainers who were in turn trained by Alive and Thrive (A&T). The programme also launched a mass media campaign through inserts in newspapers (local journalists).	In each of the countries, Alive & Thrive convened stakeholders to review research to: (1) identify a few priority IYCF practices that would be the focus of the activities; (2) assess the degree to which select behaviours were feasible from family members' point of view and identified the conditions.<br><br>For all three countries, ProPAN methods were adapted and supplemented with additional tools, such as focus group discussions to gather information from frontline heath workers, fathers, grandmothers, and community leaders. The situational analysis in each country involved stakeholder consultations and reviews of existing data sets and reports to identify strategic choices and gaps in the data. Media audits, conducted by media research firms, guided two decisions: how much effort to devote to implementing or improving the Code of Marketing of Breast-Milk Substitutes, and what media channels to use for reaching different audience segments.	In all three countries, the mass media campaign containing emotionally appealing mini-dramas with various messages to spread the perception of recommended practices as normative was designed for a run of at least 2 years to build up audience recognition and recall. The campaign's television ads were loaded onto DVDs and shown on screens in outlying areas with low electricity, using portable generators, and the frontline worker training included a session using the television spots. Rural communities in media dark areas engaged through other mediums such as theatre.	Local adaptation, engagement of multiple stakeholders and scaling up were key elements of these projects. Combining face-to-face interventions for mothers with social mobilization and mass media was effective in improving IYCF practices.<br><br>The broad campaigns may both help and hinder sustainability. Helpful in that broad audiences through information diffusion can help transform norms quickly for many. A hinderance in the complexity of messaging with locally adapted schedules, content as well as their organisation. Partnerships with local organisations potential helped sustain the programmes after initial funding.	The campaign was delivered at a large scale, reaching an estimated 8.5 million. Scale-up was achieved through streamlining of tools and strategies, government branding, phased expansion through a local non-governmental implementing partner with an extensive community-based platform – and nationwide mainstreaming through multiple non-governmental organization and government programmes. The process has also been replicated in three countries. Without external (international) funding the scale of this study may not be replicable.

Gale NK, *et al. BMJ Open* 2024; 14:e083093. doi: 10.1136/bmjopen-2023-083093

Sloand and Gebrian (2006)	Haitian Health Foundation (HHF), which is supported by both private donations and public funding, instituted fathers clubs in 1994. The fathers meet regularly, usually weekly or every 2 to 3 weeks, to learn about a wide range of child and family health topics and the related household- and family-level solutions. They discuss how to support their wives in the care of their children. They share family problems and help each other when needed. Meetings were described as 'very participatory'. The meetings are led by the fathers but attended by health agents (community health workers) and often a nurse who supervises the health agent. Together, they provide health education and support to the fathers.	Topics are decided by group discussion and fathers also engage in wider local infrastructure/project issues.	The focus of meetings is usually a child or family health issue, and discussion, songs, and skits are used to enhance learning. To reach out beyond the group, they help organise village health fairs with mothers' clubs or village health committees.	Fathers reported changes in knowledge, behaviour, social norms and skills, as well as health benefits (although these were not confirmed in observational studies). Father involvement is higher than expected based on other areas in Haiti.<br>Fathers report believing that the health and well-being of all the village children is the shared responsibility of all the adults in the village.<br>It is unclear if there are inequalities in access or participation for fathers.<br><br>The Fathers Club project has been running since 1994 in Haiti, so can be considered very sustainable in this context. However, long term outcomes were not formally evaluated/reported.	Low resources and low development context of this intervention imply high reproducibility across contexts. The use of existing health agents improves scalability also, however in contexts without a private/voluntary health foundation to provide support and organise reproducing and scaling may be more difficult.

Thuita et al. (2015); Mukuria et al. (2016)	International aid agency funded the project, which engaged volunteer community health workers (CHWs). These CHWs form community health committees and elect their leaders. In functional units, CHWs and committees are actively providing and overseeing community health surveys.<br><br>Each dialogue group selected one of its members to serve as the group mentor, who was trained and assisted by a volunteer CHW. Feedback was provided as well. Before each meeting, mentors selected the discussion topic based on member interest and then facilitated discussions and activities with group members.<br><br>Tea allowance was provided to all groups during meetings, however, fathers preferred money (100 ksh/ 1 USD).	Formative research designed key messages for fathers and grandmothers in households in western Kenya. Peer dialogue groups were used to facilitate behaviour change among fathers and grandmothers by helping them gain new knowledge, share experiences and reflections, and apply communication and problem-solving skills that in turn would facilitate behaviour change in mothers.<br><br>Existing health facilities were used to host events. Trained CHWs conducted home visits with study participants in both the intervention and comparison groups and shared oral and written complementary feeding and maternal nutrition messages. In the intervention areas, fathers or grandmothers were invited to participate in this visit.	Dialogue-based groups were used to engage infants' fathers and grandmothers to support optimal infant feeding practices. Group members participated in role plays, problem solving activities, storytelling, and cooking demonstrations; grandmothers also composed songs promoting recommended practices. They showcased what they were learning through songs, skits, dances, and testimonials from fathers, mothers, grandmothers, CHWs and children, at Community Bazaars.	Family-centred approach, through engaging fathers and grandmothers of infants to improve their knowledge of optimal infant feeding practices and to encourage provision of social support to mothers could help improve some feeding practices. It is unclear whether all groups were engaged in the activities. Arts were used for community mobilization activities.<br><br>The use of local infrastructure is promising for sustainability of the intervention. The bazaars are low cost also.	Low cost and resource usage. Builds on existing infrastructure.

Winter et al. (2021)	World Vision and Sesame Workshop collaboratively developed the WASH UP! program. WASH UP! combines a 12-week educational curriculum created by Sesame Workshop targeting students in grades 1-4 with school WASH infrastructure improvements carried out by World Vision. Teacher training is conducted by Sesame Workshop over the course of 3 days, and materials distribution and infrastructure improvements are funded and implemented by World Vision. The curriculum includes 12 sessions, each of which is approximately 30-40 min long and uses child-friendly pedagogical strategies such as play-based learning.<br><br>The twelve sessions were designed to be conducted before, during, or after normal school hours, at the discretion of the teacher. A learning object was distributed to a random sub-set of five of the 12 schools. Teachers provided the learning object to students with the instruction to take it home and discuss a key message about handwashing from the WASH UP! curriculum with their caregivers.	helping families to establish and maintain handwashing stations close to the place of child feeding rather than focusing on the message 'wash your hands with soap'.<br><br>Mass media and community mobilization were used to create an enabling environment by changing perceptions about using soap as the norm. Communication materials and a TV spot focused on convenience - establishing a handwashing close to the place of food preparation. A training session on handwashing stations was also inserted in the national IYCF training manual.	Activities include listening to the teacher read from a story book, playing interactive games, structured discussion of key curriculum messages, and watching two 10-min videos.	No evidence of effectiveness of children as agents of change.<br><br>Disruption to teacher's schedules and workloads were noted, this also was an image due to potential reputational damage for World Vision within such communities where they work. This necessitated some adaptation of scheduling of intervention delivery. Projectors and videos were included as part of the curricular materials, but three of the twelve schools did not have electricity, making one of the twelve sessions much more challenging to administer	Low cost, low resource (is theatre less expensive than video? Likely more engaging, more easily made participatory and local)
