# Supplementary material for: Coproduced, arts interventions for nurturing care (0–5 years) in low-income and middle-income countries (LMICs): a realist review
Source: BMJ Open. 2024 May 17;14(5):e083093. doi: 10.1136/bmjopen-2023-083093 (PMC11103195; doi:10.1136/bmjopen-2023-083093)
Supplement: Author's manuscript [file bmjopen-2023-083093.draft_revisions.pdf]

BMJ Open

BMJ Open is committed to open peer review. As part of this commitment we make the peer review history of every article we publish publicly available.

When an article is published we post the peer reviewers' comments and the authors' responses online. We also post the versions of the paper that were used during peer review. These are the versions that the peer review comments apply to.

The versions of the paper that follow are the versions that were submitted during the peer review process. They are not the versions of record or the final published versions. They should not be cited or distributed as the published version of this manuscript.

BMJ Open is an open access journal and the full, final, typeset and author-corrected version of record of the manuscript is available on our site with no access controls, subscription charges or pay-per-view fees (http://bmjopen.bmj.com).

If you have any questions on BMJ Open's open peer review process please email
info.bmjopen@bmj.com

Co-produced, arts interventions for nurturing care (0-5 years) in Low- and Middle-Income Countries (LMICs): a realist review.

Journal:	*BMJ Open*
Manuscript ID	bmjopen-2023-083093
Article Type:	Original research
Date Submitted by the Author:	20-Dec-2023

Complete List of Authors:	Gale, Nicola; University of Birmingham<br>Ahmed, Kalim; University of Birmingham<br>Diarra, Niélé ; University of Bamako<br>Manaseki-Holland, Semira; University of Birmingham College of Medical and Dental Sciences, Department of Public Health, Epidemiology and Biostatistics<br>Asamane, Evans; University of Birmingham<br>Sidibé, Cheick; University of Bamako<br>Touré, Ousmane; University of Bamako<br>Wilson, Michael; Loughborough University<br>Griffiths, Paula; Loughborough University, School of Sport Exercise and Health Sciences

Keywords:	Public health < INFECTIOUS DISEASES, Community child health < PAEDIATRICS, Systematic Review

SCHOLARONE™ Manuscripts

I, the Submitting Author has the right to grant and does grant on behalf of all authors of the Work (as defined in the below author licence), an exclusive licence and/or a non-exclusive licence for contributions from authors who are: i) UK Crown employees; ii) where BMJ has agreed a CC-BY licence shall apply, and/or iii) in accordance with the terms applicable for US Federal Government officers or employees acting as part of their official duties; on a worldwide, perpetual, irrevocable, royalty-free basis to BMJ Publishing Group Ltd ("BMJ") its licensees and where the relevant Journal is co-owned by BMJ to the co-owners of the Journal, to publish the Work in this journal and any other BMJ products and to exploit all rights, as set out in our licence.

The Submitting Author accepts and understands that any supply made under these terms is made by BMJ to the Submitting Author unless you are acting as an employee on behalf of your employer or a postgraduate student of an affiliated institution which is paying any applicable article publishing charge ("APC") for Open Access articles. Where the Submitting Author wishes to make the Work available on an Open Access basis (and intends to pay the relevant APC), the terms of reuse of such Open Access shall be governed by a Creative Commons licence – details of these licences and which Creative Commons licence will apply to this Work are set out in our licence referred to above.

Other than as permitted in any relevant BMJ Author's Self Archiving Policies, I confirm this Work has not been accepted for publication elsewhere, is not being considered for publication elsewhere and does not duplicate material already published. I confirm all authors consent to publication of this Work and authorise the granting of this licence.

For peer review only

TITLE

Co-produced, arts interventions for nurturing care (0-5 years) in Low- and Middle-Income Countries (LMICs): a realist review.

ABSTRACT

Community-based arts interventions have the potential to support contextually relevant nurturing care programmes and policies that adapt to different settings. Understanding the distinctive features of using the arts in local, culturally-specific ways in Low and Middle Income Countries (LMICs); how this varies by context; and gaining a better understanding of the perspectives on desirable outcomes for communities is important evidence that this review generates. We conducted a realist review of papers that covered outcomes related to child health or development (0-5years) AND arts-based approaches AND community-based, participatory approaches AND based in LMICs using a range of databases and other networks. The included papers reported 18 unique interventions. A coding system was developed covering context, intervention, outcomes, mechanisms, study, sustainability, transferability, and scalability. Interventions covered 14 countries, with evidence lacking for South America, Arab countries, and parts of Africa. Lead authors came from mostly clinical science-based disciplines and from institutions in a different country to the country/countries studied. Intended outcomes from interventions included clinical, health systems/organisation, changes in practices/behaviours/knowledge/attitudes, and wider social and educational goals. We identified three demi-regularities (semi-predictable patterns or pathways of programme functioning): participatory design based on valuing different sources of expertise; dynamic adaptation of intervention to context; and community participation in arts-based approaches. Our findings suggest that arts-based, nurturing care interventions have greater potential when they include local knowledge, embed into existing infrastructures, and there is a clear plan for ongoing resourcing of the intervention. Studies with better documentation of the

For peer review only - http://bmjopen.bmj.com/site/about/guidelines.xhtml

lessons learned, regarding the intervention delivery process and the power dynamics involved, are needed to better understand what works, for whom and in which contexts.

STRENGTHS AND LIMITATIONS OF THE STUDY

- Systematic search of the international literature ensured good coverage of the field
- Multi-disciplinary team authorship ensured realist synthesis methods could capture what works, for whom and in what circumstances.
- Review limited to English Language journal articles, so may have regional biases and positive results publication bias
- Inclusion of studies that report interventions meant that relevant contextual or conceptual paper, books or chapters, more common in the arts and humanities disciplines were not included.

MAIN BODY

1. Introduction

This paper reports the findings from a realist review and synthesis of the published and grey literature on community-based arts interventions targeted at improving health and development for 0–5-year-olds in Low- and Middle-Income Countries (LMICs). It identifies (i) possible intervention mechanisms focusing on the distinctive feature of using the arts in local, culturally specific ways; (ii) relevant issues of contextual difference and (iii) a broad perspective on desirable outcomes for communities, rather than focusing solely on medical outcomes. Our findings aim to inform policy and intervention design and implementation in this field, by identifying what works, for whom and in what circumstances, and what elements are likely to make interventions more sustainable, transferable, or scalable.

For peer review only - http://bmjopen.bmj.com/site/about/guidelines.xhtml

2. Background

2.1 Nurturing care:

UNCEF's nurturing care framework provides a structure through which countries or communities can support parents and caregivers to provide an environment that ensures optimal health, nutrition and development for infants and young children and in which these individuals can live free from threats and have opportunities to develop and learn using responsive interaction (Britto et al., 2017). More than 5 million children did not survive to their fifth birthday in 2020 (UNICEF, 2021). Furthermore, 43% (over 250 million) infants and young children in LMICs do not achieve their developmental potential for reasons including poverty, undernutrition and a lack of appropriate responsive and nurturing care and simulation (Lu et al., 2016; Richter et al., 2017). Appropriate nurturing care has the potential to reduce mortality and morbidity, such as from diarrhoea or respiratory diseases, reduce poor nutritional outcomes, and to improve early childhood development, safety and security of infants and young children and improve progress towards meeting the sustainable development goals (WHO 2018). To support the core elements of the nurturing care framework, programmes and policies are needed that are contextually relevant and take account of the requirements and different needs of all communities, because nurturing care is heavily embedded in local cultures and influenced by local political and economic circumstances.

Community-based arts interventions, such as projects working with traditional performing arts practitioners, have the potential to be contextually relevant and adapt to different settings. Understanding the distinctive features of using the arts in local, culturally-specific ways; how this varies by context; and gaining a better understanding of the perspectives on desirable outcomes for communities is important evidence that will come from this review to better understand how to support optimal nurturing care practices in different contexts.

2.2 Power and participation:

For peer review only

For peer review only - http://bmjopen.bmj.com/site/about/guidelines.xhtml

Community-based arts interventions are often co-produced. The term co-production is highly debated, but broadly refers to a collaborative and reciprocal process of exchange between actors of differing backgrounds, so as to generate outcomes uniquely embedded in the different perspectives shared (Durose et al., 2022). There are often significant challenges in implementing co-production in practice, such as knowledge and power asymmetries between those collaborating. Often power dynamics in these collaborations shift away from local expertise to professional expertise meaning that not all voices are heard (Egid et al., 2021; Yassi et al., 2016; Bamuturaki, 2016; Goncalo and Duguid, 2012; Bechtoldt et al., 2010; Schulz et al., 2003). Such power dynamics may produce interventions which are not sensitive to the specific issues, barriers and affordances of the context in which the intervention is implemented.

Many have argued that to overcome challenges and realise the purported benefits of co-production, collaborators must move beyond a ‘tick box’ or process-driven approach to involvement to partnerships that enable partnership synergy (Bamuturaki, 2016), which is built through mutual trust and understanding between parties and takes into account contextual factors (Jagosh et al., 2015; George et al., 2015). Careful consideration of how interventions may draw on and influence local infrastructures and relationships is also important, to build sustainable collaborations (Kohrt et al., 2018; Greenhalgh et al., 2016).

2.3 Arts-based approaches:

The Applied Arts refers to work that seeks to use the arts as a tool to be applied to a social issue. Other commonly used terms in the literature are Arts for Development, Arts for Social Change or Participatory Arts. Arts-based interventions are usually carried out in group settings where they are used to undertake collective processing and/or for the communication of new information such as health advice, e.g. through dramatic or musical performances, bazaar event days with activities, competitions and prizes. Applied Arts approaches are distinct from therapeutic approaches, such as Art Therapy, which are associated with improving individual health directly.

For peer review only

For peer review only - http://bmjopen.bmj.com/site/about/guidelines.xhtml

When practiced through local arts traditions, an intervention is embedded into local culture rather than imposed from outside, which is typically thought to make the intervention more relevant, coherent and engaging to community members as well as to empower and uplift by centring and celebrating existing community strengths (Stewart et al., 2022), particularly when performed by friends, family or neighbours. When both performer and audience are involved in a live performance it can act to bind communities with a sense of togetherness, increased trust and common goals (Stewart et al., 2022; Faigan and Stein, 2014). Such dramatic performances are often combined in community bazaar events with other ongoing arts activities, performances, competitions and quizzes with prizes to increase attention and attendance on the event day or days, to maintain interest throughout the event and provide more opportunities for more people to involve themselves.

Systematic reviews of arts-based interventions are lacking, with evidence spread across disciplines and perspectives and with aims differing between arts- and science-based initiatives (Logie et al., 2019; Torrissen and Stickley, 2018; Gallant et al., 2017; Faigan and Stein, 2014, Putland et al., 2008; Rossiter et al., 2008). Arts scholarship tends to focus less on evaluating interventions *per se*, and more on the artistry or process of artistic performance or creativity, or the role of artistic practice in interrogating concepts or cultures (e.g. Baxter & Low, 2017, SExtou).

2.4 Evaluating complex interventions and the importance of context:

Realist evaluation methodologies aim to ensure that context is incorporated in any analysis of effectiveness, using the refrain, ‘what works, for whom, and in what circumstances?’ (Pawson and Tilley 1997; Jagosh et al., 2013, Wong et al., 2013). Interventions that seek to address issues of improving nurturing care are often complex, addressing many layers of social and cultural practice, and influenced by political and economic context. Oversimplistic assessments that deem such interventions ‘effective’ or not are problematic. The nuanced realist evaluation of arts-based nurturing care interventions in LMICs provided in this review will provide evidence to inform policy and intervention design to improve nurturing care in some of the world’s most vulnerable contexts.

For peer review only - http://bmjopen.bmj.com/site/about/guidelines.xhtml

It will also explore the sustainability of interventions and generalisability of findings, and specifically whether interventions might be reproducible in other contexts (usually with some adaptations to context), or scalable to a wider population.

3. Research questions

i. What kinds of co-produced, arts-based, nurturing care interventions have worked in LMICs, for whom and in what circumstances?

ii. To what extent are successful interventions sustainable, transferable, or scalable?

4. Methods

Defining the scope of the review:

We worked with a team in the UK and Mali, including the intended users of the review, to clarify focus and prioritize research questions. We presented our initial ideas and later our preliminary findings to the [anonymous] project steering committee. [Anonymous] is an ongoing study with collaborations between institutions in Mali and the UK examining the impact of an intervention in Mali for nurturing care, with significant arts-based elements (Anonymous ref). Our preliminary objectives for this review were to identify examples of arts-based interventions co-produced between communities affected by poor child health, nutrition, and development outcomes and those involved in the public health/health promotion sector in LMICs and/or research organisations; to develop an understanding of the theoretical frameworks that inform these interventions or can help explain their effects, and to understand the mechanisms that contribute to success for these interventions. As the review progressed, we introduced a second research question focused on the long-term potential of these sorts of interventions to have an impact within the original context and in other similar ones. The findings from this review have been used to inform the data collection and analysis strategy for the primary empirical research.

Search strategy:

For peer review only - http://bmjopen.bmj.com/site/about/guidelines.xhtml

We searched for peer-reviewed journal articles that covered outcomes related to child health or development (0-5years) AND arts-based approaches AND community-based, participatory approaches AND based in LMICs (see Table 1: Search Terms).

These terms were used to search EBSCO, OVID, ProQuest, Scopus, Web of Science and Child Adolescence and Education Studies databases. This generated 940 unique results. After an initial screening of titles and abstracts, 175 results remained. Full papers were then screened by XX and XX independently, then discussed by members of the wider team. Sixteen met our inclusion and exclusion criteria (see Table 2: Relevance criteria for Inclusion/exclusion). The gradual refinement of these criteria helped focus the research question and ensured coherent conceptualizations of the research area between research team members. Additionally, 7 papers from our informal search and consultation with the wider reference group were identified which met these inclusion and exclusion criteria and were added to our sample. Of these papers, 18 unique interventions were identified (some were reported across multiple papers). The included interventions and the papers reporting them are summarized in Table 3.

Data Extraction:

To address the first research question, the team developed an initial coding framework:

- **Context:** Country-Region, Socio-Cultural, Economic-Developmental, Legal-Political, Geographical-Physical
- **Intervention:** Agents, Co-production, Arts-based Methods, Dates, Design, Implementation
- **Outcomes:** Impact on Primary Outcome; Impact on Secondary Outcomes, Unintended outcomes/consequences.
- **Mechanisms:** Observed-Evidenced; Theorised
- **Study:** Aims, Constructs-Variables, Date, Limitations, Sampling, Theoretical Framework, Study design/Methods, First Author Location

For peer review only - http://bmjopen.bmj.com/site/about/guidelines.xhtml

- **Paths:** Interaction between Context/Intervention, Context/Outcomes, Intervention/Outcomes, & Context + Intervention → Outcomes

The 'paths' extraction was based on anything in the paper that spoke direct to the issues of the Context-Mechanism-Outcome configuration (Pawson and Tilley 1997), even if a realist approach was not used. After agreeing the approach, papers were coded and data were extracted independently by XX and XX, who then had a series of discussions to resolve differences.

To address the second research question, a second round of data extraction was conducted by two different authors XX and XX on reported or potential issues of **sustainability**, **transferability**, and **scalability** for the interventions.

Data Synthesis:

First, we worked to compare and contrast the different interventions and the evaluation methods used, with specific reference to the ways in which the interventions focused on the key concepts of our research question, i.e. 'co-production' and 'community arts-based'. Second, we looked for demi-regularities in the data (Sayer 2000) in order to generate a set of propositions about the social mechanisms at play in these interventions. We identified three themes for further exploration: developing sustainable partnerships; tailoring interventions to local context, and using arts-based approaches that engage with social norms. Third, we appraised each intervention with a series of questions centred on these three themes (detailed in Table 4), in order to answer the question 'what works, for whom and in what circumstances?' Finally, we considered whether the interventions were reported to be or likely to be sustainable, transferable and/or scalable.

5. Description of included papers

Twenty-three papers were included in the review, which reported findings from 18 separate interventions (Table 3). In some cases, formative research or baseline characteristics were covered

For peer review only - http://bmjopen.bmj.com/site/about/guidelines.xhtml

separately from evaluations of the effectiveness of the interventions resulting in multiple papers per intervention.

Global Coverage

These interventions cover 14 different countries, with the majority focussing on the Asia Pacific region (Bangladesh (n=3), Cambodia (n=2), Haiti, India, Malaysia, Nepal (n=2), and Vietnam), followed by the African region (Ethiopia, Kenya, The Gambia, Nigeria (n=2), Uganda, and Zambia (n=2)), and one paper from South America (Peru). This shows that the evidence base is not distributed equally in regions hosting LMICs and that the English-language literature is not providing much evidence for the South American or Arab regions. There are also large parts of Africa without evidence, most notably the Western and Southern regions, most likely due to the searches being conducted in English.

Authorship Location and Disciplines

Given the focus on co-production in the review and the interdisciplinary nature of the interventions, there was a notable imbalance in the pattern of authorship. It was often unclear whether the projects had been initiated by those leading the publication or by others, such as local teams, but out of 23 papers, 17 have first authors affiliated to an institution in a different country to the country or countries being studied in the paper. In addition, most academic outputs were led from the health sciences, such as Psychotherapy, Public Health, Infectious Diseases, Nutrition, Health Education and Community Medicine, Epidemiology, Tropical Medicine, Nursing, Obstetrics and Gynaecology, Food Policy, Paediatrics, Maternal and Child Health, and Nutrition. Only one paper is led by researchers in the arts and humanities (Performing Arts) and two by social scientists (International Development, Geography). One paper was led by a team in Civil and Environmental Engineering.

Intended outcomes

For peer review only - http://bmjopen.bmj.com/site/about/guidelines.xhtml

For peer review only

There were a range of different intended outcomes from interventions, from the clinical (e.g. maternal mortality rates, malaria rates, anthropometry), the organisational (e.g. utilisation of services), to changes in practices/behaviours (e.g. changed dietary practices, exclusive breastfeeding, sharing of messages within families), and changes in knowledge or attitudes (e.g. dissemination of messages, health information retention) and wider social and educational goals (e.g. women's empowerment, school readiness). To a large extent, these were reflective of the disciplinary interests of the authors.

Evaluation methods

A range of qualitative and quantitative methods were used to evaluate the interventions, including cluster randomised controlled trials or impact assessments (non-blinded), quasi-experimental studies, a prospective cohort study, pre-post outcome evaluations (with or without control), cross-sectional surveys, interviews, focus groups and digital story creation. In a few cases, the focus was on descriptive case studies or process descriptions, rather than on evaluating outcomes.

6. Findings

Given the wide range of intended outcomes and evaluation methods used, we did not attempt to classify in any categorical way whether or not an intervention had been effective, rather we attempted to unravel whether the papers were able to say something about 'what works, for whom and in what circumstances' and whether improvements could be sustained. We did this by identifying and analysing context-mechanism-outcome configurations from information that was available in the papers. We identified three main demi-regularities: participatory design based on valuing different sources of expertise; dynamic adaptation of intervention to context; and community participation in arts-based approaches. These three pathways were unevenly realised across the different interventions described and evaluated in the papers, but can serve as a basis for future studies, or for those designing new or adapting interventions to reflect on. The details of how

For peer review only - http://bmjopen.bmj.com/site/about/guidelines.xhtml

these themes were reflected in each of the interventions included in the review are detailed in Table 5.

1: Participatory design based on valuing different sources of expertise

While our inclusion criteria required some element of 'co-production', there was a huge range of approaches, including those that were clearly researcher-led, with limited involvement of local people, local artists or other stakeholders, those that were driven by the interests of funders, particularly NGOs, and those where local people, local artists or local researchers drove the projects forward. In some cases, the co-production elements were primarily enacted during the design stages (e.g. Gautam et al. 2017), while others were mainly at the implementation stage through the adaptation of an existing intervention to local context (e.g. McMichael & Robinson, 2016). A further model provided continuing opportunities for communities to reflect and answer and input to intervention messages and materials throughout the length of the programme (e.g. Olaide, 2010).

Co-production models ranged from a very light touch co-production model to co-production that started at the beginning of the project and followed throughout the programme of activities. At the lighter end was a model where community health volunteers talked with local communities in discussion groups to get their involvement in the intervention, but where the intervention was designed externally to the community (e.g. Thuita et al. 2015). Other lighter touch co-production models included a programme of activities developed outside of the community but delivered with local artists/ drama groups to engage communities in the production of the intervention success (e.g. Nguon et al. 2018) or a model where an existing intervention is adapted with local community input (e.g. Manjang et al. 2018). At the other end of the spectrum, some projects used co-production models that started from the beginning of the project to design the intervention in partnership with the community (e.g. Gautum et al. 2017) or where communities develop the materials and stories that drive the intervention's focus messages (e.g. Limaye et al. 2018), or where the intervention was framed around collective learning between artist and the community (e.g. Olaide 2010).

For peer review only - http://bmjopen.bmj.com/site/about/guidelines.xhtml

There were some limitations in the reporting of co-production approaches. Often there was very little critical reflexive elaboration about the power structures in which the interventions were being designed and implemented, or what the challenges and benefits of harnessing community power structures were (such as who was included and who was not in the process). Another area that was underdeveloped was to what extent the resourcing of the intervention or the evaluation influenced the co-production. It is likely that there were pragmatic requirements to deliver public health interventions for funders, combined with potent power dynamics in communities, which could intensify the exclusion of already 'hard to reach' groups through, for example, task-based incentivisation for recruitment numbers.

2: Tailoring of intervention to context

All papers recognised to some degree the need to develop interventions what were tailored to the local context and employed a range of different methods to do so. These ranged from formal formative research processes that used literature reviewing, as well as qualitative (interviews, visual methods) and quantitative methods (surveys, analysis of routine or existing data sets, media audits) to understand the pre-existing social norms and behavioural patterns related to nurturing care (Kumar et al. 2018; Nguyen et al. 2019; Manjang et al. 2018), or to identify key messages/changes to focus on. In other studies, a pilot or series of pilots were used to refine the intervention, drawing on feedback from those delivering, receiving, and funding the interventions (e.g. Gautam et al. 2017).

Some interventions were built around existing community groups, such as 'Fathers Clubs' in rural Haiti, where the social groupings were already well established and health messages were incorporated into these in a way that was led by the fathers themselves, with support from health workers (Sloand & Gebrian, 2006), or the women's groups in rural Zambia (Kumar et al. 2018). In addition, existing infrastructure was often used, such as health facilities or community meeting spaces (Thuita et al. 2015; Mukaria et al. 2016).

For peer review only - http://bmjopen.bmj.com/site/about/guidelines.xhtml

292 Some papers acknowledged the importance of enabling or constraining environments, both physical (such as the location of handwashing stations or providing spaces for infant feeding) and not just focusing on health education messages. The importance of the social environment (such as the need to change perceptions about using soap in mass media campaigns) (Winter et al. 2021) was also identified. These approaches supplemented or complemented the arts components.

297 A significant limitation in the data was that authors did not always report on the opportunities for feedback from stakeholders, and so it is unclear whether those opportunities existed or not. Even where feedback opportunities are mentioned, it is not always clear what impact the feedback had on the subsequent delivery of the intervention, if any. Where performing arts were part of the intervention, there was very little reported in papers on how performing arts practitioners contributed to the overall design or content of the performance, so it is hard to judge the extent to which they influenced the artistic approaches taken.

304 Potentially relevant to the context was other similar or related public health or NGO programmes that were being undertaken in the region, and any impact they could have on outcomes, but these were rarely reported, possibly due to space limitations.

307 *3: Community participation in arts-based approaches*

308 Types of arts-based activities were wide-ranging and included: storytelling, story-acting, visual cues, interactive role-play, posters, comic books, nursery rhymes and songs, puppet shows, drama, drawing, singing competitions, animations, stories, tv promotions, games, dancing, fashion shows, comedy sketches, speeches, professional singing performances, street drama, skits (sketches), kitchen makeovers, decorating kitchens, letter exchanges, family drama, folk songs, cookery demonstrations, photo displays, Kalajarta (folk theatre), rupakas (musical dramas), writing scripts, community videos, TV spots with mini-dramas of intervention messages, radio messaging, brochure design, digital storytelling, drumming, testimonials, cartoon films, mass media campaigns, game

For peer review only - http://bmjopen.bmj.com/site/about/guidelines.xhtml

based and music based education, recitals, storybook reading, interactive games, and watching videos.

Less than half of the interventions delivered the arts components in a way that included specialist arts practitioners, such as local drama groups, traditional communicators, traditional theatre groups, songwriters, photographers, and other artists. Most interventions delivered the arts components through or with other members of the community, such as community health workers (normally with basic education), parents (mostly mothers) and wider community groups. In some cases, workers from NGOs were used to deliver intervention materials. It was much less common for papers to mention local or national government agents as involved in intervention delivery.

Very few papers commented on their theories or principles of how the arts were expected to support the intervention, with the exception of Olaide's paper (written by a Performing Arts scholar) which noted that it drew on Boal's concept of 'forum theatre' which is part of a broader concept of 'theatre of the oppressed', which is a widely used tool particularly in India, and eastern and southern Africa to facilitate social change (e.g. Brahma 2019). Most studies were reported by health scientists and in health journals so in very few cases was a detailed understanding of the process of arts production or performance interrogated in a critical or scholarly way. The intended mechanisms of change remained implicit, the arts were reported as a 'black box' conceptually and practically (no knowledge of the internal workings), and this was the biggest limitation identified in this review.

Other limitations to the reporting of the arts elements of the interventions included few detailed analyses of who was involved in the activities, how they were invited, and which parts of the communities may have been excluded for cultural, social, political, or economic reasons.

Finally, there was little consideration of how the arts activities aligned or not with broader community activities or norms, such as the provision of hospitality.

Were the interventions sustainable?

For peer review only - http://bmjopen.bmj.com/site/about/guidelines.xhtml

Approximately half of the studies did not record long-term outcomes which makes it difficult to judge their sustainability. There are of course issues with measuring effectiveness long-term, such as leakage of intervention into control groups (Kumar et al. 2018, Manaseki-Holland et al. 2021). Key to sustainability is whether change can be maintained after the withdrawal of the research team (Kim et al.).

A number of the studies claimed that the engagement of local arts meant that the studies would be more sustainable. The reasons given were that the arts interventions promoted willingness to engage (Akter et al. 2020) or included celebration as a motivation for continuing (Manaseki-Holland et al. 2021). Where interventions drew on local resources, this was often considered to make them more sustainable or low cost (Nguon et al. 2018, Manjang et al. 2018; Flax et al. 2014; Manaseki Holland et al. 2021, Olaide 2010; Makuria et al. 2016). Others argued that the use of arts would be better for promoting retention/memory of the intervention messages (Ghosh et al. 2006, Manaseki Holland et al. 2021) and that arts-based interventions help to create a positive attitude and contribute to people being more friendly to each other to promote sustainability (Olaide 2010). Positive motivational drivers particularly embedded in arts-based messaging was seen to be more effective than negative drivers such as issuing defecation fines (McMichael and Robinson 2016)

Other themes on claims for sustainability were that the diverse and intensive nature of activities over time promoted sustainability (McMichael and Robinson 2016), and that interventions were more realistic to implement when they were embedded in local infrastructure and resources so that people can access the intervention (Kumar et al. 2018, Limaye et al. 2018). It was, however, also noted that ensuring that those delivering the intervention are not distracted from their other duties when drawing on local infrastructure was also important, as well as ensuring appropriate additional incentivisation for involvement (Kumar et al. 2018; Winter et al. 2021). Local infrastructure issues, such as a lack of electricity or equipment, can make the delivery of materials such as videos challenging and affect sustainability (Winter et al. 2021). A final issue in sustainability was whether

For peer review only - http://bmjopen.bmj.com/site/about/guidelines.xhtml

other members of the family and community (beyond the mother) are brought in to engage with the intervention (Thuita et al. 2015) because broader family support was reported to bring longer-term sustainability of messaging.

To what extent were interventions transferable?

Systematic participatory approaches simplify the process of contextual adaptation (Akter et al. 2020) enabling the process of developing and delivering the intervention to be replicated (even if the content is different). Interventions that use existing staff, e.g. teachers or community health workers (Sloand and Gebrian 2006; Gautam et al. 2017), to deliver the intervention find that this promotes transferability into contexts where the health or education systems (Flax et al. 2014) or infrastructure (Kumar et al. 2018; Limaye et al. 2018; Mukuria et al. 2016) are similar and these professionals or paraprofessionals receive broadly similar training. However, there is always a risk that staff groups in different contexts may not buy into the intervention. In addition, varying relationships between governments and NGOs can be a limitation to transferability in some contexts (Kim et al. 2018).

To what extent were interventions scalable?

Small-scale interventions can limit the potential for scalability (McMichael and Robinson 2016) particularly where they are reliant on individual motivation and momentum. Broad campaigns that capture large audiences quickly are more likely to be scalable, but the specificity of messaging can be challenging (Sanghvi et al. 2013), which may limit the effectiveness or traction of the messages. Elements of group interaction also maximise the efficient use of resources (Akter et al. 2020), making the intervention more scalable, as well as having the potential to enhance community ties. A further point raised on scalability of arts-based interventions was when tried and tested health intervention materials were used alongside arts-based approaches leading to improved buy in for scaling (Callery et al. 2020).

For peer review only

For peer review only - http://bmjopen.bmj.com/site/about/guidelines.xhtml

Major limitations around scalability within LMICs are cost and resource, with many of these interventions seeking to minimize the resource burden on government or communities (Manjang et al. 2018; Sloand and Gebrian 2006; Flax et al. 2014; Ghosh et al. 2006; Kim et al. 2018; Olaide 2010; Mukuria et al. 2016; Thuita et al. 2015). Examples of this are using local storytelling (Goodman & Dent 2019) or theatre (Callery et al. 2020, Ghosh et al. 2006), which are cheaper than the production costs of video making (Winter et al. 2021). Some costs can be prohibitive for scaling e.g. TV ads (Kim et al. 2018) and cameras on smartphones (Limaye et al. 2018). For this reason, NGO support and finances can be critical requirements for scalability (Sloand and Gebrian 2006; Flax et al. 2014; Ghosh et al. 2006; Nguyen et al. 2019; Sanghvi et al. 2016).

6. Discussion

Contribution (empirical)

Our findings on community-based arts interventions targeted at improving health and development outcomes for 0-5 year olds in Low and Middle Income Countries (LMICs) have revealed a number of gaps in current knowledge and understanding of what works for whom in which contexts.

The literature on interventions currently lacks input and leadership from academics working in the arts, humanities, and the social sciences. The lack of inclusion of arts scholars means there is a lack of theory presented on how the arts were expected to support the interventions delivered, and it is unclear what was actually done in practice with local artists. Arts scholarship does engage with some of these issues (e.g. Baxter & Low, 2016) but not often in an interdisciplinary way where it is linked to outcomes of interventions (hence not meeting our inclusion criteria). The lack of social science input likely leads to a dominant focus of the literature on outcomes as opposed to the processes used to achieve the outcomes. While there are literatures that suggest that the arts have the potential to break down power dynamics and provide communities a sense of ownership of the intervention with audiences owning the art or the performance (Kuppers, 2007; Kay, 2000; Lowe

For peer review only - http://bmjopen.bmj.com/site/about/guidelines.xhtml

2000), these critical approaches were not used to interpret the findings for these interventions. This means that opportunities to learn about what works for whom and in which contexts are often missed.

The lack of local lead authors of studies and insufficient studies from some regions including West and Southern Africa and South America means that contextual understanding is often lacking in the literature reviewed and this gap is particularly big for the regions with no studies, although we recognise that there might be relevant publications in different languages in these regions.

The majority of the studies included in this review used a model of co-production that involved bringing in communities to adapt existing interventions at a later stage of the development process as opposed to including communities from the conceptualisation stage. The minority of studies that did work with communities throughout the process to co-produce were able to document benefits including time to build trust, community capacity, empowering local leadership, and developing a true collaboration. These factors were all relevant for intervention sustainability because they may empower the community to take the intervention forward once the intervention team leave or funding ends. Overall, these elements were poorly reported, although this may reflect the focus and limited words counts in target journals.

Studies that entered communities early to co-design were better placed to draw on existing health systems including community health workers, social infrastructures such as fathers' or mothers' groups, and community facilities such as community spaces. Using existing infrastructures saves investment in time and resources to build and grow social systems and staffing infrastructures to support these types of intervention. This facilitates the sustainability of the intervention. Studies that did use existing structures reported the importance of the level of adequate functioning of these systems to positively influence intervention outcomes. There was also a commonly reported need to consider ongoing training of workers such as community health volunteers to maintain the success of intervention messaging and to think about how such training becomes sustainable

For peer review only - http://bmjopen.bmj.com/site/about/guidelines.xhtml

beyond the study. This review also identified studies that did not report interaction with existing community structures such as health systems which was a missed opportunity for joined up thinking and promoting sustainability of the intervention beyond the project end point.

Contribution (theoretical)

The interrelatedness and interdependence of the three themes (co-production, local adaptation, and arts-based delivery) underpins the theoretical contribution of this review. While co-production and local adaptation offer two elements of 'scaffolding' that are necessary but not always sufficient for change, our findings suggest that the arts-based delivery approach can act as a bridge for overcoming some of the contextual and political challenges of delivering nurturing care interventions in LMICs. The use of realist methods for synthesizing the literature allowed us to explore multiple and intersecting mechanisms of action, and to explore those mechanisms within different contexts. It allowed us to see that the success of the arts elements is dependent on effective collaboration and adapting to context, and to see that the arts can enhance and enact the benefits of collaborative working and careful local adaptation processes. Nevertheless the lack of explicit reporting in papers on the mechanisms through which the arts work to enact change means that we are left with limited knowledge about what works for whom in what context. For instance we have learned little about audience involvement in the arts, cultural embeddedness of the arts, how the arts works most effectively with the public health services, how the arts messages can be effectively shaped by those who know the health elements, the role of knowledge and background in the health topic of the artists, inclusion or inclusion of certain population groups through the arts, the role of the arts venues, and how arts interact with social norms. All of these would be important to gain understanding of to better understand the true role of the arts in facilitating health interventions to improve nurturing care in infants and young children. Interdisciplinary investigations integrating insights from arts scholarship and from the public health fields could support future research on this.

For peer review only - http://bmjopen.bmj.com/site/about/guidelines.xhtml

There is evidence to suggest the importance of working toward synergy between partners (Bamuturaki, 2016) in both goals and processes (Kwan et al., 2022), and of including local people, community leaders, public health and education professionals, policy-makers, and researchers, as well as funders of nurturing care interventions, in order to achieve more than each could alone (Coombe et al., 2020). Our findings supported this through stressing the value of regular formal (Akter et al. 2020) or informal feedback opportunities (e.g. Olaide 2010) and the longest running intervention which had regular participatory meetings (Sloand & Gebriand 2006). This builds trust (Jones and Barry, 2011; Jagosh et al., 2015) and transparency (Moore de Peralta et al., 2020; Charles et al., 2021).

When collaborations also acknowledge that interventions must be tailored to context in both the design and implementation phases, more can be achieved. Complementing or integrating with existing educational and health infrastructures and human resources was identified as being key, as was an understanding of cultural and social norms (McMichael & Robinson, 2016; Winter et al., 2021, and Nguon et al., 2018) particularly where interventions were seeking to make sustainable change.

However, a key risk to effective co-production and tailoring to local context is the dysregulating effect of power imbalances, so enabling empowerment of those with less historical or cultural power in collaborations is a vital element of success (George et al., 2015). The review has highlighted how challenging it is for collaborations to achieve high levels of balance and equity in contributions, and how rare it is for these issues to be explicitly or critically reported in research papers. Our analysis can make an additional theoretical contribution here by identifying the potential for arts-based approaches to act as a bridge for some of these challenges of power imbalances. There are two sides to this - the first more practical and the second more political.

First, *practically*, our findings suggest that engagement with community arts increases the potential effectiveness of the intervention in achieving (shared) goals of improved health and child

For peer review only

For peer review only - http://bmjopen.bmj.com/site/about/guidelines.xhtml

development. Both the arts and community aspects support engagement in a shared experience of performance and through the congruence of local arts with local culture. Our findings suggest that there are two elements to this: increasing motivation to engage and shifting long standing social norms. Positive motivation is engendered though things like a festival-like atmosphere, diversity of art forms, and the focus on positive impacts of change. The social dimensions of motivation may be particularly encouraged by communal experiences which contribute to group binding (Stewart et al., 2022; Faigan and Stein, 2014). The use of local arts, local performers and organisers (Callery et al. 2020; Ghosh et al. 2006) and even local stories (Nguon et al., 2018) increases the sense of ownership (Olaide 2010). Interventions that are able to acknowledge and then shift long-established social norms from within are much more likely to be sustainable, rather than introducing external ideas. While the studies in the review rarely explicitly commented on social norms, our analysis was able to identify possible elements of the individual, social, material and institutional domains (Cislaghi and Heise, 2019) in which social norms operate. The active engagement and the shared memories of an event or experience were considered to promote discussion of intervention materials, improve its retention individually and, importantly, its diffusion through social networks, reaching beyond intervention participants (Ghosh et al. 2006; Manaseki-Holland et al. 2021).

Second, *politically,* our findings suggest that these sorts of community arts-based delivery approaches, when combined with co-production and local adaptation, have the potential to disrupt some of the neo-colonial tendencies in global health research and practice. This involves shifting the balance of power away from Western biomedical models, towards communities and their historical and cultural modes of artistic expression and cultural re/production. Culturally embedded arts as opposed to non-culturally embedded arts have the most potential to achieve this outcome. Even the frequent delivery of these interventions in local languages suggests they are at least partially outwith the surveillance of global health researchers. While in some cases attempts to address power imbalances were evident, such as minority outreach and platforming during performances (Callery et al 2020); the targeting of women’s empowerment through agricultural work and education (Kumar

For peer review only - http://bmjopen.bmj.com/site/about/guidelines.xhtml

515 et al. 2018); targeting father and grandmother involvement in nurturing care practices, (Thuita et al.
516 2015; Mukuria et al. 2016), or setting up partnership agreements (Gautam et al. 2017), in many
517 cases this was not addressed explicitly in the papers.

518 *Strengths and limitations of the review*

519 The review only captures the published peer-reviewed journal articles that are available in the
520 restricted language criteria of the review. This likely results in a positive results publication bias, a
521 lack of reports on arts-based interventions found in the grey literature including NGO reports, and a
522 the lack of French and Spanish literatures, may result in fewer interventions from countries in West
523 Africa or South America. Despite searching a wide range of databases, we have identified that the
524 papers that have been included are focussed on health sciences rather than the arts/social science
525 parts of the research. Many arts scholars publish in monographs or edited collections which are not
526 always effectively captured through database searches.

527 *Recommendations for further research*

528 There is a significant gap in the international literature, for studies that are led by researchers from
529 LMICs and/or by those in the arts, humanities and social sciences. Bringing these voices into the
530 research would enable better integration of the theoretical underpinnings regarding the potential
531 success of arts-based intervention processes in nurturing care interventions. It would also facilitate
532 better understanding of intervention processes and cultural context of these. Specific steps could be
533 considered to ensure this can happen, such as leadership from LMICs, devolving funding decisions,
534 and supporting and funding projects led from the arts, humanities and social sciences rather than
535 the health and clinical sciences, although it is worth noting that the funding available for health
536 research in much higher than the arts and humanities. Methods for ongoing reflective evaluation of
537 the quality and equity in collaborations would be useful. Contextual issues may be relevant,
538 including sources of funding being weighted toward health funders and the motivation for
539 academics to publish in high impact journals, which tend to be more STEMM-focused. Finaly,

For peer review only - http://bmjopen.bmj.com/site/about/guidelines.xhtml

reporting in journals of interventions with arts should include better documentation of their learning regarding the process of intervention delivery and the power dynamics involved in this.

Recommendations for policy and practice

Nurturing care interventions are complex involving the health system, early education providers, social workers, families, extended families and community leadership structures. As we have shown in this review there are attempts taking place to incorporate the practical and political power of the arts into intervention design to empower communities to own nurturing care interventions, to facilitate message sharing and to embed intervention messaging into the culture of communities. Our findings suggest that these types of intervention have the potential for greater success in being contextually relevant when they include local knowledge including artists, are evaluated by teams that include those with experience in the arts, include collaborative work between artists and public health experts to adapt and deliver the intervention, and where local researchers take a lead or active role in the intervention delivery, evaluation and reporting.

There is also evidence in this review that where arts-based interventions embed into existing infrastructures (e.g. health services) and there is a clear plan for ongoing resource for training of those delivering the intervention that sustainability is likely. To better understand what works for whom in which contexts when designing nurturing care arts-based interventions there is a need for studies to better document their learning regarding the process of intervention delivery and the power dynamics involved in this. Interventions that have included these elements have been able to show positive effects for nurturing care in low and middle income countries.

For peer review only

References

Akter, F., Rahman, M., Pitchik, H.O., Winch, P.J., Fernald, L.C., Nurul Huda, T.M., Jahir, T., Amin, R., Das, J.B., Hossain, K. and Shoab, A.K. (2020). Adaptation and integration of psychosocial stimulation, maternal mental health and nutritional interventions for pregnant and lactating women in rural Bangladesh. *International journal of environmental research and public health*, *17*(17), p.6233.

Al-Delaimy, A.K., Al-Mekhlafi, H.M., Lim, Y.A., Nasr, N.A., Sady, H., Atroosh, W.M. and Mahmud, R., (2014). Developing and evaluating health education learning package (HELP) to control soil-transmitted helminth infections among Orang Asli children in Malaysia. *Parasites & vectors*, *7*(1), pp.1-18.

Baxter, V. and Low, K.E. (eds.), 2017. *Applied theatre: Performing health and wellbeing*. Bloomsbury Publishing.

Bechtoldt, M. N., De Dreu, C. K. W., Nijstad, B. A., & Choi, H.-S. (2010). Motivated information processing, social tuning, and group creativity. *Journal of Personality and Social Psychology*, *99*(4), 622–637. https://doi.org/10.1037/a0019386

Brahma, J., Pavarala, V. and Belavadi, V. (2019) Driving social change through forum theatre: A study of Jana Sanskriti in West Bengal, India. *Asia Pacific Media Educator*, *29*(2), pp.164-177.

Britto, P. R., Lye, S. J., Proulx, K., Yousafzai, A. K., Matthews, S. G., Vaivada, T., Perez-Escamilla, R., Rao, N., Ip, P., Fernald, L. C. H., MacMillan, H., Hanson, M., Wachs, T. D., Yao, H., Yoshikawa, H., Cerezo, A., Leckman, J. F., & Bhutta, Z. A. (2017). Nurturing care: Promoting early childhood development. *The Lancet*, *389*(10064), 91–102. https://doi.org/10.1016/S0140-6736(16)31390-3

Callery, J.J., Sanann, N., Tripura, R., Buntau, T., Peto, T.J., Kunthea, P., Pell, C., Nguon, C., Lek, D. and Cheah, P.Y., (2021). Engaging ethnic minority communities through performance and arts: health education in Cambodian forest villages. *International Health*, *13*(2), pp.188-195.

Charles, J. (2021). How Partnership Trust can Facilitate and Result from CBPR: An Assessment of Situational, Organizational, and Institutional Related Factors. *Epidemiology International Journal*, *5*(2). https://doi.org/10.23880/EIJ-16000186

For peer review only

Cislaghi, B., & Heise, L. (2019). Using social norms theory for health promotion in low-income countries. *Health Promotion International*, *34*(3), 616–623. https://doi.org/10.1093/heapro/day017

Coombe, C. M., Chandanabhumma, P. P., Bhardwaj, P., Brush, B. L., Greene-Moton, E., Jensen, M., Lachance, L., Lee, S. Y. D., Meisenheimer, M., Minkler, M., Muhammad, M., Reyes, A. G., Rowe, Z., Wilson-Powers, E., & Israel, B. A. (2020). A Participatory, Mixed Methods Approach to Define and Measure Partnership Synergy in Long-standing Equity-focused CBPR Partnerships. *American Journal of Community Psychology*, 10.1002/ajcp.12447. https://doi.org/10.1002/ajcp.12447

de Peralta, A. M., Smithwick, J., Torres, M. E., & de Peralta, M. (2020). Perceptions and determinants of partnership trust in the context of Community-Based Participatory Research. *Participatory Research*, *13*(1).

Callery, J.J., Sanann, N., Tripura, R., Buntau, T., Peto, T.J., Kunthea, P., Pell, C., Nguon, C., Lek, D. and Cheah, P.Y., (2021). Engaging ethnic minority communities through performance and arts: health education in Cambodian forest villages. *International Health*, *13*(2), pp.188-195.

Durose, C., Perry, B., & Richardson, L. (2022). Is co-production a 'good' concept? Three responses. *Futures*, *142*, 102999. https://doi.org/10.1016/j.futures.2022.102999

Egid, B. R., Roura, M., Aktar, B., Quach, J. A., Chumo, I., Dias, S., Hegel, G., Jones, L., Karuga, R., Lar, L., López, Y., Pandya, A., Norton, T. C., Sheikhattari, P., Tancred, T., Wallerstein, N., Zimmerman, E., & Ozano, K. (2021). 'You want to deal with power while riding on power': Global perspectives on power in participatory health research and co-production approaches. *BMJ Global Health*, *6*(11), e006978. https://doi.org/10.1136/bmjgh-2021-006978

Faigin, D., & Stein, C. (2014). Community-Based Theater and Adults with Psychiatric Disabilities: Social Activism, Performance and Community Engagement. *American Journal of Community Psychology*, *55*. https://doi.org/10.1007/s10464-014-9695-6

For peer review only

For peer review only - http://bmjopen.bmj.com/site/about/guidelines.xhtml

Flax, V.L., Negerie, M., Ibrahim, A.U., Leatherman, S., Daza, E.J. and Bentley, M.E. (2014). Integrating group counseling, cell phone messaging, and participant-generated songs and dramas into a microcredit program increases Nigerian women's adherence to international breastfeeding recommendations. *The Journal of nutrition*, *144*(7), pp.1120-1124.

Gallant, K., Litwiller, F., Hamilton-Hinch, B., Hutchinson, S., Rehman, L., & Singleton, J. (2017). Community-Based Experiential Education: Making It Meaningful to Students Means Making It Meaningful for Everyone. *SCHOLE: A Journal of Leisure Studies and Recreation Education*, *32*(2), 146–157. https://doi.org/10.18666/SCHOLE-2017-V32-I2-3

Gautam, O.P., Schmidt, W.P., Cairncross, S., Cavill, S. and Curtis, V. (2017). Trial of a novel intervention to improve multiple food hygiene behaviors in Nepal. *The American journal of tropical medicine and hygiene*, *96*(6), p.1415.

Gautam, O.P. and Curtis, V. (2021). Food hygiene practices of rural women and microbial risk for children: Formative research in Nepal. *The American Journal of Tropical Medicine and Hygiene*, *105*(5), p.1383.

George, A. S., Mehra, V., Scott, K., & Sriram, V. (2015). Community Participation in Health Systems Research: A Systematic Review Assessing the State of Research, the Nature of Interventions Involved and the Features of Engagement with Communities. *PLOS ONE*, *10*(10), e0141091. https://doi.org/10.1371/journal.pone.0141091

Ghosh, S.K., Patil, R.R., Tiwari, S. and Dash, A.P. (2006). A community-based health education programme for bio-environmental control of malaria through folk theatre (Kalajatha) in rural India. *Malaria journal*, *5*, pp.1-7.

Goncalo, J. A., & Duguid, M. M. (2012). Follow the crowd in a new direction: When conformity pressure facilitates group creativity (and when it does not). *Organizational Behavior and Human Decision Processes*, *118*(1), 14–23. https://doi.org/10.1016/j.obhdp.2011.12.004

For peer review only

For peer review only - http://bmjopen.bmj.com/site/about/guidelines.xhtml

Goodman, G. and Dent, V.F. (2019). A story grows in rural Uganda: Studying the effectiveness of the storytelling/story-acting (STSA) play intervention on Ugandan preschoolers' school readiness skills. *Journal of Infant, Child, and Adolescent Psychotherapy*, *18*(3), pp.288-306.

Greenhalgh, T., Jackson, C., Shaw, S., & Janamian, T. (2016). Achieving Research Impact Through Co-creation in Community-Based Health Services: Literature Review and Case Study. *The Milbank Quarterly*, *94*(2), 392–429. https://doi.org/10.1111/1468-0009.12197

Jagosh, J., Bush, P. L., Salsberg, J., Macaulay, A. C., Greenhalgh, T., Wong, G., Cargo, M., Green, L. W., Herbert, C. P., & Pluye, P. (2015a). A realist evaluation of community-based participatory research: Partnership synergy, trust building and related ripple effects. *BMC Public Health*, *15*(1), 725. https://doi.org/10.1186/s12889-015-1949-1

Jagosh, J., Bush, P. L., Salsberg, J., Macaulay, A. C., Greenhalgh, T., Wong, G., Cargo, M., Green, L. W., Herbert, C. P., & Pluye, P. (2015b). A realist evaluation of community-based participatory research: Partnership synergy, trust building and related ripple effects. *BMC Public Health*, *15*(1), 725. https://doi.org/10.1186/s12889-015-1949-1

Jagosh, J., Pluye, P., Wong, G., Cargo, M., Salsberg, J., Bush, P. L., Herbert, C. P., Green, L. W., Greenhalgh, T., & Macaulay, A. C. (2014). Critical reflections on realist review: Insights from customizing the methodology to the needs of participatory research assessment. *Research Synthesis Methods*, *5*(2), 131–141. https://doi.org/10.1002/jrsm.1099

Jones, J., & Barry, M. M. (2011). Exploring the relationship between synergy and partnership functioning factors in health promotion partnerships. *Health Promotion International*, *26*(4), 408–420. https://doi.org/10.1093/heapro/dar002

Kay, A. (2000). Art and community development: The role the arts have in regenerating communities. *Community Development Journal*, *35*(4), 414–424. https://doi.org/10.1093/cdj/35.4.414

For peer review only

For peer review only - http://bmjopen.bmj.com/site/about/guidelines.xhtml

Keneth, Bamuturaki. (2016). Participatory Research in Theatre for Development: An Evaluative Paradigm of the Walukuba Project in Eastern Uganda. *Consciousness, Literature and the Arts*, *17*(3). http://www.dmd27.org/bamuturaki.html

Kim, S.S., Nguyen, P.H., Tran, L.M., Sanghvi, T., Mahmud, Z., Haque, M.R., Afsana, K., Frongillo, E.A., Ruel, M.T. and Menon, P. (2018). Large-scale social and behavior change communication interventions have sustained impacts on infant and young child feeding knowledge and practices: results of a 2-year follow-up study in Bangladesh. *The Journal of nutrition*, *148*(10), pp.1605-1614.

Kohrt, B. A., Asher, L., Bhardwaj, A., Fazel, M., Jordans, M. J. D., Mutamba, B. B., Nadkarni, A., Pedersen, G. A., Singla, D. R., & Patel, V. (2018). The Role of Communities in Mental Health Care in Low- and Middle-Income Countries: A Meta-Review of Components and Competencies. *International Journal of Environmental Research and Public Health*, *15*(6), Article 6. https://doi.org/10.3390/ijerph15061279

Kumar, N., Nguyen, P.H., Harris, J., Harvey, D., Rawat, R. and Ruel, M.T. (2018). What it takes: evidence from a nutrition-and gender-sensitive agriculture intervention in rural Zambia. *Journal of Development Effectiveness*, *10*(3), pp.341-372.

Kuppers, P. (2007). Community arts practices: Improvising being-together. In *The Community Performance Reader*. Routledge.

Kwan, B. M., Brownson, R. C., Glasgow, R. E., Morrato, E. H., & Luke, D. A. (2022). Designing for Dissemination and Sustainability to Promote Equitable Impacts on Health. *Annual Review of Public Health*, *43*(1), 331–353. https://doi.org/10.1146/annurev-publhealth-052220-112457

Limaye, N.P., Rivas-Nieto, A.C., Carcamo, C.P. and Blas, M.M. (2018). Nuestras Historias-Designing a novel digital story intervention through participatory methods to improve maternal and child health in the Peruvian Amazon. *PLoS One*, *13*(11), p.e0205673.

For peer review only

For peer review only - http://bmjopen.bmj.com/site/about/guidelines.xhtml

Logie, C. H., Dias, L. V., Jenkinson, J., Newman, P. A., MacKenzie, R. K., Mothopeng, T., Madau, V., Ranotsi, A., Nhlengethwa, W., & Baral, S. D. (2019). Exploring the Potential of Participatory Theatre to Reduce Stigma and Promote Health Equity for Lesbian, Gay, Bisexual, and Transgender (LGBT) People in Swaziland and Lesotho. *Health Education & Behavior*, *46*(1), 146–156. https://doi.org/10.1177/1090198118760682

Lowe, S. (2000). Creating Community: Art for Community Development. *Journal of Contemporary Ethnography*, *29*(3), 357–386. https://doi.org/10.1177/089124100129023945

Lu, C., Cuartas, J., Fink, G., McCoy, D., Liu, K., Li, Z., Daelmans, B., & Richter, L. (2020). Inequalities in early childhood care and development in low/middle-income countries: 2010–2018. *BMJ Global Health*, *5*(2), e002314. https://doi.org/10.1136/bmjgh-2020-002314

Manjang, B., Hemming, K., Bradley, C., Ensink, J., Martin, J.T., Sowe, J., Jarju, A., Cairncross, S. and Manaseki-Holland, S. (2018). Promoting hygienic weaning food handling practices through a community-based programme: intervention implementation and baseline characteristics for a cluster randomised controlled trial in rural Gambia. *BMJ open*, *8*(8), p.e017573.

Manaseki-Holland, S., Manjang, B., Hemming, K., Martin, J.T., Bradley, C., Jackson, L., Taal, M., Gautam, O.P., Crowe, F., Sanneh, B. and Ensink, J. (2021). Effects on childhood infections of promoting safe and hygienic complementary-food handling practices through a community-based programme: A cluster randomised controlled trial in a rural area of The Gambia. *PLoS medicine*, *18*(1), p.e1003260.

McMichael, C. and Robinson, P. (2016). Drivers of sustained hygiene behaviour change: a case study from mid-western Nepal. *Social science & medicine*, *163*, pp.28-36.

Mukuria, A.G., Martin, S.L., Egondi, T., Bingham, A. and Thuita, F.M. (2016). Role of social support in improving infant feeding practices in Western Kenya: a quasi-experimental study. *Global Health: Science and Practice*, *4*(1), pp.55-72.

For peer review only - http://bmjopen.bmj.com/site/about/guidelines.xhtml

Nguon, C., Dysoley, L., Davoeung, C., Sovann, Y., Sanann, N., Sareth, M., Kunthea, P., Vuth, S., Sovann, K., Kol, K. and Heng, C. (2018). Art and theatre for health in rural Cambodia. *Global Bioethics*, *29*(1), pp.16-21.

Nguyen, P.H., Frongillo, E.A., Kim, S.S., Zongrone, A.A., Jilani, A., Tran, L.M., Sanghvi, T. and Menon, P. (2019). Information diffusion and social norms are associated with infant and young child feeding practices in Bangladesh. *The Journal of nutrition*, *149*(11), pp.2034-2045.

Olaide, N.T., (2010) Maternal mortality and theatre intervention: A case study of Oluyole local government in Ibadan of Nigeria. US-China Foreign Language, ISSN 1539-8080, USA, November 2010, Volume 8, No.11 (Serial No.86).

Pawson, R., Greenhalgh, T., Harvey, G., & Walshe, K. (2005). Realist review—A new method of systematic review designed for complex policy interventions. *Journal of Health Services Research & Policy*, *10*(1_suppl), 21–34. https://doi.org/10.1258/1355819054308530

Putland, C. (2008). Lost in Translation: The Question of Evidence Linking Community-based Arts and Health Promotion. *Journal of Health Psychology*, *13*(2), 265–276. https://doi.org/10.1177/1359105307086706

Richter, L. M., Daelmans, B., Lombardi, J., Heymann, J., Boo, F. L., Behrman, J. R., Lu, C., Lucas, J. E., Perez-Escamilla, R., Dua, T., Bhutta, Z. A., Stenberg, K., Gertler, P., & Darmstadt, G. L. (2017). Investing in the foundation of sustainable development: Pathways to scale up for early childhood development. *Lancet (London, England)*, *389*(10064), 103–118. https://doi.org/10.1016/S0140-6736(16)31698-1

Rossiter, K., Kontos, P., Colantonio, A., Gilbert, J., Gray, J., & Keightley, M. (2008). Staging data: Theatre as a tool for analysis and knowledge transfer in health research. *Social Science & Medicine*, *66*(1), 130–146. https://doi.org/10.1016/j.socscimed.2007.07.021

For peer review only

For peer review only - http://bmjopen.bmj.com/site/about/guidelines.xhtml

Sanghvi, T., Jimerson, A., Hajeebhoy, N., Zewale, M. and Nguyen, G.H., (2013). Tailoring communication strategies to improve infant and young child feeding practices in different country settings. *Food and nutrition bulletin*, *34*(3_suppl2), pp.S169-S180.

Sanghvi, T., Haque, R., Roy, S., Afsana, K., Seidel, R., Islam, S., Jimerson, A. and Baker, J. (2016). Achieving behaviour change at scale: Alive & Thrive's infant and young child feeding programme in Bangladesh. Maternal & child nutrition, 12, pp.141-154.

Schulz, A. J., Israel, B. A., & Lantz, P. (2003). Instrument for evaluating dimensions of group dynamics within community-based participatory research partnerships. *Evaluation and Program Planning*, *26*(3), 249–262. https://doi.org/10.1016/S0149-7189(03)00029-6

Sloand, E. and Gebrian, B. (2006). Fathers clubs to improve child health in rural Haiti. *Public Health Nursing*, *23*(1), pp.46-51.

Stein, C. H., & Faigin, D. A. (2015). Community-Based Arts Initiatives: Exploring the Science of the Arts. *American Journal of Community Psychology*, *55*(1), 70–73. https://doi.org/10.1007/s10464-014-9698-3

Stewart, L., McConnell, B. B., Darboe, B., Glover, V., Huma, H. B., Sanfilippo, K. R. M., Cross, I., Ceesay, H., Ramchandani, P., & Cornelius, V. (2022). Social singing, culture and health: Interdisciplinary insights from the CHIME project for perinatal mental health in The Gambia. *Health Promotion International*, *37*(Supplement_1), i18–i25. https://doi.org/10.1093/heapro/daab210

Thuita, F.M., Martin, S.L., Ndegwa, K., Bingham, A. and Mukuria, A.G. (2015). Engaging fathers and grandmothers to improve maternal and child dietary practices: planning a community-based study in western Kenya. *African Journal of Food, Agriculture, Nutrition and Development*, *15*(5), pp.10386-10405.

Torrissen, W., & Stickley, T. (2018). Participatory theatre and mental health recovery: A narrative inquiry. *Perspectives in Public Health*, *138*(1), 47–54. https://doi.org/10.1177/1757913917723944

For peer review only

For peer review only - http://bmjopen.bmj.com/site/about/guidelines.xhtml

UN. (2018). *Sustainable Development Goals*. https://www.who.int/europe/about-us/our-work/sustainable-development-goals

UNICEF. (2021). *Levels and trends in child mortality*. United Nations Inter-agency Group for Child Mortality Estimation. https://data.unicef.org/resources/levels-and-trends-in-child-mortality/

Winter, J.C., Darmstadt, G.L., Lee, S.J. and Davis, J. (2021). The potential of school-based WASH programming to support children as agents of change in rural Zambian households. *BMC Public Health*, *21*, pp.1-13.

Wong, G., Greenhalgh, T., Westhorp, G. *et al.* (2013) RAMESES publication standards: realist syntheses. *BMC Med* **11**, 21. https://doi.org/10.1186/1741-7015-11-21

Yassi, A., Spiegel, J. B., Lockhart, K., Fels, L., Boydell, K., & Marcuse, J. (2016). Ethics in Community-University-Artist Partnered Research: Tensions, Contradictions and Gaps Identified in an ‘Arts for Social Change’ Project. *Journal of Academic Ethics*, *14*(3), 199–220. https://doi.org/10.1007/s10805-016-9257-7

For peer review only

For peer review only - http://bmjopen.bmj.com/site/about/guidelines.xhtml

Table 1: Search Terms

Outcomes	Arts	Community	LMIC
(‘Child health’ OR ‘infant health’ OR ‘baby health’ OR ‘early years health’ OR ‘nurturing care’ OR ‘child care’ OR ‘child feeding’ OR ‘breastfeeding’ OR ‘hygiene’ OR ‘diarrhoea’ OR ‘sanitation’ OR ‘maternal health’ OR ‘maternal care’ OR ‘paternal health’ OR ‘paternal care’ OR ‘child development’ OR ‘child education’ OR ‘child wellbeing’ OR ‘maternal wellbeing’ OR ‘maternal mental health’ OR ‘child mental health’ OR ‘social cohesion’ OR ‘community cohesion’ OR ‘trust’ OR ‘community support’ OR ‘community development’ OR ‘social norms’ OR ‘health practices’ OR ‘care practices’) AND	(‘Arts’ OR ‘drama’ OR ‘theatre’ OR ‘theater’ or ‘performative’ OR ‘creative’ OR ‘ music’ OR ‘story’ AND NOT 'antiretroviral') AND	(‘Arts’ OR ‘drama’ OR ‘theatre’ OR ‘theater’ or ‘performative’ OR ‘creative’ OR ‘ music’ OR ‘story’ AND NOT 'antiretroviral') AND	(‘Low-income country’ OR ‘middle-income country’ OR ‘low income country’ OR ‘middle income country’ OR ‘LMIC’ OR ‘developing country’ OR ‘global south’ OR Afghanistan OR ‘Albania’ OR ‘Algeria’ OR ‘Angola’ OR ‘Antigua’ …. OR ‘Yemen’ OR ‘Zambia’ OR ‘Zimbabwe’)

For peer review only

Table 2: Relevance Criteria for Inclusion

**Initial criteria**	
**Include**	**Exclude**
Intervention is co-produced (can be led by community, researchers or public health, or equally balanced)<br><br>Focuses on early years water/food sanitation (0-5) or child development<br><br>Intervention takes place in LMIC<br><br>Intervention is arts-based<br><br>Any study that:<br>- Reports the design and delivery of an intervention<br>- Reports an intervention evaluation, using a randomized control trial (RCT) design or in other evaluation methods (quantitative or qualitative)<br>- Theoretical or critical articulation of an intervention	Opinions, letters or editorials<br><br>Conducted over 20 years ago<br><br>Any language other than English.
Clarifications added after discussions about relevance of articles	
Intervention is arts-based, aimed toward public health and/or changes in health behaviours	Health or nurturing care not a primary outcome, but as an unintended (if positive) outcome.<br><br>Maternal health only as outcome<br><br>Outcome is 'Community health' but without focus or measure(s) specific to under 5s

For peer review only

	Theory only, rather than reporting related to a specific intervention<br>Arts/story-based methodological evaluation only<br>Arts-based intervention is individual therapy, rather than related to public health outcomes

For peer review only

Author (Date)	Inter-vention Date	Title (s)	Country of interventio n	Country of first author	Primary Discipline
Akter et al. (2020)	2017	Adaptation and Integration of Psychosocial Stimulation, Maternal Mental Health and Nutritional Interventions for Pregnant and Lactating Women in Rural Bangladesh	Bangladesh	Bangladesh	Public Health (Infectious Disease)
Al-Delaimy et al. (2014)	2012-2013	Developing and evaluating health education learning package (HELP) to control soil-transmitted helminth infections among Orang Asli children in Malaysia	Malaysia	Iraq	Public health (Health Education & Community Medicine)
Callery et al. (2020)	2018	Engaging ethnic minority communities through performance and arts: health education in Cambodian forest villages	Cambodia	Thailand	Tropical Medicine
Flax et al. (2014)	2011-2012	Integrating Group Counseling, Cell Phone Messaging, and Participant-Generated Songs and Dramas into a Microcredit Program Increases Nigerian Women's Adherence to International Breastfeeding Recommendations	Nigeria	US	Public Health Nutrition
Goodman and Dent (2019)	2014	A Story Grows in Rural Uganda: Studying the Effectiveness of the Storytelling/Story-Acting (STSA) Play Intervention on Ugandan Preschoolers' School Readiness Skills	Uganda	US	Psychotherapy
Gautam et al. (2017) Gautam and Curtis (2021)	2012-2013	Trial of a Novel Intervention to Improve Multiple Food Hygiene Behaviors in Nepal<br>Food Hygiene Practices of Rural Women and Microbial Risk for Children: Formative Research in Nepal	Nepal	UK	Tropical Medicine
Ghosh et al. (2006)	2001	A community-based health education programme for bio-environmental control of malaria through folk theatre (Kalajatha) in rural India	India	India	Epidemiology (Malaria research)

For peer review only

Kim et al. (2018)	2010-2014	Large-Scale Social and Behavior Change Communication Interventions Have Sustained Impacts on Infant and Young Child Feeding Knowledge and Practices: Results of a 2-Year Follow-Up Study in Bangladesh	Bangladesh	US	Nutrition
Kumar et al. (2018)	2011-2015	What it takes: evidence from a nutrition- and gender-sensitive agriculture intervention in rural Zambia	Zambia	USA	Food policy
Limaye et al. (2018)	2017	Nuestras Historias- Designing a novel digital story intervention through participatory methods to improve maternal and child health in the Peruvian Amazon	Peru	USA	Pediatrics
Manjang, et al. (2018) Manaseki-Holland et al. (2021)	2014-2015	Promoting hygienic weaning food handling practices through a community-based programme: intervention implementation and baseline characteristics for a cluster randomised controlled trial in rural Gambia. Effects on childhood infections of promoting safe and hygienic complementary-food handling practices through a community-based programme: A cluster randomised controlled trial in a rural area of The Gambia	The Gambia	The Gambia/ UK	Public Health/ Paediatrics
McMichael & Robinson (2016)	2008-2012	Drivers of sustained hygiene behaviour change: A case study from mid-western Nepal	Nepal	Australia	Geography
Nguon et al. (2018)	2016-17	Art and theatre for health in rural Cambodia	Cambodia	Cambodia	Epidemiology (Parasitology)
Olaide (2010)	Not stated	Maternal mortality and theatre intervention: A case study of Oluyole	Nigeria	Nigeria	Performing Arts

For peer review only

Sanghvi et al. (2013) Sanghvi et al. (2016) Nguyen et al. (2019)	2010-2016	Tailoring communication strategies to improve infant and young child feeding practices in different country settings; Achieving behaviour change at scale: Alive & Thrive's infant and young child feeding programme in Bangladesh; Information Diffusion and Social Norms Are Associated with Infant and Young Child Feeding Practices in Bangladesh	Bangladesh Ethiopia & Vietnam	USA	International Development
Sloand and Gebrian (2006)	1994-2005	Fathers Clubs to Improve Child Health in Rural Haiti	Haiti	US	Nursing
Thuita et al. (2015) Mukuria et al. (2016)	2010-12	Engaging fathers and grandmothers to improve maternal and child dietary practices: planning a community-based study in Western Kenya Role of Social Support in Improving Infant Feeding Practices in Western Kenya: A Quasi-Experimental Study	Kenya	Kenya/ USA	Public Health
Winter et al. (2021)	2017-2018	The potential of school-based WASH programming to support children as agents of change in rural Zambian households	Zambia	USA	Civil and Environmental Engingeering

For peer review only

Evaluation methods used	Intended Nurturing Care/Health Outcomes
Qualitative evaluation (interviews) of a multi-phase adaptation process for an intervention.	Early childhood development
Mixed-methods approach: literature review, community discussions & impact evaluation of an open-label controlled intervention trial	Knowledge about soil-transmitted helminth infections
Qualitative evaluation (interviews)	Health information retention; assessing contextual-appropriateness
Cluster randomised control trial evaluation of an intervention, interviews, and survey	Early breastfeeding initiation; exclusive breastfeeding
Randomised control trial and qualitative evaluation (caregiver interviews)	School readiness skills
Cluster randomised control trial evaluation of an intervention; Process evaluation and mixed-method analysis of an intervention adaptation process	Food hygiene behaviors
Impact assessment (interviews assessing knowledge and behaviour) comparing intervention with control villages	Control of malaria

For peer review only

Cluster randomized nonblinded impact evaluation and comparison between more and less intensive intervention packages, via cross-sectional household survey	Infant and young child feeding knowledge and practices
Cluster-randomised, controlled, non-blinded, impact evaluation (cross-sectional household survey and child stunting measurements)	Child undernutrition (via women's empowerment, IYCF knowledge and practices and child anthropometry)
sequential exploratory mixed-method study design (qualitative: process of digital story curriculum creation; quantitative: acceptability)	Maternal mortality rates
Cluster randomised control trial	Complementary-food contamination and disease rates
Qualitative case-study evaluation (interviews, group discussions, drawings and household observation)	Water and sanitation-related diseases
Description & Reflection on lessons learnt	Dissemination of key health messages and supplementing existing engagement activities conducted by local authorities
Descriptive case study evaluation	Maternal mortality rates

For peer review only

Cross country comparison of intervention design, adaptation phases and outcomes; Process description of multi-method intervention design and scale-up phases and evaluation via preliminary comparisons of intensive vs non-intensive treatment groups; Household surveys - analysis of paths from intervention exposure to IYCF practices through networks, diffusion, and norms (multiple [illegible]	Exclusive breastfeeding and complementary feeding practices
Literature Review	Child and family health
Quasi-experimental, non-equivalent comparison group design with pre- and post-testing (surveys, formative assessment and process evaluation); Quasi-experimental, multi-method, multi-phase intervention description and evaluation (survey)	Maternal and child dietary practices; Complementary feeding practices
Quasi-experimental, prospective cohort intervention impact evaluation (open and closed surveys)	Sharing of WASH-related messages (washing hands and using the latrine) with caregivers

For peer review only

Table 4: Prompt questions used for appraisal of the interventions during data synthesis

appraise **co-production** by:	appraise **contextual adaptation** by:	appraise **arts-based implementation** by:
Whose input was sought/heard at which stage?	How was the context attended to by different phases or forms of contextual learning (e.g. observations, focus groups)?	What type of dramatic arts were utilised and who delivered/facilitated their use?
Was there balanced and reciprocal participation of stakeholders, researchers, local agents and community members in design/ implementation decisions?	Was there feedback (formal or informal) between design and implementation phases?	Was the intervention flexible? in the room? Was it adaptable logistically? i.e. time and place of delivery (to improve attendance)
Were any tensions or differences in goals noted between partners? If so, were causes and/or resolutions explored?	Was there explicit incorporation of local knowledge, local infrastructure or networks, local history and social/economic/geographic/political differences or inequalities?<br>Note: beyond assuming this would happen automatically through participation of local people	Were any community groups excluded from participation? Were any groups notably less engaged, untrusting or sceptical upon delivery? Did any drop-out?
Intermediary-outcome hypotheses/ mid-range theories:<br>Is there evidence these brought about partnership synergy, partnership trust, targeting of equitably representative health/care/development issues, in a manner that are broadly supported and seen as effective. And, if so, to what extent?	*Intermediary-outcome hypotheses/ mid-range theories:*<br>Is there evidence these brought about effective, targeted health/care/ development information, and sustainable transmission of such information, equitable targeting of health/care issues. And, if so, to what extent?	*Intermediary-outcome hypotheses/ mid-range theories:*<br>Is there evidence these brought about explicit or experiential knowledge of health information, shared/communal experience of health information delivery, engaging with or challenging social norms, community alignment/ social binding or synergy, affective impact of health information delivery (e.g. motivation, shame, or empowerment). And, if so, to what extent?

For peer review only

Author (Date)	Co-production	Adaptation to context
Akter et al. (2020)	Researcher-led process, with feedback from participants, community members and community health workers.	Three existing interventions were integrated and culturally adapted via researcher knowledge and situation analysis (direct observation).<br><br>Two models of delivery were piloted - one in 'courtyard groups' and the other in individual home visits. Used local school infrastructure for delivery.
Al-Delaimy et al. (2014)	Researcher-led process, with engagement of a range of academic disciplines (e.g. public health, parascitology, education), local public health and community development officials, as well as residents, community members and service providers.<br><br>Limited opportunities for feedback during or after delivery.	Local adaptation of health education messages, via literature review and direct observation (household survey) by researchers of the Orang Asli communities, responding to a history of 'failed' interventions in the region.

For peer review only

Callery et al. (2020)	Researcher-led and stakeholder-funded. Involvement of a range of stakeholders at different levels, including Deputy District Governor, commune and village leaders, primary school principals, health centre chiefs, the police chief and village malaria workers.	A existing intervention was adapted to local context via a large stakeholder orientation meeting (n=~50) at a community health centre, where the activities, target villages and topics for community engagement were discussed.<br><br>This was followed by additional local, informal adaptation in each village, through meeting with commune and village leaders, the principal of the local school and village malaria workers to discuss with them the activities and inviting the students to participate.<br><br>TV was used to promote the events and celebrate.
Flax et al. (2014)	Researchers partnered with a US NGO (Partners for Development) and 4 local, Nigerian community-based organizations.<br><br>Community members were recruited into microcredit groups of 5-7 friends, neighbors, or relatives who guaranteed each other's loans.	The intervention was delivered within an established social/economic grouping - women's micro-credit groups - that met monthly.<br><br>It was unclear whether there was any local adaptation or feedback process to the intervention.
Goodman and Dent (2019)	Researcher-led process, whereby researchers identified the problem and designed the intervention then, using translators, trained the local project coordinators to deliver the intervention.	The team ensured that the intervention was low cost, and used local school infrastructure for delivery.

For peer review only

Gautam et al. (2017) Gautam and Curtis (2021)	Researcher-led process, drawing on literature and theoretical models of behaviour change (Behavior-Centered Design), but also engaging a range of stakeholders and local communities. Government and non-governmental organizations' advised that the intervention adapted from preceding interventions in Mali and India would be replicable and scalable in Nepal. A local creative team was assembled to design the intervention. Local women with a similar profile to Nepal's ubiquitous Female Community Health Volunteers (FCHVs)<br>an intervention package was designed and delivered, with a local creative team with expertise in marketing, design, innovation, program development, and [illegible]	The intervention design was informed by formative research conducted by 4 local research field data collectors and five local women from different castes/ethnic groups using hand held cameras, and analysed by the research team. Following this group discussions with community members and health officials.<br><br>Several pilots took place for feedback from those delivering the intervention and the communities. Final feedback was from government officials and NGO stakeholders.
Ghosh et al. (2006)	Led by an intersectoral committee (involving ten governmental and non-governmental organisations), and delivered by local agents. Funded by the government, whose ministers provided visibility for the programme. Religious leaders contributed by offering free accommodation and hospitality for the period of one month as a token of solidarity in the fight against malaria. Local press and radio helped in wider dissemination of health education messages.<br><br>Local and regional organisations supported the intervention, e.g. the education department deputed five teachers and the Child and Women's Welfare Department deputed ten Anganwadi workers for one month.	Informal adaptation through a two day visit from the intervention team prior to the final event.<br><br>Unclear if there were opportunities for feedback once the intervention had started.

For peer review only

Kim et al. (2018)	Donor-led initiative, working with researchers (from outside the country). The focus of the intervention shifted somewhat with new donors, who scaled up and adapted the programme. This is a top-down change (or feedback process).<br><br>The community-based infection and prevention control work was delivered by a international development organisation based in Bangladesh. Local health workers/volunteers/artists and regional/local infrastructres and organisations were used to deliver Infant and Young Child Feeding (IYCF) messages in regular meetings, community video and theatre shows, as well as radio and TV.	Broad consideration of context in intervention design (media, situation analysis, communications) concept testing, leading to creative development of prototype materials, (6) material pretests followed by revisions<br><br>The campaign implementation was subjected to ongoing monitoring, which was reported to lead to early and mid-course adjustments. However, evidence of bottom up changes/feedback incorporation in substantive content, or form of its delivery were not explicit.
Kumar et al. (2018)	International humanitarian organisation established partnerships with relevant government line ministries and with local implementing NGOs who were to deliver programme content across the different project components. Evaluation was conducted by International Food Policy Research Institute.	Used existing community health volunteers and women's groups, and existing government IYCF guidelines and materials to implement a gender-sensitive homestead food production programme with nutrition behaviour change communication.<br><br>Contextual issues were discussed in the paper and and initial gender analysis informed the intervention, but it is unclear whether there was any further adaptation informed by stakeholders.

Limaye et al. (2018)	Design of a digital story curriculum was participatory: the researcher ran three community-based PhotoVoice workshops with Community Health Workers and women who were invited by the CHWs; then they were involved in participatory digital storytelling activities. Stories were selected by the reserach team, who worked with a professional filmmaker and a professional midwife to develop these stories into a final digital story curriculum, where each story was combined with question-based teaching prompts.	The programme worked with existing community health workers, and the curriculum was iteratively developed with a variety of stakeholders.<br><br>There were limited opportunities later in the project for local storytellers to influence the final product (due to limitations of needing to process the digital stories in a city).
Manjang, et al. (2018) Manaseki-Holland et al. (2021)	Local researcher-led, with wide consultation with health promotion agencies who were represented on a Local Scientific Advisory Committee in the Gambia (MOH, Unicef, WHO, University of the Gambia, National Nutrition Agency (NANA) and the MRC Gambia).<br><br>Locally, traditional communicators, community leaders and traditional birth attendents or other experienced women/ grandmothers were engaged. Women's acheivements were celebrated individually (as MaaChampions) and communally (through villages achieving MaaChampion status).	The intervention was based on theoretical models of behaviour change and findings from previous similiar intervention studies (in Nepal and India) and were adapted to the local context, based on formative research and consultation with stakeholders. The intervention material was translated into the three local languages (Mandinka, Wolof and Fula), field-tested with mothers and villagers and piloted iteratively by the intervention team.<br><br>TV was used to promote the events.<br><br>Women's networks were used to convey the messages 'onward' from the intervention, finding more volunteers. These women visited the families between the visits from the wider team to help establish the practices within the cultural norms of the community.

McMichael & Robinson (2016)	Researcher-led, in partnership with Australian and Nepal Red Cross. Two non-local researchers led the data collection with the assistance of a Nepali bilingual research assistant and two Nepali interpreters (one male, one female). Local residents were involved in some elements, e.g. children in participatory hygiene education and activities, group/committee formation (e.g. junior/youth Red Cross circles, women's groups, Water User Committees) and employment of local Village Health Motivators to conduct community-based hygiene education and home visits. Significant changes were made as a result of community feedback: for example, the original intervention employed Community-Led Total Sanitation triggering tools to create demand for latrines and improved hygiene (e.g. "walk of shame", flagging faeces) which were found to be 'difficult' and removed when all used the toilets. Positive motivational drivers were reported to be more effective.	Feedback was sought in focus group discussions and in depth-interviews. These were used to evaluate intervention impact. Ongoing program monitoring was noted but is unclear how this may have impacted adaptation beyond the informal adpatation through participatory activities.<br><br>Arts-based elements used as part of a broader intervention programme addressing different dimensions of the socio-economic and public health context: including provision of partially subsidised hardware (toilets, handwashing platforms, construction/renovation ofwater sources etc.); group/committee formation (e.g. junior/youth Red Cross circles, women's groups, Water User Committees); hygiene and sanitation education; distribution of WASH information and education materials; employment of local Village Health Motivators to conduct community-based hygiene education and home visits; peer-led WASH awareness raising in schools/community; hygiene and
Nguon et al. (2018)	Led by local researchers, in partnership with the provincial health authorities, who selected and trained a local drama group to deliver the intervention based on a competitive process.<br><br>Community members were involved through attending the workshops, which led to the local tailoring of content for the public performances, and in quizzes/competitions.<br><br>High trust and synergy was reported between performers and the community.	This project (a pilot science-arts inititive) supplemented existing engagement activities conducted by local authorities and a community engagement team supporting a malaria elimination study (clinicaltrials.gov NCT01872702).<br><br>There was no formative research or formal adaptation process, but workshops were held in each village by the drama team, to adapt the performance using real stories of locals' experiences with malaria.

For peer review only

Olaide (2010)	Researchers, local drama groups, participants, stakeholders included government, health/medical and environmental establishment figures were involved in the design process.<br><br>The drama was intended to become the 'theatre of the people, by the people and for the people' (based on the Boalian concept of forum theatre) which aims not to find the 'best' solution but to produce a variety of options that could be used in the type of situation.	Communal research was conducted early in the process and identified that the most appropriate approach woudl be an 'inside-out approach' which would enable facilitators to interact within the community, gaining their trust and confidence and identify a 'joint panacea to the problems'.<br><br>The first step was the involvement of an existing and popular community-based theatre group in the dramatic endeavour data analysis, scenario development, improvisation rehearsal, performance and post performance discussion. Also involved are some individuals within the community in the play presentation and the relevant themes were discussed after the performance in a live Q&A the programme took three days to put together.
Sanghvi et al. (2013) Sanghvi et al. (2016) Nguyen et al. (2019)	Partnerships with stakeholders were vital to expand the program's reach and to sustain program activities after intitial funding ended. Partners were engaged in the research and design process to give implementing partners ownership of the strategy.<br><br>Government staff were trained by national and master trainers who were in turn trained by Alive and Thrive (A&T). The programme also launched a mass media campaign through inserts in newspapers (local journalists).	In each of the countries, Alive & Thrive convened stakeholders to review research to: (1) identify a few priority IYCF practices that would be the focus of the activities; (2) assess the degree to which select behaviors were feasible from family members' point of view and identified the conditions.<br><br>For all three countries, ProPAN methods were adapted and supplemented with additional tools, such as focus group discussions to gather information from frontline heath workers, fathers, grandmothers, and community leaders. The situational analysis in each country involved stakeholder consultations and reviews of existing data sets and reports to identify strategic choices and gaps in the data. Media audits, conducted by media research firms, guided two decisions: how much effort to devote to implementing or improving the Code of Marketing of Breast-Milk Substitutes, and what media channels to use for reaching different audience segments.

For peer review only

Sloand and Gebrian (2006)	Haitian Health Foundation (HHF), which is supported by both private donations and public funding, instituted fathers clubs in 1994. The fathers meet regularly, usually weekly or every 2 to 3 weeks, to learn about a wide range of child and family health topics and the related household- and family-level solutions. They discuss how to support their wives in the care of their children. They share family problems and help each other when needed. Meetings were described as 'very participatory'. The meetings are led by the fathers, but attended by health agents (community health workers) and often a nurse who supervises the health agent. Together, they provide health education and support to the fathers.	Topics are decided by group discussion and fathers also engage in wider local infrastructure/project issues.
Thuita et al. (2015); Mukuria et al. (2016)	International aid agency funded the project, which engaged volunteer community health workers (CHWs). These CHWs form community health committees and elect their leaders. In functional units, CHWs and committees are actively providing and overseeing community health surveys.<br><br>Each dialogue group selected one of its members to serve as the group mentor, who was trained and assisted by a volunteer CHW. Feedback was provided as well. Before each meeting, mentors selected the discussion topic based on member interest and then facilitated discussions and activities with group members.<br><br>Tea allowance was provided to all groups during meetings, however, fathers preferred money (100 ksh/ 1 USD).	Formative research designed key messages for fathers and grandmothers in households in western Kenya. Peer dialogue groups were used to facilitate behavior change among fathers and grandmothers by helping them gain new knowledge, share experiences and reflections, and apply communication and problem-solving skills that in turn would facilitate behavior change in mothers.<br><br>Existing health facilties were used to host events. Trained CHWs conducted home visits with study participants in both the intervention and comparison groups and shared oral and written complementary feeding and maternal nutrition messages. In the intervention areas, fathers or grandmothers were invited to participate in this visit.

For peer review only

Winter et al. (2021)	World Vision and Sesame Workshop collaboratively developed the WASH UP! program. WASH UP! combines a 12-week educational curriculum created by Sesame Workshop targeting students in grades 1-4 with school WASH infrastructure improvements carried out by World Vision.Teacher training is conducted by Sesame Workshop over the course of 3 days, and materials distribution and infrastructure improvements are funded and implemented by World Vision.The curriculum includes 12 sessions, each of which is approximately 30-40 min long and uses child-friendly pedagogical strategies such as play-based learning.<br><br>The twelve sessions were designed to be conducted before, during, or after normal school hours, at the discretion of the teacher. A learning object was distributed to a random sub-set of five of the 12 schools. Teachers provided the learning object to students with the instruction to take it home and discuss a key message about handwashing from the WASH UP!	helping families to establish and maintain handwashing stations close to the place of child feeding rather than focusing on the message 'wash your hands with soap'.<br><br>Mass media and community mobilization were used to create an enabling environment by changing perceptions about using soap as the norm. Communication materials and a TV spot focused on convenience - establishing a handwashing close to the place of food preparation. A training session on handwashing stations was also inserted in the national IYCF training manual.

For peer review only

Use of dramatic arts	What works, for whom, & in what circumstances? Was it sustainable?
Arts-based approaches were not part of the original intervention, but added after community feedback. Health messages were conveyed through use of visual cues, interactive role play and story-telling.<br><br>Delivered to communities by community health workers.	Participants reported willingness to attend the sessions if they were extended for 1 year. Changes recommended included additional visual cues and interactive role-play activities to make the sessions more engaging, a simplified behaviour-focused story (rather than the concept of 'unhealthy thoughts'), and to reduce the amount of content.<br><br>The study did not collect long-term data.
Posters/comic books, nursery rhymes and songs (lyrics on posters and songs on YouTube and school computers) and a puppet show were used alongside more traditional learning materials.<br><br>Delivered to child-participants by teachers, who were trained in relevant health content.	Knowledge and behaviour improved (with variable onset, longevity and extent), and school infrastructure was inclusive of range of children.<br><br>No long term outcomes, beyond 6 months (where control group had lower infections, but below statistical significance threhold). Long-term funding unclear.

For peer review only

Intervention was delivered by a community engagement team and members of a professional theatre group experienced in community/ village drama.<br><br>Participatory art and theatre workshops were delivered to school children to prepare for a village performance, which included wider members of the community. The workshops with schoolchildren were facilitated by teachers, with parental consent.<br><br>The performance included games, singing and dancing, health quizzes, a fashion show (showcasing traditional costumes, dance and muscial instruments) and a comedy sketch about malaria. The closing event involved speeches, competitions, performances from professional singers, local traditional dances and an amateur singing competition.	Qualitative interviews suggested that knowledge of health messages had improved, with general recall of messages but not all details. Some particpants shared the messages with non-participants. Involvement of children was noted to boost investment of families and teachers (and to require mastery of health messages for the performance).<br><br>Ethnic minority groups were specifically engaged and asked to be special guests at arts events in other villages. Showcasing elements helped the participants and the audience to gain a sense of ownership of the performances and this element was particularly popular among the local Kaviet and Lao populations.<br><br>No long term outcomes reported. Unclear whether further funding would be required or available.
Small groups of residents living near each other (in the microcredit groups) were asked to perform one of the key messages in a song or dramatic form.	Knowledge and behaviour improved, and intervention groups did breastfeed more at 3 and 6 months.<br><br>No longer term outcomes documented.
Storytelling and story-acting were used in the intervention. The story development was entirely child-led and 'spontaneous', integrated into play.	No difference overall between intervention and control group (who did drawing instead of story-telling). Some evidence that lower-income students and girls benefitted more.<br><br>No long term outcomes reported. It was unclear whether further funding would be required or available.

For peer review only

Local rallies, folk songs, games, rewards, storytelling, drama, cookery demonstrations and “kitchen makeover parties” were explicitly linked to the motivational drivers of behaviour identified in the guiding programme theory (Affiliation, Disgust, and Status).	The intervention changed five key behaviours observed in formative research to improve the microbiological environment. Women who engaged undertook public pledging and were celebrated with a public display of photos. The intervention was effective in differing socio-economic settings, although it is unclear if there were inequalities wihtin settings in the women who did and did not participate.<br><br>No long term outcomes observed.
Local educators, artists, religious leaders and community members collaborated to deliver the intervention.<br><br>Kalajatha is a popular, traditional art form of folk theatre depicting various life processes of a local socio-cultural setting . A local scriptwriter wrote 8 songs, two dramas and 4 rupakas (musical dramas) based on various aspects of malaria. These were then translated into skits using local dialects, musical styles and theatre traditions.<br><br>Thirty local artists (15 males and 15 females) from different occupational background were selected. Female artists were involved in the team, which resulted in better engagement from the women in the community.	Immediate behaviour changes were not evident but knowledge and attitudes of participants improved.<br><br>No long term outcomes were directly observed, but in a subsequent malaria prevention study via biocontol in the area the year after this intervention, a dramatic improvement in malaria incidence was observed and in the study area people still refer to the Kalajatha events and the messages delivered to them, suggesting long term knowledge retention.

For peer review only

Arts were only one component of a comprehensive programme of health promotion. Community video and theatre shows focused on infant and young child feeding. TV spots were broadcast nationally and regionally with intervention relevant messages.	This study offers evidence of decreased yet sustained impacts on IYCF practices even under modifications, suggesting program drift. Continued exposure infection prevention and control messages and and sustained impacts on infant and young child feeding practices indicate lasting benefits from these interventions, as they underwent major scale-up and adaptations after termination of initial external donor support. At 2 y after endline, intervention exposure had decreased, but home visits by frontline workers remained moderately high in the intensive areas and significantly higher compared with the nonintensive areas.<br><br>The programme is reliant on significant funding from external (non-governmenal and internation) organisations, so sustainability is dependent on this continuing.
Drama-based approaches were part of broader range of activities, including agricultural interventions (home gardening, providing seeds and equipment), and behaviour change components (group activities, radio messaging, posters, brochures) and women's empowerment (supply of fuel-saving stoves and fuel trees as labour-saving technologies). Community sensitisations were undertaken with assistance from drama groups, particularly around gender equality and its importance for improved nutrition. It is unclear whether these were [illegible]	Positive effects were observed on women's empowerment, IYCF knowledge, child morbidity and weight-for-height z-scores, but little impact was seen on IYCF practices, and no impact on stunting. Areas that could be strengthened were programme implementation and fostering higher participation rates.<br><br>Long term outcomes were not observed.

For peer review only

Photography (photo-voice) and community-based digital storytelling activities were used to inform the development of the curriculum. Participants were encouraged to think of personal stories and recorded themselves on mobile devices narrating their stories. Then participants did a storyboarding activity, in which they broke down the components of their story, and determined what types of photos would fit with each component ofthe story.	The digital story curriculum was highly acceptable to the target population, both men and women. They were considered novel, relatable, educational, and shareable.<br><br>Long term outcomes were not observed.
Performing arts (using culturally ingrained styles of drama and songs), animation, and stories.<br><br>Delivery team included traditional communicators (male and female, who use traditional African drumming, singing and acting), public health officers, a driver and a trained female volunteer, usually a traditional birth attendant (TBA).<br><br>Villagers reported fond memories of a 'joyous' programme with songs and activities of traditional performing artists, and they 'treasured' the achievements of MaaChampions in their family and community.	After intervention, behaviours improved (higher soap use in domestic kitchens and latrines) and there was a significant reduction in reported diarrhoea episodes and hospital admissions in the rainy season. No information was available about any inequalities in its effect.<br><br>Improved knowledge of the key behaviour messages was sustained at 6 months.<br><br>Qualitative data at 32 months suggested that mothers in intervention villages felt it their duty to inform mothers about complementary-food safety and hygiene practices when visiting other villages.

Children and community members were engaged through the arts to raise awareness of WASH - through street drama and song.	Qualitative evidence showed changes in hygiene knowledge, behaviour and development of new social norms around hygiene. There was some anecdotal evidence of improvments in health.<br><br>Contextual limitations included the prohibitative cost of further latrine construction, and an inconsistent water supply which limited flushing and handwashing practice.<br><br>This paper presents qualitative reports on the interventions impact two years after beginning. The project's impact appears to have been supported by the diverse and intensive behaviour change activities that were repeated over a long period time by peers as well as community leaders.
In each village there was a two and a half-day workshop led by a team consisting of local drama groups and health workers. There was a 'memorable' public performance on the third evening, incorporating local legends and real stories from the community. Community members, including children, were engaged using comedy, drama, drawing, singing competitions and quizzes about the intervention messages with prizes offered.	The drama storylines and health messages were remembered across the groups, and there was media interest in the project.<br><br>Attendence was lower proportionally in larger villages.<br><br>No long term outcomes reported.

For peer review only

Theatre performances, storytelling, later involved were drumming, singing and dancing which also made use of the songs that were known to them, most especially the nursing mothers and the pregnant women.	The study appears to have caught the attention of and galvanised the community around issues of maternal mortality based on the qualitative reports cited in the paper.
The performance comprised of 5 different but complementing sketches that were based on the causes and preventive measures of maternal mortality. The sketches were spiced up and linked together with different maternal songs that were folkloric, especially to the community members of the local government area.	The cost as with other local theatre groups should be low.<br><br>Long term outcomes were not observed.
In all three countries, the mass media campaign containing emotionally appealing mini-dramas with various messages to spread the perception of recommended practices as normative was designed for a run of at least 2 years to build up audience recognition and recall. The campaign's television ads were loaded onto DVDs and shown on screens in outlying areas with low electricity, using portable generators, and the frontline worker training included a session using the television spots. Rural communities in media dark areas engaged through other mediums such as theatre.	Local adaptation, engagement of multiple stakeholders and scaling up were key elements of these projects. Combining face-to-face interventions for mothers with social mobilization and mass media was effective in improving IYCF practices.<br><br>The broad campaigns may both help and hinder sustainability. Helpful in that broad audiences through information diffusion can help transform norms quickly for many. A hinderance in the complexity of messaging with locally adpated schedules, content as well as their organisation. Partnerships with local organisations potential helped sustain the programmes after initial funding.

For peer review only

The focus of meetings is usually a child or family health issue, and discussion, songs, and skits are used to enhance learning. To reach out beyond the group, they help organise village health fairs with mothers clubs or village health committees.	Fathers reported changes in knowledge, behaviour, social norms and skills, as well as health benefits (although these were not confirmed in observational studies). Father involvement is higher than expected based on other areas in Haiti. Fathers report believing that the health and well-being of all the village children is the shared responsibility of all the adults in the village.<br>It is unclear if there are inequalities in access or participation for fathers.<br><br>The Fathers Club project has been running since 1994 in Haiti, so can be considered very sustainable in this context. However, long term outcomes were not formally evaluated/reported.
Dialogue-based groups were used to engage infants' fathers and grandmothers to support optimal infant feeding practices. Group members participated in role plays, problem solving activities, storytelling, and cooking demonstrations; grandmothers also composed songs promoting recommended practices. They showcased what they were learning through songs, skits, dances, and testimonials from fathers, mothers, grandmothers, CHWs and children, at Community Bazaars.	Family-centred approach, through engaging fathers and grandmothers of infants to improve their knowledge of optimal infant feeding practices and to encourage provision of social support to mothers could help improve some feeding practices. It is unclear whether all groups were engaged in the activities. Arts were used for community mobilization activities.<br><br>The use of local infrastructure is promising for sustainability of the intervention. The bazaars are low cost also.

For peer review only

Activities include listening to the teacher read from a story book, playing interactive games, structured discussion of key curriculum messages, and watching two 10-min videos.	No evidence of effectiveness of children as agents of change.<br><br>Disruption to teacher's schedules and workloads were noted, this also was an image due to potenetial reputational damage for World Vision within such communities where they work. This necessitated some adaptation of scheudling of intervention delivery. Projectors and videos were included as part of the curricular materials, but three of the twelve schools did not have electricity, making one of the twelve sessions much more challenging to administer

For peer review only

**Scalability/ Transferability**
Small scale limits generalisability.<br><br>A systematic participatory approach was taken in intervention design, and a revised intervention was proposed which was likely feasible to deliver to a group of pregnant and lactating mothers in a low-resource setting.<br><br>Group sessions were as effective as combined ones and therefore potentially more cost-effective and scalable.<br><br>The intervention used teachers making it scalable, but how willing or able teachers are to help will vary between individuals and places. The arts elements depended on availability of school materials e.g. computers, so may not be reproducible in all contexts.

For peer review only

This intervention was an adapted reproduction of preceding interventions, so can be considered reproducible. No immediate barriers to scaling were noted.

The microcredit aspect of this intervention may be difficult without American developmental organisation support. Could also cause social disruption instead of solidarity if poorly managed. Other aspects are low-cost and use local organisations.

Storytelling is low-resource and but limited evidence of effectiveness. The use of local schools for delivery would be scalable.

For peer review only

That the intervention was equally effective across targeted behaviors and across clusters is a positive indicator that the improvements are reproducible. Healthworkers found that it was feasible to deliver the intervention on top of their existing workload, suggesting potential scalability.

This intervention was implemented through and with existing local and regional organisations. The theatre performances themselves should not be highly expensive and require few materials.

For peer review only

This project involved lots of government and non-government organisations. The practices of community arts-based health messaging is reproducible given low cost when working alongside existing organisations and with exisiting infrastructures.

In other contexts, where perhaps there was not be a broad political consensus on health priorities, expensive aspects of the current intervention may not be replicable, such as the TV ads. Reproducing this kind of comprehensive project at scale is challenging and demands significant collaboration.

The project was initially funded by international research organisations. It used local infrastructure and at times altered it, or offered incentives for participation (e.g. bicycles and/or agricultural inputs). The processes of adaptation to context (in design and delivery) are not very explicit.

For peer review only

Solar-powered mobile phones with cameras are becoming cheaper and so the participatory process for designing a curriculum in this way is perhaps likely more replicable over time. Reproducibility was not investigated directly, but if able to utlise existing networks of local health workers and secure funding, there are no obvious issues.

Cost-minimising tips were discussed toward replicability. Local structures and personnel used, with a low cost intervention.

This study builds on a smaller cluster RCTs of similar interventions in Nepal and India. This suggests the potential for reproducibility across different LMICs, and scope for scaling.

This low-cost intervention was not only acceptable to the mothers and the communities at large but was, at least in part, self-sustaining since new mothers reported practicing the behaviours at 32 months.

For peer review only

Small-scale and intensive nature limits the applicability of these findings to large-scale WASH interventions.

Latrine construction could not persist due to the cost.

Intervention attracted large audiences in the villages, and offered something unique to remote, rural villages - especially involvement of professionals and the opportunity for children to be performing on stage alongside professional actors and seeing themselves in newspapers and on television. This is likely reproducible in other contexts, but not necessarily scalable as it would need to be tailored.

For peer review only

No obvious barriers to scalability or reproduction were noted given low cost of theatre and success of other similar interventions.

The campaign was delivered at a large scale, reaching an esimateed 8.5 million. Scale-up was achieved through streamlining of tools and strategies, government branding, phased expansion through a local non-governmental implementing partner with an extensive community-based platform – and nationwide mainstreaming through multiple non-governmental organization and government programmes. The process has also been replicated in three countries. Without external (international) funding the scale of this study may not be replicable.

For peer review only

Low resources and low development context of this intervention imply high reproducibility across contexts. The use of existing health agents improves scalability also, however in contexts without a private/voluntary health foundation to provide support and organise reproducing and scaling may be more difficult.

Low cost and resource usage. Builds on existing infrastructure.

For peer review only

Low cost, low resource (is theatre less expensive than video? Likely more engaging, more easlily made participatory and local)

For peer review only

BMJ Open

Co-produced, arts interventions for nurturing care (0-5 years) in Low- and Middle-Income Countries (LMICs): a realist review.

Journal:	*BMJ Open*
Manuscript ID	bmjopen-2023-083093.R1
Article Type:	Original research
Date Submitted by the Author:	21-Mar-2024

Complete List of Authors:	Gale, Nicola; University of Birmingham<br>Ahmed, Kalim; University of Birmingham<br>Diarra, Niélé ; University of Bamako<br>Manaseki-Holland, Semira; University of Birmingham College of Medical and Dental Sciences, Department of Public Health, Epidemiology and Biostatistics<br>Asamane, Evans; University of Birmingham<br>Sidibé, Cheick; University of Bamako<br>Touré, Ousmane; University of Bamako<br>Wilson, Michael; Loughborough University<br>Griffiths, Paula; Loughborough University, School of Sport Exercise and Health Sciences

**Primary Subject Heading**:	Global health
Secondary Subject Heading:	Health policy, Paediatrics, Public health
Keywords:	Public health < INFECTIOUS DISEASES, Community child health < PAEDIATRICS, Systematic Review

SCHOLARONE™
Manuscripts

I, the Submitting Author has the right to grant and does grant on behalf of all authors of the Work (as defined in the below author licence), an exclusive licence and/or a non-exclusive licence for contributions from authors who are: i) UK Crown employees; ii) where BMJ has agreed a CC-BY licence shall apply, and/or iii) in accordance with the terms applicable for US Federal Government officers or employees acting as part of their official duties; on a worldwide, perpetual, irrevocable, royalty-free basis to BMJ Publishing Group Ltd ("BMJ") its licensees and where the relevant Journal is co-owned by BMJ to the co-owners of the Journal, to publish the Work in this journal and any other BMJ products and to exploit all rights, as set out in our licence.

The Submitting Author accepts and understands that any supply made under these terms is made by BMJ to the Submitting Author unless you are acting as an employee on behalf of your employer or a postgraduate student of an affiliated institution which is paying any applicable article publishing charge ("APC") for Open Access articles. Where the Submitting Author wishes to make the Work available on an Open Access basis (and intends to pay the relevant APC), the terms of reuse of such Open Access shall be governed by a Creative Commons licence – details of these licences and which Creative Commons licence will apply to this Work are set out in our licence referred to above.

Other than as permitted in any relevant BMJ Author's Self Archiving Policies, I confirm this Work has not been accepted for publication elsewhere, is not being considered for publication elsewhere and does not duplicate material already published. I confirm all authors consent to publication of this Work and authorise the granting of this licence.

For peer review only

TITLE

Co-produced, arts interventions for nurturing care (0-5 years) in Low- and Middle-Income Countries (LMICs): a realist review.

ABSTRACT

Objectives: Community-based arts interventions have the potential to support contextually relevant nurturing care programmes and policies that adapt to different settings. Understanding the distinctive features of using the arts in local, culturally-specific ways in Low and Middle Income Countries (LMICs); how this varies by context; and gaining a better understanding of the perspectives on desirable outcomes for communities is important evidence that this review generates.

Design: We conducted a realist review of papers that covered outcomes related to child health or development (0-5years) AND arts-based approaches AND community-based, participatory approaches AND based in LMICs using a range of databases and other networks. A coding framework was developed covering context, intervention, outcomes, mechanisms, study, sustainability, transferability, and scalability.

Results: The included papers reported 18 unique interventions. Interventions covered 14 countries, with evidence lacking for South America, Arab countries, and parts of Africa. Lead authors came from mostly clinical science-based disciplines and from institutions in a different country to the country/countries studied. Intended outcomes from interventions included clinical, health systems/organisation, changes in practices/behaviours/knowledge/attitudes, and wider social and educational goals. We identified three demi-regularities (semi-predictable patterns or pathways of programme functioning): participatory design based on valuing different sources of expertise; dynamic adaptation of intervention to context; and community participation in arts-based approaches.

For peer review only - http://bmjopen.bmj.com/site/about/guidelines.xhtml

For peer review only

Conclusions: Our findings suggest that arts-based, nurturing care interventions have greater potential when they include local knowledge, embed into existing infrastructures, and there is a clear plan for ongoing resourcing of the intervention. Studies with better documentation of the lessons learned, regarding the intervention delivery process and the power dynamics involved, are needed to better understand what works, for whom and in which contexts.

STRENGTHS AND LIMITATIONS OF THE STUDY

- Systematic search of the international literature ensured good coverage of the field.
- Multi-disciplinary team authorship ensured realist synthesis methods could capture what works, for whom and in what circumstances.
- Searches were conducted in English so the review may have regional biases, as well as positive results publication bias.
- Inclusion of studies that report interventions meant that relevant contextual or conceptual papers, books or chapters, more common in the arts disciplines were not included.

BACKGROUND

This article reports the findings from a realist review and synthesis of the published literature on community-based arts interventions targeted at improving health and development of 0-5 year olds in low and middle-income countries (LMICs).

Nurturing care:

UNICEF's nurturing care framework provides a structure through which countries or communities can support parents and caregivers to provide an environment that ensures optimal health, nutrition and development for infants and young children and in which these individuals can live free from

For peer review only - http://bmjopen.bmj.com/site/about/guidelines.xhtml

threats and have opportunities to develop and learn using responsive interaction [1]. More than 5 million children did not survive to their fifth birthday in 2020 [2]. Furthermore, 43% (over 250 million) infants and young children in LMICs do not achieve their developmental potential for reasons including poverty, undernutrition and a lack of appropriate responsive and nurturing care and simulation [3,4]. Appropriate nurturing care has the potential to reduce mortality and morbidity and improve early childhood development [5]. To support the core elements of the nurturing care framework, programmes and policies are needed that are contextually relevant and take account of the requirements and different needs of all communities, because nurturing care is heavily embedded in local cultures and those seeking to implement health or child development interventions are influenced by local political and economic circumstances [6,7].

Power and participation:

Community-based interventions are often co-produced with local communities. The term co-production is highly debated, but broadly refers to a collaborative and reciprocal process of exchange between actors of differing backgrounds, to generate outcomes uniquely embedded in the different perspectives shared [8]. There are often significant challenges in implementing co-production in practice, such as knowledge and power asymmetries between those collaborating. Often power dynamics in these collaborations shift away from local expertise to professional expertise meaning that not all voices are heard [9-14]. Such power dynamics may produce interventions which are not sensitive to the specific issues, barriers and affordances of the context in which the intervention is implemented.

Many have argued that to overcome challenges and realise the purported benefits of co-production, collaborators must move beyond a ‘tick box’ or process-driven approach to involvement to partnerships that enable partnership synergy [11]. This is built through mutual trust and understanding between parties and takes into account contextual factors [15,16]. Careful

For peer review only

For peer review only - http://bmjopen.bmj.com/site/about/guidelines.xhtml

consideration of how interventions may draw on and influence local infrastructures and relationships is also important, to build sustainable collaborations [17,18].

Arts-based approaches:

Applied arts is an umbrella term that refers to work that seeks to use the arts as a tool to be applied to a social issue; other commonly used terms in the literature are Arts for Development, Arts for Social Change or Participatory Arts [19,20]. Arts-based interventions are usually carried out in group settings where they are used to undertake collective processing and/or for the communication of new information such as health advice, e.g. through dramatic or musical performances, bazaar event days with activities, competitions and prizes. Applied arts approaches are distinct from therapeutic approaches, such as Art Therapy, which are associated with improving individual health directly [21].

When practiced through local arts traditions, an intervention is embedded into local culture rather than imposed from outside, which is typically thought to make the intervention more relevant, coherent and engaging to community members including using existing community strengths [22]. When both performer and audience are involved in a live performance it can act to bind communities with a sense of togetherness, increased trust and common goals [22,23].

Rationale for review and objectives:

Given the growing global interest in arts-based approaches to health and child development interventions, there is a clear need to understand whether they are effective and, if so, how they work. There is growing understanding of the effectiveness of arts-based approaches [23,24-30] , and much scholarship in this field from the arts disciplines engages with issues such the artistry or process of artistic performance or creativity, or the role of artistic practice in interrogating concepts or cultures [31,32], but arts scholarship tends to focus less on evaluating interventions *per se.* Systematic reviews of the effectiveness of arts-based interventions in child health and development in LMICs are lacking.

For peer review only - http://bmjopen.bmj.com/site/about/guidelines.xhtml

Realist evaluation methodologies aim to ensure that context is incorporated in any analysis of effectiveness, using the refrain, 'what works, for whom, and in what circumstances?' [33-35]. Interventions that seek to address issues of improving nurturing care are often complex, addressing many layers of social and cultural practice, and influenced by political and economic context, so oversimplistic assessments that deem such interventions categorically 'effective' or not are problematic. The nuanced realist evaluation of arts-based nurturing care interventions in LMICs provided in this review will provide evidence to inform policy and intervention design to improve nurturing care in some of the world's most vulnerable contexts. It will also explore the sustainability of interventions and generalisability of findings, and specifically whether interventions might be reproducible in other contexts (usually with some adaptations to context), or scalable to a wider population.

To address these gaps, we conducted the first realist review and synthesis of the published and grey literature on community-based arts interventions targeted at improving health and development for 0–5-year-olds in Low- and Middle-Income Countries (LMICs). We identify (i) possible intervention mechanisms focusing on the distinctive feature of using the arts in local, culturally specific ways; (ii) relevant issues of contextual difference and (iii) a broad perspective on desirable outcomes for communities, rather than focusing solely on medical outcomes. Realist review is an effective method for analysing complex interventions that are highly context-dependent [33,34]. This can help inform future research as well as policy and intervention design and implementation in this field, by identifying what works, for whom and in what circumstances, and what elements are likely to make interventions more sustainable, transferable, or scalable. Additional resources about realist reviews can be found the RAMESES Project website.

The overarching review questions are:

i. What kinds of co-produced, arts-based, nurturing care interventions have worked in LMICs, for whom and in what circumstances?

For peer review only

ii. To what extent are successful interventions sustainable, transferable, or scalable?

METHODS

We applied the RAMESES quality and reporting guidelines [35].

Scoping the literature:

We worked with a team in the UK and Mali, including the intended users of the review, to clarify focus and prioritize research questions. We presented our initial ideas and later our preliminary findings to the MaaCiwara project steering committee. MaaCiwara is an ongoing study with collaborations between institutions in Mali and the UK examining the impact of an intervention in Mali for nurturing care, with significant arts-based elements [36]. Our preliminary objectives for this review were to identify examples of arts-based interventions co-produced between communities affected by poor child health, nutrition, and development outcomes and those involved in the public health/health promotion sector in LMICs and/or research organisations; to develop an understanding of the theoretical frameworks that inform these interventions or can help explain their effects, and to understand the mechanisms that contribute to success for these interventions. As the review progressed, we introduced a second research question focused on the long-term potential of these sorts of interventions to have an impact within the original context and in other similar ones. The findings from this review have been used to inform the data collection and analysis strategy for the primary empirical research.

Search strategy:

We searched for peer-reviewed journal articles that covered outcomes related to child health or development (0-5years) AND arts-based approaches AND community-based, participatory approaches AND based in LMICs (Supplementary Table 1). An experienced information specialist performed these searches. These terms were used to search EBSCO, OVID, ProQuest, Scopus, Web of Science and Child Adolescence and Education Studies databases.

For peer review only

Selection and appraisal of studies:

KA and NHD independently screened titles and abstracts for relevance. Where there was uncertainty, further discussions took place with PG and NG and we refined our relevance criteria in line with the outcomes of these discussions (Supplementary Table 2). The gradual refinement of these criteria helped focus the review questions and ensured coherent conceptualizations of the research area between research team members.

Data Extraction:

Given the wide range of intended outcomes and evaluation methods used, we did not attempt to classify in any categorical way whether or not an intervention had been effective, rather we attempted to unravel whether the papers were able to say something about 'what works, for whom and in what circumstances' and whether improvements could be sustained. We did this by identifying and analysing context-mechanism-outcome configurations from information that was available in the papers.

To address the first research question, the team developed an initial coding framework:

- **Context:** Country-Region, Socio-Cultural, Economic-Developmental, Legal-Political, Geographical-Physical
- **Intervention:** Agents, Co-production, Arts-based Methods, Dates, Design, Implementation
- **Outcomes:** Impact on Primary Outcome; Impact on Secondary Outcomes, Unintended outcomes/consequences.
- **Mechanisms:** Observed-Evidenced; Theorised
- **Study:** Aims, Constructs-Variables, Date, Limitations, Sampling, Theoretical Framework, Study design/Methods, First Author Location
- **Paths:** Interaction between Context/Intervention, Context/Outcomes, Intervention/Outcomes, & Context + Intervention → Outcomes

For peer review only - http://bmjopen.bmj.com/site/about/guidelines.xhtml

170 The 'paths' extraction was based on anything in the paper that spoke direct to the issues of the Context-Mechanism-Outcome configuration [33], even if a realist approach was not used. After agreeing the approach, papers were coded and data were extracted independently by KA and NHD, who then had a series of discussions to resolve differences.

174 To address the second review question, a second round of data extraction was conducted by NG and PG on reported or potential issues of:

176 - **Sustainability**
177 - **Transferability**
178 - **Scalability**

179 *Data Analysis and Synthesis:*

180 Our process for generating theories and adjudicating between them involved four stages involving NG, KA and PG meeting regularly, with emerging themes discussed in wider team meetings. First, we worked to compare and contrast the different interventions and the evaluation methods used, with specific reference to the ways in which the interventions focused on the key concepts of our research question, i.e. 'co-production' and 'community arts-based'. Second, we looked for demi-regularities in the data [37] in order to generate a set of propositions about the social mechanisms at play in these interventions. Our discussions cohered around three themes for further exploration: developing sustainable partnerships; tailoring interventions to local context, and using arts-based approaches that engage with social norms. Third, we appraised each intervention with a series of questions centred on these three themes (Supplementary Table 3), in order to answer the question 'what works, for whom and in what circumstances?' Finally, we considered whether the interventions were reported to be or likely to be sustainable, transferable and/or scalable.

192 *Patient and public involvement:*

193 No patients or members of the public were involved in this literature review.

For peer review only - http://bmjopen.bmj.com/site/about/guidelines.xhtml

RESULTS

Study Characteristics:

Our database searches generated 940 unique results (Supplementary Table 4). After an initial screening of titles and abstracts, 175 results remained. At full text screening, 16 were deemed relevant to the review. Additionally, 7 papers from our informal search and consultation with the wider reference group were identified which met these inclusion and exclusion criteria and were added to our sample. Therefore, 23 papers were included in the review, which reported findings from 18 separate interventions (process overview in Supplementary Figure 1). In some cases, formative research or baseline characteristics were covered separately from evaluations of the effectiveness of the interventions resulting in multiple papers per intervention. The 18 interventions are summarized in Supplementary Table 5.

Global Coverage: These interventions covered 14 different countries, with the majority focussing on the Asia Pacific region (Bangladesh (n=3), Cambodia (n=2), Haiti, India, Malaysia, Nepal (n=2), and Vietnam), followed by the African region (Ethiopia, Kenya, The Gambia, Nigeria (n=2), Uganda, and Zambia (n=2)), and one paper from South America (Peru). This shows that the English language evidence base is not providing much evidence for the South American or Arab regions, as well as large parts of Africa.

Authorship Location and Disciplines: Given the focus on co-production in the review and the interdisciplinary nature of the interventions, there was a notable imbalance in the pattern of authorship. It was often unclear whether the projects had been initiated by those leading the publication or by others, such as local teams, but out of 23 papers, 17 have first authors affiliated to an institution in a different country to the country or countries being studied in the paper. In addition, most academic outputs were led from the health sciences and only one paper was led by

For peer review only - http://bmjopen.bmj.com/site/about/guidelines.xhtml

researchers in the arts and humanities and two by social scientists. One paper was led by a team in Civil and Environmental Engineering.

Intended outcomes: There were a range of different intended outcomes from interventions, from the clinical (e.g. maternal mortality rates, malaria rates, anthropometry), the organisational (e.g. utilisation of services), to changes in practices/behaviours (e.g. changed dietary practices, exclusive breastfeeding, sharing of messages within families), and changes in knowledge or attitudes (e.g. dissemination of messages, health information retention) and wider social and educational goals (e.g. women's empowerment, school readiness).

Evaluation methods: A range of qualitative and quantitative methods were used to evaluate the interventions, including cluster randomised controlled trials or impact assessments (non-blinded), quasi-experimental studies, a prospective cohort study, pre-post outcome evaluations (with or without control), cross-sectional surveys, interviews, focus groups and digital story creation. In a few cases, the focus was on descriptive case studies or process descriptions, rather than on evaluating outcomes.

Main Findings:

We identified three main demi-regularities: participatory design based on valuing different sources of expertise; dynamic adaptation of intervention to context; and community participation in arts-based approaches. These three pathways were unevenly realised across the different interventions described and evaluated in the papers (Supplementary Table 6) but can serve as a basis for future studies, or for those designing new or adapting interventions to reflect on.

Programme Theory 1: Participatory design based on valuing different sources of expertise

While our inclusion criteria required some element of 'co-production', there was a huge range of approaches, including those that were clearly researcher-led, with limited involvement of local people, local artists or other stakeholders, those that were driven by the interests of funders,

For peer review only - http://bmjopen.bmj.com/site/about/guidelines.xhtml

particularly NGOs, and those where local people, local artists or local researchers drove the projects forward. In some cases, the co-production elements were primarily enacted during the design stages [38], while others were mainly at the implementation stage through the adaptation of an existing intervention to local context [39]. Others provided continuing opportunities for communities to engage throughout the length of the programme [40].

Co-production models ranged from very light touch co-production models to co-production that started at the beginning of the project and followed throughout the programme of activities. At the lighter end was a model where community health volunteers talked with local communities in discussion groups to get their involvement in the intervention, but where the intervention was designed externally to the community [41]. Other lighter touch co-production models included a programme of activities developed outside of the community but delivered with local artists/ drama groups to engage communities in the production of the intervention success [42] or a model where an existing intervention was adapted with local community input [43]. At the other end of the spectrum, some projects used co-production models that started from the beginning of the project to design the intervention in partnership with the community [38], or where communities develop the materials and stories that drive the intervention’s focus messages [44], or where the intervention was framed around collective learning between artist and the community [40].

There were some limitations in the reporting of co-production approaches. Often there was very little critical reflexive elaboration about the power structures in which the interventions were being designed and implemented, or what the challenges and benefits of harnessing community power structures were (such as who was included and who was not in the process). Another area that was underdeveloped was to what extent the resourcing of the intervention or the evaluation influenced the co-production. It is likely that there were pragmatic requirements to deliver public health interventions for funders, combined with potent power dynamics in communities, which could intensify the exclusion of already ‘hard to reach’ groups.

For peer review only - http://bmjopen.bmj.com/site/about/guidelines.xhtml

Programme Theory 2: Tailoring of intervention to context

All papers recognised to some degree the need to develop interventions what were tailored to the local context and employed a range of different methods to do so. These ranged from formal formative research processes that used literature reviewing, as well as qualitative (interviews, visual methods) and quantitative methods (surveys, analysis of routine or existing data sets, media audits) to understand the pre-existing social norms and behavioural patterns related to nurturing care [43,45,46], or to identify key messages/changes to focus on. In other studies, a pilot or series of pilots were used to refine the intervention, drawing on feedback from those delivering, receiving, and funding the interventions [38].

Some interventions were built around existing community groups, such as 'Fathers Clubs' in rural Haiti, where the social groupings were already well established and health messages were incorporated into these in a way that was led by the fathers themselves, with support from health workers [47], or the women's groups in rural Zambia [45]. In addition, existing infrastructure was often used, such as health facilities or community meeting spaces [41,48].

Some papers acknowledged the importance of enabling or constraining environments, both physical (such as the location of handwashing stations or providing spaces for infant feeding) and not just focusing on health education messages. The importance of the social environment (such as the need to change perceptions about using soap in mass media campaigns) [49] was also identified. These approaches supplemented or complemented the arts components.

A significant limitation in the data was that authors did not always report on the opportunities for feedback from stakeholders, and so it is unclear whether those opportunities existed or not. Even where feedback opportunities are mentioned, it is not always clear what impact the feedback had on the subsequent delivery of the intervention, if any. Where performing arts were part of the intervention, there was very little reported in papers on how performing arts practitioners

For peer review only - http://bmjopen.bmj.com/site/about/guidelines.xhtml

contributed to the overall design or content of the performance, so it is hard to judge the extent to which they influenced the artistic approaches taken.

Potentially relevant to the context was other similar or related public health or NGO programmes that were being undertaken in the region, and any impact they could have on outcomes, but these were rarely reported.

<u>Programme Theory 3: Community participation in arts-based approaches</u>

Types of arts-based activities were wide-ranging and included: storytelling, story-acting, visual cues, interactive role-play, posters, comic books, nursery rhymes and songs, puppet shows, drama, drawing, singing competitions, animations, stories, tv promotions, games, dancing, fashion shows, comedy sketches, speeches, professional singing performances, street drama, skits (sketches), kitchen makeovers, decorating kitchens, letter exchanges, family drama, folk songs, cookery demonstrations, photo displays, Kalajarta (folk theatre), rupakas (musical dramas), writing scripts, community videos, TV spots with mini-dramas of intervention messages, radio messaging, brochure design, digital storytelling, drumming, testimonials, cartoon films, mass media campaigns, game based and music based education, recitals, storybook reading, interactive games, and watching videos.

Less than half of the interventions delivered the arts components in a way that included specialist arts practitioners, such as local drama groups, traditional communicators, traditional theatre groups, songwriters, photographers, and other artists. Most interventions delivered the arts components through or with other members of the community, such as community health workers (normally with basic education), parents (mostly mothers) and wider community groups. In some cases, workers from NGOs were used to deliver intervention materials. It was much less common for papers to mention local or national government agents as involved in intervention delivery.

For peer review only - http://bmjopen.bmj.com/site/about/guidelines.xhtml

Very few papers commented on their theories or principles of how the arts were expected to support the intervention, with the exception of Olaide's paper (written by a Performing Arts scholar) which noted that it drew on Boal's concept of 'forum theatre' which is part of a broader concept of 'theatre of the oppressed', which is a widely used tool particularly in India, and eastern and southern Africa to facilitate social change [50]. Most studies were reported by health scientists and in health journals so in very few cases was a detailed understanding of the process of arts production or performance interrogated in a critical or scholarly way. The intended mechanisms of change remained implicit, the arts were reported as a 'black box' conceptually and practically (no knowledge of the internal workings).

Other limitations to the reporting of the arts elements of the interventions included few detailed analyses of who was involved in the activities, how they were invited, and which parts of the communities may have been excluded for cultural, social, political, or economic reasons.

Finally, there was little consideration of how the arts activities aligned or not with broader community activities or norms, such as the provision of hospitality.

To what extent were the interventions sustainable?

Approximately half of the studies did not record long-term outcomes which makes it difficult to judge their sustainability. There are of course issues with measuring effectiveness long-term, such as leakage of intervention into control groups [45,51]. Key to sustainability is whether change can be maintained after the withdrawal of the research team [52].

A number of the studies claimed that the engagement of local arts meant that the studies would be more sustainable. The reasons given were that the arts interventions promoted willingness to engage [53] or included celebration as a motivation for continuing [51]. Where interventions drew on local resources, this was often considered to make them more sustainable or low cost [40,43,48,51,54,55]. Others argued that the use of arts would be better for promoting

For peer review only - http://bmjopen.bmj.com/site/about/guidelines.xhtml

retention/memory of the intervention messages [51,56] and that arts-based interventions help to create a positive attitude and contribute to people being more friendly to each other to promote sustainability [40]. Positive motivational drivers particularly embedded in arts-based messaging was seen to be more effective than negative drivers such as issuing defecation fines [39].

Other themes on claims for sustainability were that the diverse and intensive nature of activities over time promoted sustainability [39], and that interventions were more realistic to implement when they were embedded in local infrastructure and resources so that people can access the intervention [44,45]. It was, however, also noted that ensuring that those delivering the intervention are not distracted from their other duties when drawing on local infrastructure was also important, as well as ensuring appropriate additional incentivisation for involvement [45,49]. Local infrastructure issues, such as a lack of electricity or equipment, made the delivery of materials such as videos challenging and affected sustainability [49]. A final issue in sustainability was whether other members of the family and community (beyond the mother) were brought in to engage with the intervention [41] because broader family support was reported to bring longer-term sustainability of messaging.

To what extent were interventions transferable?

Systematic participatory approaches simplify the process of contextual adaptation[53] enabling the process of developing and delivering the intervention to be replicated (even if the content is different). Interventions that use existing staff, e.g. teachers or community health workers [38,47], to deliver the intervention find that this promotes transferability into contexts where the health or education systems [55] or infrastructure [44,45] are similar and these professionals or paraprofessionals receive broadly similar training. However, there is always a risk that staff groups in different contexts may not buy into the intervention. In addition, varying relationships between governments and NGOs were a limitation to transferability in some contexts [52].

To what extent were interventions scalable?

For peer review only - http://bmjopen.bmj.com/site/about/guidelines.xhtml

Small-scale interventions can limit the potential for scalability[39] particularly where they are reliant on individual motivation and momentum. Broad campaigns that capture large audiences quickly are more likely to be scalable, but the specificity of messaging can be challenging [57], which may limit the effectiveness or traction of the messages. Elements of group interaction also maximise the efficient use of resources [53], making the intervention more scalable, as well as having the potential to enhance community ties. A further point raised on scalability of arts-based interventions was when tried and tested health intervention materials were used alongside arts-based approaches leading to improved buy in for scaling [58].

Major limitations around scalability within LMICs are cost and resource, with many of these interventions seeking to minimize the resource burden on government or communities [40,41,43,47,52,55,56]. Examples of this are using local storytelling [59] or theatre [56, 58] which are cheaper than the production costs of video making [49]. Some costs can be prohibitive for scaling e.g. TV ads [52] and cameras on smartphones [44]. For this reason, NGO support and finances can be critical requirements for scalability [46,47,54-56, 60].

DISCUSSION

Summary of findings:

We identified three main programme theories, suggesting the importance of (i) participatory design based on valuing different sources of expertise; (ii) tailoring of the intervention to the local political and cultural context, and (iii) community participation in arts-based approaches. We found mixed evidence on sustainability of interventions, but that those embedded in local infrastructure and culture were more likely to be sustainable. We found that transferability of the interventions to other contexts, depended on the social and political organisation of health and child development activities (such as schools and community health workers). We found that the scalability of the community-arts interventions was variable, depending on the scale and levels of integration of the

For peer review only - http://bmjopen.bmj.com/site/about/guidelines.xhtml

arts approaches within wider programmes of health and child development work as well as the availability of financial resources.

Contribution to the literature (empirical):

Our findings on community-based arts interventions targeted at improving health and development outcomes for 0-5 year olds in Low and Middle Income Countries (LMICs) have revealed a number of gaps in current knowledge and understanding of what works for whom in which contexts.

The literature on interventions currently lacks input and leadership from academics working in the arts, humanities, and the social sciences. The lack of inclusion of arts scholars means there is a lack of theory integrated into discussions on how the arts were expected to support the interventions delivered, and it is unclear what was actually done in practice with local artists. Arts scholarship does engage with some of these issues [31] but not always in an interdisciplinary way where it is linked to outcomes of interventions (hence not meeting our review inclusion criteria). The lack of social science input likely leads to a dominant focus of the literature on outcomes as opposed to the processes used to achieve these. While there are literatures that suggest that the arts have the potential to break down power dynamics and provide communities a sense of ownership of the intervention with audiences owning the art or the performance [61-63], these critical approaches were not used to interpret the findings for these interventions. This means that opportunities to learn about what works for whom and in which contexts were missed.

The lack of local lead authors of studies and insufficient studies from some regions including West and Southern Africa and South America means that contextual understanding is often lacking in the literature reviewed and this gap is particularly big for the regions with no pre-existing evidence.

The majority of the studies included in this review used a model of co-production that involved bringing in communities to adapt existing interventions at a later stage of the development process as opposed to including communities from conceptualisation. The minority of studies that did work with communities throughout the process to co-produce were able to document benefits including

For peer review only

time to build trust, community capacity, empowering local leadership, and developing a true collaboration. These factors were all relevant for intervention sustainability because they may empower the community to take the intervention forward after the study ends. Overall, these elements were poorly reported, although this may reflect the focus and limited words counts in target journals.

Studies that entered communities early to co-design were better placed to draw on existing health systems including community health workers, social infrastructures such as fathers' or mothers' groups, and community facilities such as community spaces. Using existing infrastructures saves investment in time and resources to build and grow social systems and staffing infrastructures to support these types of intervention and supports sustainability. Studies that did use existing structures reported the importance of the level of adequate functioning of these systems to positively influence intervention outcomes. Ongoing training of workers in the system was also reported to be important to maintain the success of intervention messaging and to support intervention sustainability beyond the study. This review also identified studies that did not report interaction with existing community structures such as health systems which was a missed opportunity for joined up thinking and promoting intervention sustainability.

Contribution (theoretical)

The interrelatedness and interdependence of the three themes (co-production, local adaptation, and arts-based delivery) underpins the theoretical contribution of this review. While co-production and local adaptation offer two elements of 'scaffolding' that are necessary but not always sufficient for change, our findings suggest that the arts-based delivery approach can act as a bridge for overcoming some of the contextual and political challenges of delivering nurturing care interventions in LMICs. The use of realist methods for synthesizing the literature allowed us to explore multiple and intersecting mechanisms of action, and to explore those mechanisms within different contexts. It allowed us to see that the success of the arts elements is dependent on

For peer review only

For peer review only - http://bmjopen.bmj.com/site/about/guidelines.xhtml

effective collaboration and adapting to context, and to see that the arts can enhance and enact the benefits of collaborative working and careful local adaptation processes. Nevertheless the lack of explicit reporting in papers on the mechanisms through which the arts work to enact change means that we are left with limited knowledge about what works for whom in what context. For instance we have learned little about audience involvement in the arts, cultural embeddedness of the arts, how the arts works most effectively with the public health services, how the arts messages can be effectively shaped by those who know the health elements, the role of knowledge and background in the health topic of the artists, inclusion or inclusion of certain population groups through the arts, the role of the arts venues, and how arts interact with social norms. All of these would be important to gain understanding of the true role of the arts in facilitating nurturing care.

There is evidence to suggest the importance of working toward synergy between partners[11] in both goals and processes [64], and of including local people, community leaders, public health and education professionals, policy-makers, and researchers, as well as funders of nurturing care interventions, in order to achieve more than each could alone [65]. Our findings supported this through stressing the value of regular formal [53] or informal feedback opportunities [40] and regular participatory meetings to support sustainability [47]. This builds trust [15,66] and transparency [67,68].

When collaborations also acknowledge that interventions must be tailored to context in both the design and implementation phases, more can be achieved. Complementing or integrating with existing educational and health infrastructures and human resources was identified as being key, as was an understanding of cultural and social norms [39,42,49] for sustainable change.

However, a key risk to effective co-production and tailoring to local context is the dysregulating effect of power imbalances. Enabling empowerment of those with less historical or cultural power in collaborations is a vital element of success [16]. The review has highlighted how challenging it is for collaborations to achieve high levels of balance and equity in contributions, and how rare it is for

For peer review only - http://bmjopen.bmj.com/site/about/guidelines.xhtml

these issues to be explicitly or critically reported. Our analysis can make an additional theoretical contribution here by identifying the potential for arts-based approaches to act as a bridge for some of these challenges of power imbalances through practical and political pathways.

First, *practically*, our findings suggest that engagement with community arts increases the potential effectiveness of the intervention in achieving (shared) goals of improved health and child development. Both the arts and community aspects support engagement in a shared experience of performance and through the congruence of local arts with local culture. Our findings suggest that there are two elements to this: increasing motivation to engage, and shifting long standing social norms. Positive motivation is engendered though things like a festival-like atmosphere, diversity of art forms, and the focus on positive impacts of change. The social dimensions of motivation may be particularly encouraged by communal experiences which contribute to group binding [22,23]. The use of local arts, local performers and organisers [56,58] and even local stories [42] increases the sense of ownership [40]. Interventions that are able to acknowledge and then shift long-established social norms from within are much more likely to be sustainable, rather than introducing external ideas. While the studies in the review rarely explicitly commented on social norms, our analysis was able to identify possible elements of the individual, social, material and institutional domains [69] in which social norms operate. The active engagement and the shared memories of an event or experience were considered to promote discussion of intervention materials, improve its retention individually and, importantly, its diffusion through social networks, reaching beyond intervention participants [51,56].

Second, *politically,* our findings suggest that these sorts of community arts-based delivery approaches, when combined with co-production and local adaptation, have the potential to disrupt some of the neo-colonial tendencies in global health research and practice. This involves shifting the balance of power away from Western biomedical models, towards communities and their historical and cultural modes of artistic expression and cultural re/production. Culturally embedded arts as

For peer review only - http://bmjopen.bmj.com/site/about/guidelines.xhtml

opposed to non-culturally embedded arts have the most potential to achieve this outcome. Even the frequent delivery of these interventions in local languages suggests they are at least partially outwith the surveillance of global health researchers. While in some cases attempts to address power imbalances were evident, such as minority outreach and platforming during performances [59]; the targeting of women's empowerment through agricultural work and education [45]; targeting father and grandmother involvement in nurturing care practices [41,48], or setting up partnership agreements [70], in many cases this was not addressed explicitly in the papers.

Strengths and limitations of the review

The review only captures published peer-reviewed journal articles. This likely results in a positive results publication bias and a lack of reports on arts-based interventions (grey literature). While we did not explicitly exclude French and Spanish articles (we had the language capacity in the team), we conducted the searches in English only. Despite searching a wide range of databases, we have identified that the papers that have been included are focussed on health sciences rather than the arts/social science parts of the research. Many arts scholars publish in monographs or edited collections which are not always effectively captured through database searches.

Recommendations for further research

There is a significant gap in the international literature, for studies that are led by researchers from LMICs and/or by those in the arts, humanities and social sciences. Bringing these voices into the research would enable better integration of the theoretical underpinnings regarding the potential success of arts-based intervention processes in nurturing care interventions. It would also facilitate better understanding of intervention processes and cultural context of these. Specific steps could be considered to ensure this can happen, such as leadership from LMICs, devolving funding decisions, and supporting and funding projects led from the arts, humanities and social sciences as well as the health and clinical sciences. Methods for ongoing reflective evaluation of the quality and equity in collaborations would be useful. Contextual issues may be relevant, including sources of funding

For peer review only - http://bmjopen.bmj.com/site/about/guidelines.xhtml

being weighted toward health funders and the motivation for academics to publish in high impact journals, which tend to be more STEMM-focused. Finally, reporting in journals of interventions with arts should include better documentation of their learning regarding the process of intervention delivery and the power dynamics involved in this.

Recommendations for policy and practice

Nurturing care interventions are complex involving the health system, early education providers, social workers, families, extended families and community leadership structures. As we have shown in this review there are attempts taking place to incorporate the practical and political power of the arts into intervention design to empower communities to own nurturing care interventions, to facilitate message sharing and to embed intervention messaging into the culture of communities. Our findings suggest that these types of intervention have the potential for greater success in being contextually relevant when they include local knowledge including artists, are evaluated by teams that include those with experience in the arts, include collaborative work between artists and public health experts to adapt and deliver the intervention, and where local researchers take a lead or active role in the intervention delivery, evaluation and reporting.

There is also evidence in this review that where arts-based interventions embed into existing infrastructures (e.g. health services) and there is a clear plan for ongoing resource for training of those delivering the intervention that sustainability is likely. To better understand what works for whom in which contexts when designing nurturing care arts-based interventions there is a need for studies to better document their learning regarding the process of intervention delivery and the power dynamics involved in this.

CONCLUSIONS

The findings of this realist review contribute to our understanding of the potential impact of co-produced, arts interventions for nurturing care in LMICs. Using arts approaches had the potential to act as a bridge for some of the challenges of power imbalances between researchers, health and

For peer review only - http://bmjopen.bmj.com/site/about/guidelines.xhtml

education professionals, and communities, that tailoring interventions to context and participatory design could not achieve alone. Interdisciplinary investigations integrating insights from arts scholarship and from the public health fields could support future research.

Footnotes:

Acknowledgements: In addition to our core research team, we would like to thank Rachel Posaner for her work developing and executing the search strategies for this paper. We would also like to thank the wider MaaCiwara team for their support.

Contributors: NG: Securing funding; study design; data collection, extraction and analysis; manuscript drafting; manuscript development and review. KA: Study design; data collection, extraction and analysis; manuscript drafting; manuscript development and review. NHD: Data collection, extraction and analysis; manuscript development and review. SMH: Securing funding; manuscript development and review. AE: Manuscript development and review. CS: Securing funding; manuscript development and review. OT: Securing funding; manuscript development and review. MW: Manuscript development and review. PG: Securing funding; study design; data collection, extraction and analysis; manuscript drafting; manuscript development and review.

Funding: This research was funded by Medical Research Council (MRC), UK Research and Innovation (UKRI) Global Challenges Research Fund (GCRF) MR/T030011/1. The funder of this study has no role in the design, conduct, collection of data, analysis or writing of outputs.

Competing interests: None declared.

Provenance and peer review: Not commissioned; externally peer reviewed.

Data sharing statement: All papers included in the review are available on the journal websites.

ETHICS APPROVAL

For peer review only

For peer review only - http://bmjopen.bmj.com/site/about/guidelines.xhtml

Ethical approval was not required for this literature review.

REFERENCES

1. Britto PR, Lye SJ, Proulx K, *et al.* Nurturing care: promoting early childhood development. *The Lancet*. 2017;389:91–102. doi: 10.1016/S0140-6736(16)31390-3

2. UNICEF. Levels and trends in child mortality. United Nations Inter-agency Group for Child Mortality Estimation 2021. https://data.unicef.org/resources/levels-and-trends-in-child-mortality/ (accessed 21 August 2023)

3. Lu C, Cuartas J, Fink G, *et al.* Inequalities in early childhood care and development in low/middle-income countries: 2010–2018. *BMJ Global Health*. 2020;5: doi:10.1136/bmjgh-2020-002314

4. Richter LM, Daelmans B, Lombardi J, *et al.* Investing in the foundation of sustainable development: pathways to scale up for early childhood development. *Lancet*. 2017;389:103–18. doi:10.1016/S0140-6736(16)31698-1

5. UN. Sustainable Development Goals. 2018. https://www.who.int/europe/about-us/our-work/sustainable-development-goals (accessed 21 August 2023)

6. Barnish MS, Tan SY, Taeihagh A, *et al.* Linking political exposures to child and maternal health outcomes: a realist review. *BMC Public Health*. 2021;21:127.

7. Barnish MS, Tan SY, Robinson S, *et al.* A realist synthesis to develop an explanatory model of how policy instruments impact child and maternal health outcomes. *Social Science & Medicine*. 2023;339:116402.

8. Durose C, Perry B, Richardson L. Is co-production a ‘good’ concept? Three responses. *Futures*. 2022;142: doi:10.1016/j.futures.2022.102999

For peer review only

For peer review only - http://bmjopen.bmj.com/site/about/guidelines.xhtml

9. Egid BR, Roura M, Aktar B, *et al.* 'You want to deal with power while riding on power': global perspectives on power in participatory health research and co-production approaches. *BMJ Global Health*. 2021;6: doi:10.1136/bmjgh-2021-006978

10. Yassi A, Spiegel JB, Lockhart K, *et al.* Ethics in Community-University-Artist Partnered Research: Tensions, Contradictions and Gaps Identified in an 'Arts for Social Change' Project. *J Acad Ethics*. 2016;14:199–220. doi:10.1007/s10805-016-9257-7

11. Keneth, Bamuturaki. Participatory Research in Theatre for Development: An Evaluative Paradigm of the Walukuba Project in Eastern Uganda. *Consciousness, Literature and the Arts*. 2016;17.

12. Goncalo JA, Duguid MM. Follow the crowd in a new direction: When conformity pressure facilitates group creativity (and when it does not). *Organizational Behavior and Human Decision Processes*. 2012;118:14–23. doi: 10.1016/j.obhdp.2011.12.004

13. Bechtoldt MN, De Dreu CKW, Nijstad BA, *et al.* Motivated information processing, social tuning, and group creativity. *Journal of Personality and Social Psychology*. 2010;99:622–37. Doi: 10.1037/a0019386

14. Schulz AJ, Israel BA, Lantz P. Instrument for evaluating dimensions of group dynamics within community-based participatory research partnerships. *Evaluation and Program Planning*. 2003;26:249–62. doi:10.1016/S0149-7189(03)00029-6

15. Jagosh J, Bush PL, Salsberg J, *et al.* A realist evaluation of community-based participatory research: partnership synergy, trust building and related ripple effects. *BMC Public Health*. 2015;15:725. doi:10.1186/s12889-015-1949-1

16. George AS, Mehra V, Scott K, *et al.* Community Participation in Health Systems Research: A Systematic Review Assessing the State of Research, the Nature of Interventions Involved and the Features of Engagement with Communities. *PLOS ONE*. 2015; doi: 10.1371/journal.pone.0141091

For peer review only

For peer review only - http://bmjopen.bmj.com/site/about/guidelines.xhtml

17. Kohrt BA, Asher L, Bhardwaj A, *et al.* The Role of Communities in Mental Health Care in Low- and Middle-Income Countries: A Meta-Review of Components and Competencies. *International Journal of Environmental Research and Public Health*. 2018;15:1279. doi:10.3390/ijerph15061279

18. Greenhalgh T, Jackson C, Shaw S, *et al.* Achieving Research Impact Through Co-creation in Community-Based Health Services: Literature Review and Case Study. *The Milbank Quarterly*. 2016;94:392–429. doi:10.1111/1468-0009.12197

19. Stupples P. Creative contributions: The role of the arts and the cultural sector in development. *Progress in Development Studies*. 2014;14:115–30.

20. Cooke P, Soria-Donlan I. *Participatory Arts in International Development*. 1st ed. Routledge 2019.

21. Slayton SC, D'Archer J, Kaplan F. Outcome Studies on the Efficacy of Art Therapy: A Review of Findings. *Art Therapy*. 2010;27:108–18.

22. Stewart L, McConnell BB, Darboe B, *et al.* Social singing, culture and health: interdisciplinary insights from the CHIME project for perinatal mental health in The Gambia. *Health Promotion International*. 2022;37:i18–25.doi: 10.1093/heapro/daab210

23. Faigin D, Stein C. Community-Based Theater and Adults with Psychiatric Disabilities: Social Activism, Performance and Community Engagement. *American journal of community psychology*. 2014;55. doi:10.1007/s10464-014-9695-6

24. Barnish MS, Barran SM. A systematic review of active group-based dance, singing, music therapy and theatrical interventions for quality of life, functional communication, speech, motor function and cognitive status in people with Parkinson's disease. *BMC Neurology*. 2020;20:371.

25. Barnish MS, Nelson-Horne RV. Group-based active artistic interventions for adults with primary anxiety and depression: a systematic review. *BMJ Open*. 2023;13:e069310.

For peer review only - http://bmjopen.bmj.com/site/about/guidelines.xhtml

26. Logie CH, Dias LV, Jenkinson J, *et al.* Exploring the Potential of Participatory Theatre to Reduce Stigma and Promote Health Equity for Lesbian, Gay, Bisexual, and Transgender (LGBT) People in Swaziland and Lesotho. *Health Educ Behav*. 2019;46:146–56. doi: 10.1177/1090198118760682

27. Torrissen W, Stickley T. Participatory theatre and mental health recovery: a narrative inquiry. *Perspect Public Health*. 2018;138:47–54. doi:10.1177/1757913917723944

28. Gallant K, Litwiller F, Hamilton-Hinch B, *et al.* Community-Based Experiential Education: Making It Meaningful to Students Means Making It Meaningful for Everyone. *SCHOLE: A Journal of Leisure Studies and Recreation Education*. 2017;32:146–57. doi:10.18666/SCHOLE-2017-V32-I2-3

29. Putland C. Lost in Translation: The Question of Evidence Linking Community-based Arts and Health Promotion. *J Health Psychol*. 2008;13:265–76. doi:10.1177/1359105307086706

30. Rossiter K, Kontos P, Colantonio A, *et al.* Staging data: Theatre as a tool for analysis and knowledge transfer in health research. *Social Science & Medicine*. 2008;66:130–46.doi: 10.1016/j.socscimed.2007.07.021

31. Baxter V, Low K. *Applied Theatre: Performing Health and Wellbeing*. 1st ed. Bloomsbury Publishing 2017.

32. Sextou P. A Theatre-in-Education Study of Multicultural Understanding in Hellas. *Youth Theatre Journal*. 2006;20:77–93.

33. Pawson R, Tilley N. An introduction to scientific realist evaluation. *Evaluation for the 21st century: A handbook*. 1997;405–18.

34. Jagosh J, Pluye P, Wong G, *et al.* Critical reflections on realist review: insights from customizing the methodology to the needs of participatory research assessment. *Research Synthesis Methods*. 2014;5:131–41. doi:10.1002/jrsm.1099

For peer review only

35. Wong G, Greenhalgh T, Westhorp G, *et al.* RAMESES publication standards: realist syntheses. *BMC Medicine*. 2013;11:21. doi:10.1186/1741-7015-11-21

36. Asamane EA, Quinn L, Watson SI, *et al.* Protocol for a parallel group, two-arm, superiority cluster randomised trial to evaluate a community-level complementary-food safety and hygiene and nutrition intervention in Mali: the MaaCiwara study (version 1.3; 10 November 2022). *Trials*. 2023;24:68.

37. Sayer A. *Realism and Social Science*. London: SAGE Publications Ltd 2000. doi: 10.4135/9781446218730

38. Gautam OP, Schmidt WP, Cairncross S, *et al.* Trial of a Novel Intervention to Improve Multiple Food Hygiene Behaviors in Nepal. *Am J Trop Med Hyg*. 2017;96:1415–26.

39. McMichael C, Robinson P. Drivers of sustained hygiene behaviour change: A case study from mid-western Nepal. *Social science and medicine*. 2016;163:28.

40. Olaide NT. Maternal mortality and theatre intervention: A case study of Oluyole local government in Ibadan of Nigeria. *US-China Foreign Language*. 2010;8:52–62.

41. Thuita FM, Martin SL, Ndegwa K, *et al.* Engaging fathers and grandmothers to improve maternal and child dietary practices: Planning a community-based study in western Kenya. *African Journal of Food, Agriculture, Nutrition and Development*. 2015;15:10386–405. doi:10.4314/ajfand.v15i5

42. Chea Nguon, Lek Dysoley, Chan Davoeung, *et al.* Art and theatre for health in rural Cambodia. *Global Bioethics*. 2018;29. doi:10.1080/11287462.2017.1411762

43. Buba Manjang, Hemming K, Bradley C, *et al.* Promoting hygienic weaning food handling practices through a community-based programme: intervention implementation and baseline

For peer review only - http://bmjopen.bmj.com/site/about/guidelines.xhtml

characteristics for a cluster randomised controlled trial in rural Gambia. *BMJ Open*. 2018;8. doi:10.1136/bmjopen-2017-017573

44. Limaye NP, Rivas-Nieto AC, Carcamo CP, *et al.* Nuestras Historias- Designing a novel digital story intervention through participatory methods to improve maternal and child health in the Peruvian Amazon. *PLoS ONE*. 2018;13:e0205673.

45. Kumar N, Nguyen PH, Harris J, *et al.* What it takes: evidence from a nutrition- and gender-sensitive agriculture intervention in rural Zambia. *Journal of Development Effectiveness*. 2018;10:341–72.

46. Nguyen PH, Frongillo EA, Kim SS, *et al.* Information Diffusion and Social Norms Are Associated with Infant and Young Child Feeding Practices in Bangladesh. *The Journal of Nutrition*. 2019;149:2034–45.

47. Sloand E, Gebrian B. Fathers clubs to improve child health in rural Haiti. *Public Health Nurs*. 2006;23:46–51.

48. Mukuria AG, Martin SL, Egondi T, *et al.* Role of Social Support in Improving Infant Feeding Practices in Western Kenya: A Quasi-Experimental Study. *Glob Health Sci Pract*. 2016;4:55–72.

49. Winter JC, Darmstadt GL, Lee SJ, *et al.* The potential of school-based WASH programming to support children as agents of change in rural Zambian households. *BMC Public Health*. 2021;21:1–13.

50. Brahma J, Pavarala V, Belavadi V. Driving Social Change Through Forum Theatre: A Study of Jana Sanskriti in West Bengal, India. *Asia Pacific Media Educator*. 2019;29:164–77.

51. Manaseki-Holland S, Manjang B, Hemming K, *et al.* Effects on childhood infections of promoting safe and hygienic complementary-food handling practices through a community-based programme: A cluster randomised controlled trial in a rural area of The Gambia. *PLoS Medicine*. 2021;18:1–24.

For peer review only - http://bmjopen.bmj.com/site/about/guidelines.xhtml

52. Kim SS, Nguyen PH, Tran LM, *et al.* Large-Scale Social and Behavior Change Communication Interventions Have Sustained Impacts on Infant and Young Child Feeding Knowledge and Practices: Results of a 2-Year Follow-Up Study in Bangladesh. *The Journal of Nutrition*. 2018;148:1605–14.

53. Akter F, Rahman M, Pitchik HO, *et al.* Adaptation and Integration of Psychosocial Stimulation, Maternal Mental Health and Nutritional Interventions for Pregnant and Lactating Women in Rural Bangladesh. *Int J Environ Res Public Health*. 2020;17:6233.

54. Chea Nguon, Lek Dysoley, Chan Davoeung, *et al.* Art and theatre for health in rural Cambodia. *Global Bioethics*. 2018;29. doi:10.1080/11287462.2017.1411762

55. Flax VL, Negerie M, Ibrahim AU, *et al.* Integrating group counseling, cell phone messaging, and participant-generated songs and dramas into a microcredit program increases Nigerian women's adherence to international breastfeeding recommendations. *Journal of Nutrition*. 2014;144:1120–4.

56. Ghosh SK, Patil RR, Tiwari S, *et al.* A community-based health education programme for bio-environmental control of malaria through folk theatre (Kalajatha) in rural India. *Malar J*. 2006;5:123.

57. Sanghvi T, Jimerson A, Hajeebhoy N, *et al.* Tailoring Communication Strategies to Improve Infant and Young Child Feeding Practices in Different Country Settings. *Food Nutr Bull*. 2013;34:S169–80.

58. Callery JJ, Sanann N, Tripura R, *et al.* Engaging ethnic minority communities through performance and arts: health education in Cambodian forest villages. *Int Health*. 2020;13:188–95.

59. Goodman G, Dent VF. A Story Grows in Rural Uganda: Studying the Effectiveness of the Storytelling/Story-Acting (STSA) Play Intervention on Ugandan Preschoolers' School Readiness Skills. *Journal of Infant, Child & Adolescent Psychotherapy*. 2019;18:288–306.

60. Sanghvi T, Haque R, Roy S, *et al.* Achieving behaviour change at scale: Alive & Thrive's infant and young child feeding programme in Bangladesh. *Maternal & Child Nutrition*. 2016;12:141–54.

For peer review only

For peer review only - http://bmjopen.bmj.com/site/about/guidelines.xhtml

61. Kuppers P. Community arts practices: Improvising being-together. *The Community Performance Reader*. Routledge 2007.

62. Kay A. Art and community development: the role the arts have in regenerating communities. *Community Development Journal*. 2000;35:414–24. doi:10.1093/cdj/35.4.414

63. Lowe S. Creating Community: Art for Community Development. *Journal of Contemporary Ethnography*. 2000;29:357–86. doi:10.1177/089124100129023945

64. Kwan BM, Brownson RC, Glasgow RE, *et al.* Designing for Dissemination and Sustainability to Promote Equitable Impacts on Health. *Annual Review of Public Health*. 2022;43:331–53. doi:10.1146/annurev-publhealth-052220-112457

65. Coombe CM, Chandanabhumma PP, Bhardwaj P, *et al.* A Participatory, Mixed Methods Approach to Define and Measure Partnership Synergy in Long-standing Equity-focused CBPR Partnerships. *Am J Community Psychol*. 2020; doi:10.1002/ajcp.12447.

66. Jones J, Barry MM. Exploring the relationship between synergy and partnership functioning factors in health promotion partnerships. *Health Promotion International*. 2011;26:408–20. doi:10.1093/heapro/dar002

67. de Peralta AM, Smithwick J, Torres ME, *et al.* Perceptions and determinants of partnership trust in the context of Community-Based Participatory Research. *Participatory Research*. 2020;13.

68. Charles J. How Partnership Trust can Facilitate and Result from CBPR: An Assessment of Situational, Organizational, and Institutional Related Factors. *EIJ*. 2021;5. doi:10.23880/EIJ-16000186

69. Cislaghi B, Heise L. Using social norms theory for health promotion in low-income countries. *Health Promotion International*. 2019;34:616–23. doi:10.1093/heapro/day017

70. Gautam OP, Curtis V. Food Hygiene Practices of Rural Women and Microbial Risk for Children: Formative Research in Nepal. *Am J Trop Med Hyg*. 2021;105:1383–95.

For peer review only

For peer review only - http://bmjopen.bmj.com/site/about/guidelines.xhtml

746

For peer review only

For peer review only - http://bmjopen.bmj.com/site/about/guidelines.xhtml

"Co-produced, arts interventions for nurturing care (0-5 years) in Low- and Middle-Income Countries (LMICs): a realist review."

Gale et. al (2024) BMJ OPEN

Supplementary materials

Supplementary Table 1: Search Terms

Outcomes	Arts	Community	LMIC
('Child health' OR 'infant health' OR 'baby health' OR 'early years health' OR 'nurturing care' OR 'child care' OR 'child feeding' OR 'breastfeeding' OR 'hygiene' OR 'diarrhoea' OR 'sanitation' OR 'maternal health' OR 'maternal care' OR 'paternal health' OR 'paternal care' OR 'child development' OR 'child education' OR 'child wellbeing' OR 'maternal wellbeing' OR 'maternal mental health' OR 'child mental health' OR 'social cohesion' OR 'community cohesion' OR 'trust' OR 'community support' OR 'community development' OR 'social norms' OR 'health practices' OR 'care practices') AND	('Arts' OR 'drama' OR 'theatre' OR 'theater' or 'performative' OR 'creative' OR ' music' OR 'story' AND NOT 'antiretroviral') AND	('Community engagement' OR 'community mobilisation' OR 'community mobilization' OR 'community intervention' OR 'community-led' OR 'community campaign' OR 'group mobilisation' OR 'group mobilization' or 'participatory' or 'co-production' OR 'coproduction' OR 'coproduce' OR co-produce' OR 'mobilisation' OR 'mobilization') AND	('Low-income country' OR 'middle-income country' OR 'low income country' OR 'middle income country' OR 'LMIC' OR 'developing country' OR 'global south' OR Afghanistan OR 'Albania' OR 'Algeria' OR 'Angola' OR 'Antigua' .... OR 'Yemen' OR 'Zambia' OR 'Zimbabwe')

For peer review only - http://bmjopen.bmj.com/site/about/guidelines.xhtml

Supplementary Table 2: Relevant Criteria for Inclusion

Include	Exclude
Study type:<br>- Performs and reports an intervention, whether in an randomized control trial (RCT) design or in other forms (Quantitative or qualitative)<br>- Theoretical or critical articulation of community-based arts-based interventions	Not opinions, letters or editorials
Intervention is co-produced (can be led by community or public health, or equally balanced)	Conducted over 20 years ago
Focuses on early years water/food sanitation (0-5) or child development	Any language other than English, French, Spanish
Intervention takes place in LMIC	***Added after discussion:***<br>Health or nurturing care not a primary outcome, but as an unintended (if positive) outcome
Intervention is arts-based	Maternal health only as outcome
Added after discussion:<br>Intervention is arts-based, toward public health and normative behaviour change	'Community health' does not contain measure(s) using specific to under 5s
	Theory only (though useful for background section)
	Arts/story-based methodological evaluation only
	Arts-based intervention is individual therapy (no participatory aspect)

For peer review only - http://bmjopen.bmj.com/site/about/guidelines.xhtml

Supplementary Table 3: Prompt questions used for appraisal of the interventions during data synthesis

appraise **co-production** by:	appraise **contextual adaptation** by:	appraise **arts-based implementation** by:
Whose input was sought/heard at which stage?	How was the context attended to by different phases or forms of contextual learning (e.g. observations, focus groups)?	What type of dramatic arts were utilised and who delivered/facilitated their use?
Was there balanced and reciprocal participation of stakeholders, researchers, local agents and community members in design/ implementation decisions?	Was there feedback (formal or informal) between design and implementation phases?	Was the intervention flexible? in the room? Was it adaptable logistically? i.e. time and place of delivery (to improve attendance)
Were any tensions or differences in goals noted between partners? If so, were causes and/or resolutions explored?	Was there explicit incorporation of local knowledge, local infrastructure or networks, local history and social/ economic/ geographic/ political differences or inequalities?<br>Note: beyond assuming this would happen automatically through participation of local people	Were any community groups excluded from participation? Were any groups notably less engaged, untrusting or sceptical upon delivery? Did any drop-out?
Intermediary-outcome hypotheses/ mid-range theories:<br>Is there evidence these brought about partnership synergy, partnership trust, targeting of equitably representative health/care/development issues, in a manner that are broadly supported and seen as effective. And, if so, to what extent?	*Intermediary-outcome hypotheses/ mid-range theories:*<br>Is there evidence these brought about effective, targeted health/care/ development information, and sustainable transmission of such information, equitable targeting of health/care issues. And, if so, to what extent?	*Intermediary-outcome hypotheses/ mid-range theories:*<br>Is there evidence these brought about explicit or experiential knowledge of health information, shared/communal experience of health information delivery, engaging with or challenging social norms, community alignment/ social binding or synergy, affective impact of health information delivery (e.g. motivation, shame, or empowerment). And, if so, to what extent?

For peer review only - http://bmjopen.bmj.com/site/about/guidelines.xhtml

Supplementary Table 4: Search results breakdown

DB search engine	Database(s)	Results
EBSCO	Anthropology Plus, ASSIA, CINAHL Plus, EconLit, Education Abstracts (H.W. Wilson), Ergonomics Abstracts, ERIC, Humanities Abstracts (H.W. Wilson), Humanities Full Text (H.W. Wilson)	516
OVID	APA PsycArticles, Embase, HMIC, CAB Abstracts and Global Health, Ovid MEDLINE(R) and In-Process, APA PsycInfo, Social Policy and Practice, BooksOvid, journalsOVID	211
Proquest	Arts & Humanities Database, Biological Science Database,East & South Asia Database, East Europe, Central Europe Database, Education Collection, Environmental Science Database, Health & Medical Collection, India Database, International Bibliography of the Social Sciences (IBSS), Middle East & Africa Database, Nursing & Allied Health Database, Political Science Database, Psychology Database, Public Health Database, Religion Database, Social Science Database, Sociology Collection	568
Scopus		266
Web of Science		42
Child Adolescence and Education Studies		38
	Total	1641
	Duplicates removed	940
	Titles/abstracts screening	175

For peer review only

For peer review only - http://bmjopen.bmj.com/site/about/guidelines.xhtml

Supplementary Figure 1: Search strategy process overview

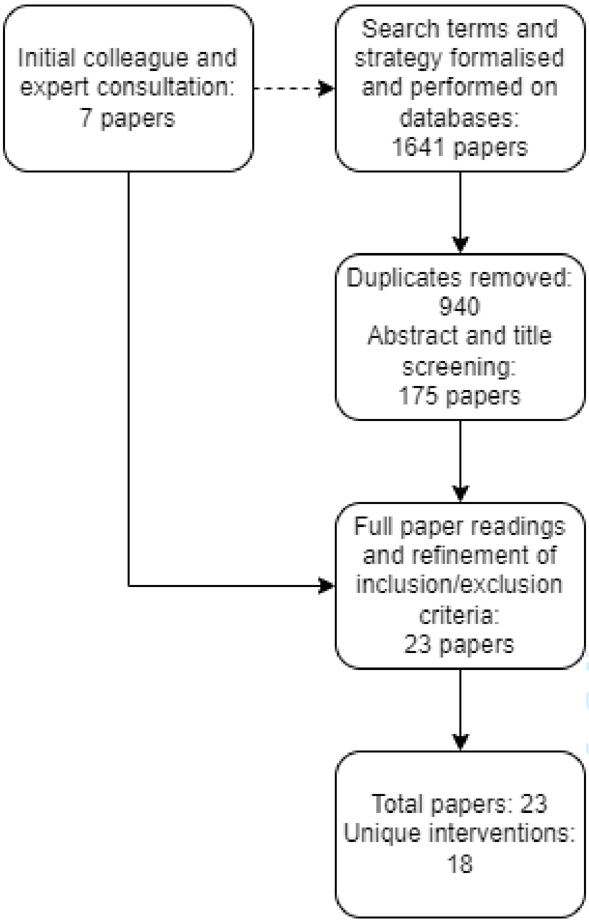

For peer review only - http://bmjopen.bmj.com/site/about/guidelines.xhtml

Supplementary Table 5: Summary of included papers

Author (Date)	Inter-vention Date	Title (s)	Country of intervention	Country of first author	Primary Discipline	Evaluation methods used	Intended Nurturing Care/Health Outcomes	Main Findings
Akter et al. (2020)	2017	Adaptation and Integration of Psychosocial Stimulation, Maternal Mental Health and Nutritional Interventions for Pregnant and Lactating Women in Rural Bangladesh	Bangladesh	Bangladesh	Public Health (Infectious Disease)	Qualitative evaluation (interviews) of a multi-phase adaptation process for an intervention.	Early childhood development	Most participants reported willingness to attend the sessions if extended for 1 year, and recommended additional visual cues and interactive role-play activities to make the sessions more engaging. Participants and community health workers found it difficult to understand the concept of "unhealthy thoughts" in the curriculum. This component was then revised to include a simplified behavior-focused story. Community health workers reported difficulty balancing the required content of the integrated curriculum but were able to manage after the contents were reduced. The revised intervention is likely feasible to deliver to a group of pregnant and lactating mothers in a low-resource setting.
Al-Delaimy et al. (2014)	2012-2013	Developing and evaluating health education learning package (HELP) to control soil-transmitted helminth infections among Orang Asli children in Malaysia	Malaysia	Iraq	Public health (Health Education & Community Medicine)	Mixed-methods approach: literature review, community discussions & impact evaluation of an open-label controlled intervention trial	Knowledge about soil-transmitted helminth infections	The developed package consists of a half day workshop for teachers, a teacher's guide book to STH infections, posters, a comic book, a music video, a puppet show, drawing activities and an aid kit. The package was well-received with effective contributions being made by teachers, children and their parents. The incidence rates of hookworm infection at different assessment points were significantly lower among children in the intervention school compared to those in the control school. Similarly, the intensity of trichuriasis, ascariasis and hookworm infections were found to be significantly lower among children in the HELP group compared to those in the control group ($P < 0.05$). Moreover, the package significantly improved the knowledge, attitude and practices (KAP) of Orang Asli people and the knowledge of teachers towards STH infections.

For peer review only - http://bmjopen.bmj.com/site/about/guidelines.xhtml

Callery et al. (2020)	2018	Engaging ethnic minority communities through performance and arts: health education in Cambodian forest villages	Cambodia	Thailand	Tropical Medicine	Qualitative evaluation (interviews)	Health information retention; assessing contextual-appropriateness	Respondents were able to recall the key health messages about malaria, antenatal care and infant vaccination. They also showed good awareness of malaria transmission and prevention and described how they enjoyed the events and appreciated the efforts of the project team. In isolated communities in Cambodia, a health education programme harnessing performance and arts engaged the whole community and its messages were readily recalled and prompted reflection.
Flax et al. (2014)	2011-2012	Integrating Group Counseling, Cell Phone Messaging, and Participant-Generated Songs and Dramas into a Microcredit Program Increases Nigerian Women's Adherence to International Breastfeeding Recommendations	Nigeria	US	Public Health Nutrition	Cluster randomised control trial evaluation of an intervention, interviews, and survey	Early breastfeeding initiation; exclusive breastfeeding	Among the clients who completed the final survey (n = 390), the odds of exclusive breastfeeding to 6 mo (OR: 2.4; 95% CI: 1.4, 4.0) and timely breastfeeding initiation (OR: 2.6; 95% CI: 1.6, 4.1) were increased in the intervention vs. control arm. Delayed introduction of water explained most of the increase in exclusive breastfeeding among clients receiving the intervention. In conclusion, a breastfeeding promotion intervention integrated into microcredit increased the likelihood that women adopted recommended breastfeeding practices. This intervention could be scaled up in Nigeria, where local organizations provide microcredit to >500,000 clients. Furthermore, the intervention could be adopted more widely given that >150 million women, many of childbearing age, are involved in microfinance globally.
Goodman and Dent (2019)	2014	A Story Grows in Rural Uganda: Studying the Effectiveness of the Storytelling/Story-Acting (STSA) Play Intervention on Ugandan Preschoolers' School Readiness Skills	Uganda	US	Psychotherapy	Randomised control trial and qualitative evaluation (caregiver interviews)	School readiness skills	Overall, participants benefited significantly from a story-reading activity with or without STSA. When examining both groups together (N = 121 post-intervention), school readiness skills significantly improved. Caregiver variables also predicted these three child outcome variables at baseline, suggesting that caregivers play a significant role in the development of their children's school readiness skills.

For peer review only - http://bmjopen.bmj.com/site/about/guidelines.xhtml

Gautam et al. (2017) Gautam and Curtis (2021)	2012-2013	Trial of a Novel Intervention to Improve Multiple Food Hygiene Behaviors in Nepal<br>Food Hygiene Practices of Rural Women and Microbial Risk for Children: Formative Research in Nepal	Nepal	UK	Tropical Medicine	Cluster randomised control trial evaluation of an intervention; Process evaluation and mixed-method analysis of an intervention adaptation process	Food hygiene behaviors	The five targeted food hygiene behaviors were rare at baseline (composite performance of all five behaviors in intervention 1%[standard deviation (SD)= 2%] and in control groups 2%[SD= 2%]). Six weeks after the intervention, the target behaviors were more common in the intervention than in the control group (43%[SD= 14%] versus 2%[SD= 2%], P= 0.02) during follow-up. The intervention appeared to be equally effective in improving all five behaviors in all intervention clusters. This study shows that a theory-driven, systematic approach employing emotional motivators and modifying behavior settings was capable of substantially improving multiple food hygiene behaviors in Nepal.
Ghosh et al. (2006)	2001	A community-based health education programme for bio-environmental control of malaria through folk theatre (Kalajatha) in rural India	India	India	Epidemiology (Malaria research)	Impact assessment (interviews assessing knowledge and behaviour) comparing intervention with control villages	Control of malaria	The exposed respondents had significant increase in knowledge and change in attitude about malaria and its control strategies, especially on bio-environmental measures (p < 0.001). They could easily associate clean water with anopheline breeding and the role of larvivorous fish in malaria control. In 2002, the local community actively co-operated and participated in releasing larvivorous fish, which subsequently resulted in a noteworthy reduction of malaria cases. Immediate behavioural changes, especially maintenance of general sanitation and hygiene did not improve as much as expected.
Kim et al. (2018)	2010-2014	Large-Scale Social and Behavior Change Communication Interventions Have Sustained Impacts on Infant and Young Child Feeding Knowledge and Practices: Results of a 2-Year Follow-Up Study in Bangladesh	Bangladesh	US	Nutrition	Cluster randomized nonblinded impact evaluation and comparison between more and less intensive intervention packages, via cross-sectional household survey	Infant and young child feeding knowledge and practices	In intensive areas, exposure to IPC decreased slightly between endline and follow-up (88.9% to 77.2%); exposure to CM activities decreased significantly (29.3% to 3.6%); and MM exposure was mostly unchanged (28.1–69.1% across 7 TV spots). Exposure to interventions did not expand in nonintensive areas. Most IYCF indicators in intensive areas declined from endline to follow-up, but remained higher than at baseline. Large differential improvements of 12–17 percentage points in intensive, compared with nonintensive areas, between baseline and follow-up remained for early initiation of and exclusive breastfeeding, timely introduction of foods, and consumption of iron-rich foods. Differential impact in breastfeeding knowledge remained between baseline

For peer review only - http://bmjopen.bmj.com/site/about/guidelines.xhtml

								and follow-up; complementary feeding knowledge increased similarly in both groups.
Kumar et al. (2018)	2011-2015	What it takes: evidence from a nutrition- and gender-sensitive agriculture intervention in rural Zambia	Zambia	USA	Food policy	Cluster-randomised, controlled, non-blinded, impact evaluation (cross-sectional household survey and child stunting measurements)	Child undernutrition (via women's empowerment, IYCF knowledge and practices and child anthropometry)	The RAIN project had positive effects on women's empowerment, IYCF knowledge, child morbidity and weight-for-height z-scores, but had little impacts on IYCF practices, and no impact on stunting. Strengthening programme implementation and fostering higher participation rates could support greater impacts on child nutrition outcomes.
Limaye et al. (2018)	2017	Nuestras Historias-Designing a novel digital story intervention through participatory methods to improve maternal and child health in the Peruvian Amazon	Peru	USA	Pediatrics	sequential exploratory mixed-method study design (qualitative: process of digital story curriculum creation; quantitative: acceptability)	Maternal mortality rates	According to the PhotoVoice workshops, pregnancy-related problems included: lack of partner support, domestic violence, early pregnancies, difficulty attending prenatal appointments, and complications during pregnancy and delivery. Over 30 stories on these themes were recorded. Seven were selected based on clarity, thematic relevance, and narrative quality and were edited by a professional filmmaker. The acceptability survey showed that local participants found the digital stories novel (M = 89.4%, F = 83.3%), relatable (M = 89.4%, F = 93.2%), educational (M = 91.5%, F = 93.3%) and shareable (M = 100%, F = 100%). Over 90% of respondents rated the digital stories as "Excellent" or "Good", found the videos "Useful" and considered them "Relevant" to their communities.

For peer review only - http://bmjopen.bmj.com/site/about/guidelines.xhtml

Manjang, et al. (2018) Manaseki-Holland et al. (2021)	2014-2015	Promoting hygienic weaning food handling practices through a community-based programme: intervention implementation and baseline characteristics for a cluster randomised controlled trial in rural Gambia.<br>Effects on childhood infections of promoting safe and hygienic complementary-food handling practices through a community-based programme: A cluster randomised controlled trial in a rural area of The Gambia	The Gambia	The Gambia/ UK	Public Health/ Paediatrics	Cluster randomised control trial	Complementary-food contamination and disease rates	For the primary outcome, the rate was 662/4,351(incidence rate [IR] = 0.15) in control villages versus 2,861/4,378 (IR = 0.65) in intervention villages (adjusted incidence rate ratio [aIRR] = 4.44, 95% CI 3.62–5.44, p < 0.001), and at 32 months the aIRR was 1.17 (95% CI 1.07–1.29, p = 0.001). We found that low-cost and culturally embedded behaviour change interventions were acceptable to communities and led to short- and long-term improvements in complementary-food safety and hygiene practices, and reported diarrhoea and acute respiratory tract infections.
McMichael & Robinson (2016)	2008-2012	Drivers of sustained hygiene behaviour change: A case study from mid-western Nepal	Nepal	Australia	Geography	Qualitative case-study evaluation (interviews, group discussions, drawings and household observation)	Water and sanitation-related diseases	Findings indicate the intervention has supported development of new norms around hygiene behaviours. Key drivers of sustained hygiene behaviour were habit formation, emotional drivers (e.g. disgust, affiliation), and collective action and civic pride; key constraints included water scarcity and socio-economic disadvantage. Identifying and responding to the drivers and constraints of hygiene behaviour change in specific contexts is critical to sustained behaviour change and population health impact.

For peer review only - http://bmjopen.bmj.com/site/about/guidelines.xhtml

Nguon et al. (2018)	2016-17	Art and theatre for health in rural Cambodia	Cambodia	Cambodia	Epidemiology (Parasitology)	Description & Reflection on lessons learnt	Dissemination of key health messages and supplementing existing engagement activities conducted by local authorities	We learnt the following lessons from our experience in Cambodia: involving local people including children from the beginning of the project and throughout the process is important; messages should be kept simple; it is necessary to take into consideration practical issues such as location and timing of the activities; and that the project should offer something unique to communities.
Olaide (2010)	Not stated	Maternal mortality and theatre intervention: A case study of Oluyole	Nigeria	Nigeria	Performing Arts	Descriptive case study evaluation	Maternal mortality rates	The paper discusses the practice of theatre for development as an information and enter-educative medium to the people most of whom are western educationally disadvantaged.

For peer review only

For peer review only - http://bmjopen.bmj.com/site/about/guidelines.xhtml

Sanghvi et al. (2013)<br>Sanghvi et al. (2016)<br>Nguyen et al. (2019)	2010-2016	Tailoring communication strategies to improve infant and young child feeding practices in different country settings;<br>Achieving behaviour change at scale: Alive & Thrive's infant and young child feeding programme in Bangladesh;<br>Information Diffusion and Social Norms Are Associated with Infant and Young Child Feeding Practices in Bangladesh	Bangladesh Ethiopia & Vietnam	USA	International Development	Cross country comparison of intervention design, adaptation phases and outcomes;<br>Process description of multi-method intervention design and scale-up phases and evaluation via preliminary comparisons of intensive vs non-intensive treatment groups;<br>Household surveys - analysis of paths from intervention exposure to IYCF practices through networks, diffusion, and norms (multiple regression analysis)	Exclusive breastfeeding and complementary feeding practices	Key messages: Well-designed and well-implemented large-scale interventions that combine interpersonal counselling, community mobilization, advocacy, mass communication and strategic use of data have great potential to improve IYCF practices rapidly.<br>Formative research and ongoing studies are essential to tailor strategies to the local context and to the perspectives of mothers, family members, influential community members and policymakers. Continued use of data to adjust programme elements is also central to the process.<br>Scale-up can be facilitated through strategic selection of partners with existing community-based platforms and through mass media, where a high proportion of the target audience can be reached through communication channels such as broadcast media.<br>Sustaining the impacts will involve commitments from government and capacity building. The next step for capacity building would involve understanding barriers and constraints and then coming up with appropriate strategies to address them. One of the limitations we experienced was rapid transition of staff in key positions of implementing agencies, in government leadership, donors and other stakeholders. There was a need for continued advocacy, orientation and teaching related to strategic programme design, behaviour change, effective implementation and use of data.

For peer review only - http://bmjopen.bmj.com/site/about/guidelines.xhtml

Sloand and Gebrian (2006)	1994-2005	Fathers Clubs to Improve Child Health in Rural Haiti	Haiti	US	Nursing	Literature Review	Child and family health	Public health practitioners continually face grave challenges when addressing the health care needs in less developed countries. One such case is Haiti, where crushing poverty, poor infrastructure, a failing economy, natural disasters, and chaotic sociopolitical conditions compound these challenges. Public health practitioners, including nurses, must seek creative, culturally-appropriate, low technologic approaches to improve the health of the children and families in the remote villages of Haiti. The institution and support of village fathers clubs is one approach that has been ongoing since 1994. Fathers meet together on a regular basis for health education, support, and community building activities. The curriculum is health-based and facilitated by nurses, with participation by young and old men alike. Participants and organizers believe that family and child health is improved as a result of the groups.
Thuita et al. (2015) Mukuria et al. (2016)	2010-12	Engaging fathers and grandmothers to improve maternal and child dietary practices: planning a community-based study in Western Kenya<br>Role of Social Support in Improving Infant Feeding Practices in Western Kenya: A Quasi-Experimental Study	Kenya	Kenya/ USA	Public Health	Quasi-experimental, non-equivalent comparison group design with pre- and post-testing (surveys, formative assessment and process evaluation);<br>Quasi-experimental, multi-method, multi-phase intervention description and evaluation (survey)	Maternal and child dietary practices; Complementary feeding practices	We surveyed 554 people at baseline (258 mothers, 165 grandmothers, and 131 fathers) and 509 participants at endline. The percentage of mothers who reported receiving 5 or more social support actions (of a possible 12) ranged from 58% to 66% at baseline in the 3 groups. By endline, the percentage had increased by 25.8 percentage points (P=.002) and 32.7 percentage points (P=.001) more in the father and the grandmother intervention group, respectively, than in the comparison group. As the number of social support actions increased in the 3 groups, the likelihood of a mother reporting that she had fed her infant the minimum number of meals in the past 24 hours also increased between baseline and endline (odds ratio [OR], 1.14; confidence interval [CI], 1.00 to 1.30; P=.047). When taking into account the interaction effects of intervention area and increasing social support over time, we found a significant association in the grandmother intervention area on dietary diversity (OR, 1.19; CI, 1.01 to 1.40; P=.04). No significant effects were found on minimum acceptable diet.

For peer review only - http://bmjopen.bmj.com/site/about/guidelines.xhtml

Winter et al. (2021)	2017-2018	The potential of school-based WASH programming to support children as agents of change in rural Zambian households	Zambia	USA	Civil and Environmental Engineering	Quasi-experimental, prospective cohort intervention impact evaluation (open and closed surveys)	Sharing of WASH-related messages (washing hands and using the latrine) with caregivers	Student knowledge increased significantly, but primarily among students in grade 1. Overall rates of students reporting that they shared messages from the curriculum with their caregivers rose from 7 to 23% ($p < 0.001$). Students in grade 4 were 5.2 times as likely as those in grade 1 to report sharing a WASH-related message with their caregivers (ARR = 5.2, 95% C.I. = (2.3, 8.9); $p < 0.001$). Although we measured only modest levels of student dissemination of WASH UP! messages from the school to the home, students in grade 4 showed significantly more promise as agents of change than those in grade 1.

For peer review only - http://bmjopen.bmj.com/site/about/guidelines.xhtml

Supplementary Table 6: Summary of analysis and review findings

Author (Date)	Co-production	Adaptation to context	Use of dramatic arts	What works, for whom, & in what circumstances? Was it sustainable?	Scalability/ Transferability
Akter et al. (2020)	Researcher-led process, with feedback from participants, community members and community health workers.	Three existing interventions were integrated and culturally adapted via researcher knowledge and situation analysis (direct observation).<br><br>Two models of delivery were piloted - one in 'courtyard groups' and the other in individual home visits. Used local school infrastructure for delivery.	Arts-based approaches were not part of the original intervention but added after community feedback. Health messages were conveyed through use of visual cues, interactive role play and storytelling.<br><br>Delivered to communities by community health workers.	Participants reported willingness to attend the sessions if they were extended for 1 year. Changes recommended included additional visual cues and interactive role-play activities to make the sessions more engaging, a simplified behaviour-focused story (rather than the concept of 'unhealthy thoughts'), and to reduce the amount of content.<br><br>The study did not collect long-term data.	Small scale limits generalisability.<br><br>A systematic participatory approach was taken in intervention design, and a revised intervention was proposed which was likely feasible to deliver to a group of pregnant and lactating mothers in a low-resource setting.<br><br>Group sessions were as effective as combined ones and therefore potentially more cost-effective and scalable.
Al-Delaimy et al. (2014)	Researcher-led process, with engagement of a range of academic disciplines (e.g. public health, parasitology, education), local public health and community development officials, as well as residents, community members and service providers.<br><br>Limited opportunities for feedback during or after delivery.	Local adaptation of health education messages, via literature review and direct observation (household survey) by researchers of the Orang Asli communities, responding to a history of 'failed' interventions in the region.	Posters/comic books, nursery rhymes and songs (lyrics on posters and songs on YouTube and school computers) and a puppet show were used alongside more traditional learning materials.<br><br>Delivered to child-participants by teachers, who were trained in relevant health content.	Knowledge and behaviour improved (with variable onset, longevity and extent), and school infrastructure was inclusive of range of children.<br><br>No long-term outcomes, beyond 6 months (where control group had lower infections, but below statistical significance threshold). Long-term funding unclear.	The intervention used teachers making it scalable, but how willing or able teachers are to help will vary between individuals and places. The arts elements depended on availability of school materials e.g. computers, so may not be reproducible in all contexts.

For peer review only - http://bmjopen.bmj.com/site/about/guidelines.xhtml

Callery et al. (2020)	Researcher-led and stakeholder-funded. Involvement of a range of stakeholders at different levels, including Deputy District Governor, commune and village leaders, primary school principals, health centre chiefs, the police chief and village malaria workers.	A existing intervention was adapted to local context via a large stakeholder orientation meeting (n=~50) at a community health centre, where the activities, target villages and topics for community engagement were discussed.<br><br>This was followed by additional local, informal adaptation in each village, through meeting with commune and village leaders, the principal of the local school and village malaria workers to discuss with them the activities and inviting the students to participate.<br><br>TV was used to promote the events and celebrate.	Intervention was delivered by a community engagement team and members of a professional theatre group experienced in community/ village drama.<br><br>Participatory art and theatre workshops were delivered to school children to prepare for a village performance, which included wider members of the community. The workshops with schoolchildren were facilitated by teachers, with parental consent.<br><br>The performance included games, singing and dancing, health quizzes, a fashion show (showcasing traditional costumes, dance and musical instruments) and a comedy sketch about malaria. The closing event involved speeches, competitions, performances from professional singers, local traditional dances and an amateur singing competition.	Qualitative interviews suggested that knowledge of health messages had improved, with general recall of messages but not all details. Some participants shared the messages with non-participants. Involvement of children was noted to boost investment of families and teachers (and to require mastery of health messages for the performance).<br><br>Ethnic minority groups were specifically engaged and asked to be special guests at arts events in other villages. Showcasing elements helped the participants and the audience to gain a sense of ownership of the performances and this element was particularly popular among the local Kaviet and Lao populations.<br><br>No long term outcomes reported. Unclear whether further funding would be required or available.	This intervention was an adapted reproduction of preceding interventions, so can be considered reproducible. No immediate barriers to scaling were noted.

For peer review only - http://bmjopen.bmj.com/site/about/guidelines.xhtml

Flax et al. (2014)	Researchers partnered with a US NGO (Partners for Development) and 4 local, Nigerian community-based organizations.<br><br>Community members were recruited into microcredit groups of 5-7 friends, neighbours, or relatives who guaranteed each other's loans.	The intervention was delivered within an established social/economic grouping - women's micro-credit groups - that met monthly.<br><br>It was unclear whether there was any local adaptation or feedback process to the intervention.	Small groups of residents living near each other (in the microcredit groups) were asked to perform one of the key messages in a song or dramatic form.	Knowledge and behaviour improved, and intervention groups did breastfeed more at 3 and 6 months.<br><br>No longer term outcomes documented.	The microcredit aspect of this intervention may be difficult without American developmental organisation support. Could also cause social disruption instead of solidarity if poorly managed. Other aspects are low-cost and use local organisations.
Goodman and Dent (2019)	Researcher-led process, whereby researchers identified the problem and designed the intervention then, using translators, trained the local project coordinators to deliver the intervention.	The team ensured that the intervention was low cost and used local school infrastructure for delivery.	Storytelling and story-acting were used in the intervention. The story development was entirely child-led and 'spontaneous', integrated into play.	No difference overall between intervention and control group (who did drawing instead of story-telling). Some evidence that lower-income students and girls benefitted more.<br><br>No long-term outcomes reported. It was unclear whether further funding would be required or available.	Storytelling is low-resource and but limited evidence of effectiveness. The use of local schools for delivery would be scalable.

For peer review only - http://bmjopen.bmj.com/site/about/guidelines.xhtml

Gautam et al. (2017) Gautam and Curtis (2021)	Researcher-led process, drawing on literature and theoretical models of behaviour change (Behaviour-Centered Design), but also engaging a range of stakeholders and local communities. Government and non-governmental organizations' advised that the intervention adapted from preceding interventions in Mali and India would be replicable and scalable in Nepal. A local creative team was assembled to design the intervention. Local women with a similar profile to Nepal's ubiquitous Female Community Health Volunteers (FCHVs) an intervention package was designed and delivered, with a local creative team with expertise in marketing, design, innovation, program development, and behaviour change.	The intervention design was informed by formative research conducted by 4 local research field data collectors and five local women from different castes/ethnic groups using handheld cameras, and analysed by the research team. Following this group discussions with community members and health officials.<br><br>Several pilots took place for feedback from those delivering the intervention and the communities. Final feedback was from government officials and NGO stakeholders.	Local rallies, folk songs, games, rewards, storytelling, drama, cookery demonstrations and "kitchen makeover parties" were explicitly linked to the motivational drivers of behaviour identified in the guiding programme theory (Affiliation, Disgust, and Status).	The intervention changed five key behaviours observed in formative research to improve the microbiological environment. Women who engaged undertook public pledging and were celebrated with a public display of photos. The intervention was effective in differing socio-economic settings, although it is unclear if there were inequalities within settings in the women who did and did not participate.<br><br>No long term outcomes observed.	That the intervention was equally effective across targeted behaviours and across clusters is a positive indicator that the improvements are reproducible. Health workers found that it was feasible to deliver the intervention on top of their existing workload, suggesting potential scalability.

For peer review only - http://bmjopen.bmj.com/site/about/guidelines.xhtml

Ghosh et al. (2006)	Led by an intersectoral committee (involving ten governmental and non-governmental organisations), and delivered by local agents. Funded by the government, whose ministers provided visibility for the programme. Religious leaders contributed by offering free accommodation and hospitality for the period of one month as a token of solidarity in the fight against malaria. Local press and radio helped in wider dissemination of health education messages.<br><br>Local and regional organisations supported the intervention, e.g. the education department deputed five teachers and the Child and Women's Welfare Department deputed ten Anganwadi workers for one month.	Informal adaptation through a two-day visit from the intervention team prior to the final event.<br><br>Unclear if there were opportunities for feedback once the intervention had started.	Local educators, artists, religious leaders and community members collaborated to deliver the intervention.<br><br>Kalajatha is a popular, traditional art form of folk theatre depicting various life processes of a local socio-cultural setting . A local scriptwriter wrote 8 songs, two dramas and 4 rupakas (musical dramas) based on various aspects of malaria. These were then translated into skits using local dialects, musical styles and theatre traditions.<br><br>Thirty local artists (15 males and 15 females) from different occupational background were selected. Female artists were involved in the team, which resulted in better engagement from the women in the community.	Immediate behaviour changes were not evident but knowledge and attitudes of participants improved.<br><br>No long term outcomes were directly observed, but in a subsequent malaria prevention study via biocontrol in the area the year after this intervention, a dramatic improvement in malaria incidence was observed and in the study area people still refer to the Kalajatha events and the messages delivered to them, suggesting long term knowledge retention.	This intervention was implemented through and with existing local and regional organisations. The theatre performances themselves should not be highly expensive and require few materials.

For peer review only

Kim et al. (2018)	Donor-led initiative, working with researchers (from outside the country). The focus of the intervention shifted somewhat with new donors, who scaled up and adapted the programme. This is a top-down change (or feedback process).<br><br>The community-based infection and prevention control work was delivered by a international development organisation based in Bangladesh. Local health workers/volunteers/artists and regional/local infrastructures and organisations were used to deliver Infant and Young Child Feeding (IYCF) messages in regular meetings, community video and theatre shows, as well as radio and TV.	Broad consideration of context in intervention design (media, situation analysis, communications) concept testing, leading to creative development of prototype materials, (6) material pretests followed by revisions<br><br>The campaign implementation was subjected to ongoing monitoring, which was reported to lead to early and mid-course adjustments. However, evidence of bottom up changes/feedback incorporation in substantive content, or form of its delivery were not explicit.	Arts were only one component of a comprehensive programme of health promotion. Community video and theatre shows focused on infant and young child feeding. TV spots were broadcast nationally and regionally with intervention relevant messages.	This study offers evidence of decreased yet sustained impacts on IYCF practices even under modifications, suggesting program drift. Continued exposure infection prevention and control messages and sustained impacts on infant and young child feeding practices indicate lasting benefits from these interventions, as they underwent major scale-up and adaptations after termination of initial external donor support. At 2 y after endline, intervention exposure had decreased, but home visits by frontline workers remained moderately high in the intensive areas and significantly higher compared with the non intensive areas.<br><br>The programme is reliant on significant funding from external (non-governmental and internation) organisations, so sustainability is dependent on this continuing.	This project involved lots of government and non-government organisations. The practices of community arts-based health messaging is reproducible given low cost when working alongside existing organisations and with existing infrastructures.<br><br>In other contexts, where perhaps there was not be a broad political consensus on health priorities, expensive aspects of the current intervention may not be replicable, such as the TV ads. Reproducing this kind of comprehensive project at scale is challenging and demands significant collaboration.

For peer review only - http://bmjopen.bmj.com/site/about/guidelines.xhtml

Kumar et al. (2018)	International humanitarian organisation established partnerships with relevant government line ministries and with local implementing NGOs who were to deliver programme content across the different project components. Evaluation was conducted by International Food Policy Research Institute.	Used existing community health volunteers and women's groups, and existing government IYCF guidelines and materials to implement a gender-sensitive homestead food production programme with nutrition behaviour change communication.<br><br>Contextual issues were discussed in the paper and and initial gender analysis informed the intervention, but it is unclear whether there was any further adaptation informed by stakeholders.	Drama-based approaches were part of broader range of activities, including agricultural interventions (home gardening, providing seeds and equipment), and behaviour change components (group activities, radio messaging, posters, brochures) and women's empowerment (supply of fuel-saving stoves and fuel trees as labour-saving technologies). Community sensitisations were undertaken with assistance from drama groups, particularly around gender equality and its importance for improved nutrition. It is unclear whether these were participatory in delivery.	Positive effects were observed on women's empowerment, IYCF knowledge, child morbidity and weight-for-height z-scores, but little impact was seen on IYCF practices, and no impact on stunting. Areas that could be strengthened were programme implementation and fostering higher participation rates.<br><br>Long term outcomes were not observed.	The project was initially funded by international research organisations. It used local infrastructure and at times altered it, or offered incentives for participation (e.g. bicycles and/or agricultural inputs). The processes of adaptation to context (in design and delivery) are not very explicit.
Limaye et al. (2018)	Design of a digital story curriculum was participatory: the researcher ran three community-based PhotoVoice workshops with Community Health Workers and women who were invited by the CHWs; then they were involved in participatory digital storytelling activities. Stories were selected by the research team, who worked with a professional filmmaker and a professional midwife to develop these stories into a final digital story curriculum, where each story was combined with question-based teaching prompts.	The programme worked with existing community health workers, and the curriculum was iteratively developed with a variety of stakeholders.<br><br>There were limited opportunities later in the project for local storytellers to influence the final product (due to limitations of needing to process the digital stories in a city).	Photography (photo-voice) and community-based digital storytelling activities were used to inform the development of the curriculum. Participants were encouraged to think of personal stories and recorded themselves on mobile devices narrating their stories. Then participants did a storyboarding activity, in which they broke down the components of their story, and determined what types of photos would fit with each component of the story.	The digital story curriculum was highly acceptable to the target population, both men and women. They were considered novel, relatable, educational, and shareable.<br><br>Long term outcomes were not observed.	Solar-powered mobile phones with cameras are becoming cheaper and so the participatory process for designing a curriculum in this way is perhaps likely more replicable over time. Reproducibility was not investigated directly, but if able to utilise existing networks of local health workers and secure funding, there are no obvious issues.

For peer review only - http://bmjopen.bmj.com/site/about/guidelines.xhtml

Manjang, et al. (2018) Manaseki-Holland et al. (2021)	Local researcher-led, with wide consultation with health promotion agencies who were represented on a Local Scientific Advisory Committee in the Gambia (MOH, Unicef, WHO, University of the Gambia, National Nutrition Agency (NANA) and the MRC Gambia).<br><br>Locally, traditional communicators, community leaders and traditional birth attendants or other experienced women/ grandmothers were engaged. Women's achievements were celebrated individually (as MaaChampions) and communally (through villages achieving MaaChampion status).	The intervention was based on theoretical models of behaviour change and findings from previous similar intervention studies (in Nepal and India) and were adapted to the local context, based on formative research and consultation with stakeholders. The intervention material was translated into the three local languages (Mandinka, Wolof and Fula), field-tested with mothers and villagers and piloted iteratively by the intervention team.<br><br>TV was used to promote the events.<br><br>Women's networks were used to convey the messages 'onward' from the intervention, finding more volunteers. These women visited the families between the visits from the wider team to help establish the practices within the cultural norms of the community.	Performing arts (using culturally ingrained styles of drama and songs), animation, and stories.<br><br>Delivery team included traditional communicators (male and female, who use traditional African drumming, singing and acting), public health officers, a driver and a trained female volunteer, usually a traditional birth attendant (TBA).<br><br>Villagers reported fond memories of a 'joyous' programme with songs and activities of traditional performing artists, and they 'treasured' the achievements of MaaChampions in their family and community.	After intervention, behaviours improved (higher soap use in domestic kitchens and latrines) and there was a significant reduction in reported diarrhoea episodes and hospital admissions in the rainy season. No information was available about any inequalities in its effect.<br><br>Improved knowledge of the key behaviour messages was sustained at 6 months.<br><br>Qualitative data at 32 months suggested that mothers in intervention villages felt it their duty to inform mothers about complementary-food safety and hygiene practices when visiting other villages.	Cost-minimising tips were discussed toward replicability. Local structures and personnel used, with a low cost intervention.<br><br>This study builds on a smaller cluster RCTs of similar interventions in Nepal and India. This suggests the potential for reproducibility across different LMICs, and scope for scaling.<br><br>This low-cost intervention was not only acceptable to the mothers and the communities at large but was, at least in part, self-sustaining since new mothers reported practicing the behaviours at 32 months.

For peer review only - http://bmjopen.bmj.com/site/about/guidelines.xhtml

McMichael & Robinson (2016)	Researcher-led, in partnership with Australian and Nepal Red Cross. Two non-local researchers led the data collection with the assistance of a Nepali bilingual research assistant and two Nepali interpreters (one male, one female). Local residents were involved in some elements, e.g. children in participatory hygiene education and activities, group/committee formation (e.g. junior/youth Red Cross circles, women's groups, Water User Committees) and employment of local Village Health Motivators to conduct community-based hygiene education and home visits. Significant changes were made as a result of community feedback: for example, the original intervention employed Community-Led Total Sanitation triggering tools to create demand for latrines and improved hygiene (e.g. "walk of shame", flagging faeces) which were found to be 'difficult' and removed when all used the toilets. Positive motivational drivers were reported to be more effective.	Feedback was sought in focus group discussions and in depth-interviews. These were used to evaluate intervention impact. Ongoing program monitoring was noted but is unclear how this may have impacted adaptation beyond the informal adaptation through participatory activities.<br><br>Arts-based elements used as part of a broader intervention programme addressing different dimensions of the socio-economic and public health context: including provision of partially subsidised hardware; group/committee formation; hygiene and sanitation education; distribution of WASH information and education materials; employment of local Village Health Motivators to conduct community-based hygiene education and home visits; peer-led WASH awareness raising in schools/community; hygiene and sanitation campaigns; faeces calculation and fining of open defecation; and establishment of community funds that provide small loans for toilet construction.	Children and community members were engaged through the arts to raise awareness of WASH - through street drama and song.	Qualitative evidence showed changes in hygiene knowledge, behaviour and development of new social norms around hygiene. There was some anecdotal evidence of improvements in health.<br><br>Contextual limitations included the prohibitive cost of further latrine construction, and an inconsistent water supply which limited flushing and handwashing practice.<br><br>This paper presents qualitative reports on the interventions impact two years after beginning. The project's impact appears to have been supported by the diverse and intensive behaviour change activities that were repeated over a long period time by peers as well as community leaders.	Small-scale and intensive nature limits the applicability of these findings to large-scale WASH interventions.<br><br>Latrine construction could not persist due to the cost.

For peer review only - http://bmjopen.bmj.com/site/about/guidelines.xhtml

Nguon et al. (2018)	Led by local researchers, in partnership with the provincial health authorities, who selected and trained a local drama group to deliver the intervention based on a competitive process.<br><br>Community members were involved through attending the workshops, which led to the local tailoring of content for the public performances, and in quizzes/competitions.<br><br>High trust and synergy was reported between performers and the community.	This project (a pilot science-arts initiative) supplemented existing engagement activities conducted by local authorities and a community engagement team supporting a malaria elimination study (clinicaltrials.gov NCT01872702).<br><br>There was no formative research or formal adaptation process, but workshops were held in each village by the drama team, to adapt the performance using real stories of locals' experiences with malaria.	In each village there was a two and a half-day workshop led by a team consisting of local drama groups and health workers. There was a 'memorable' public performance on the third evening, incorporating local legends and real stories from the community. Community members, including children, were engaged using comedy, drama, drawing, singing competitions and quizzes about the intervention messages with prizes offered.	The drama storylines and health messages were remembered across the groups, and there was media interest in the project.<br><br>Attendance was lower proportionally in larger villages.<br><br>No long-term outcomes reported.	Intervention attracted large audiences in the villages, and offered something unique to remote, rural villages - especially involvement of professionals and the opportunity for children to be performing on stage alongside professional actors and seeing themselves in newspapers and on television. This is likely reproducible in other contexts, but not necessarily scalable as it would need to be tailored.

For peer review only

Olaide (2010)	Researchers, local drama groups, participants, stakeholders included government, health/medical and environmental establishment figures were involved in the design process.<br><br>The drama was intended to become the 'theatre of the people, by the people and for the people' (based on the Boalian concept of forum theatre) which aims not to find the 'best' solution but to produce a variety of options that could be used in the type of situation.	Communal research was conducted early in the process and identified that the most appropriate approach would be an 'inside-out approach' which would enable facilitators to interact within the community, gaining their trust and confidence and identify a 'joint panacea to the problems'.<br><br>The first step was the involvement of an existing and popular community-based theatre group in the dramatic endeavour data analysis, scenario development, improvisation rehearsal, performance and post-performance discussion. Also involved are some individuals within the community in the play presentation and the relevant themes were discussed after the performance in a live Q&A the programme took three days to put together.	Theatre performances, storytelling, later involved were drumming, singing and dancing which also made use of the songs that were known to them, most especially the nursing mothers and the pregnant women.<br><br>The performance comprised of 5 different but complementing sketches that were based on the causes and preventive measures of maternal mortality. The sketches were spiced up and linked together with different maternal songs that were folkloric, especially to the community members of the local government area.	The study appears to have caught the attention of and galvanised the community around issues of maternal mortality based on the qualitative reports cited in the paper.<br><br>The cost as with other local theatre groups should be low.<br><br>Long term outcomes were not observed.	No obvious barriers to scalability or reproduction were noted given low cost of theatre and success of other similar interventions.

For peer review only - http://bmjopen.bmj.com/site/about/guidelines.xhtml

Sanghvi et al. (2013)<br>Sanghvi et al. (2016)<br>Nguyen et al. (2019)	Partnerships with stakeholders were vital to expand the program's reach and to sustain program activities after initial funding ended. Partners were engaged in the research and design process to give implementing partners ownership of the strategy.<br><br>Government staff were trained by national and master trainers who were in turn trained by Alive and Thrive (A&T). The programme also launched a mass media campaign through inserts in newspapers (local journalists).	In each of the countries, Alive & Thrive convened stakeholders to review research to: (1) identify a few priority IYCF practices that would be the focus of the activities; (2) assess the degree to which select behaviours were feasible from family members' point of view and identified the conditions.<br><br>For all three countries, ProPAN methods were adapted and supplemented with additional tools, such as focus group discussions to gather information from frontline heath workers, fathers, grandmothers, and community leaders. The situational analysis in each country involved stakeholder consultations and reviews of existing data sets and reports to identify strategic choices and gaps in the data. Media audits, conducted by media research firms, guided two decisions: how much effort to devote to implementing or improving the Code of Marketing of Breast-Milk Substitutes, and what media channels to use for reaching different audience segments.	In all three countries, the mass media campaign containing emotionally appealing mini-dramas with various messages to spread the perception of recommended practices as normative was designed for a run of at least 2 years to build up audience recognition and recall. The campaign's television ads were loaded onto DVDs and shown on screens in outlying areas with low electricity, using portable generators, and the frontline worker training included a session using the television spots. Rural communities in media dark areas engaged through other mediums such as theatre.	Local adaptation, engagement of multiple stakeholders and scaling up were key elements of these projects. Combining face-to-face interventions for mothers with social mobilization and mass media was effective in improving IYCF practices.<br><br>The broad campaigns may both help and hinder sustainability. Helpful in that broad audiences through information diffusion can help transform norms quickly for many. A hinderance in the complexity of messaging with locally adapted schedules, content as well as their organisation. Partnerships with local organisations potential helped sustain the programmes after initial funding.	The campaign was delivered at a large scale, reaching an estimated 8.5 million. Scale-up was achieved through streamlining of tools and strategies, government branding, phased expansion through a local non-governmental implementing partner with an extensive community-based platform – and nationwide mainstreaming through multiple non-governmental organization and government programmes. The process has also been replicated in three countries. Without external (international) funding the scale of this study may not be replicable.

For peer review only - http://bmjopen.bmj.com/site/about/guidelines.xhtml

Sloand and Gebrian (2006)	Haitian Health Foundation (HHF), which is supported by both private donations and public funding, instituted fathers clubs in 1994. The fathers meet regularly, usually weekly or every 2 to 3 weeks, to learn about a wide range of child and family health topics and the related household- and family-level solutions. They discuss how to support their wives in the care of their children. They share family problems and help each other when needed. Meetings were described as 'very participatory'. The meetings are led by the fathers but attended by health agents (community health workers) and often a nurse who supervises the health agent. Together, they provide health education and support to the fathers.	Topics are decided by group discussion and fathers also engage in wider local infrastructure/project issues.	The focus of meetings is usually a child or family health issue, and discussion, songs, and skits are used to enhance learning. To reach out beyond the group, they help organise village health fairs with mothers' clubs or village health committees.	Fathers reported changes in knowledge, behaviour, social norms and skills, as well as health benefits (although these were not confirmed in observational studies). Father involvement is higher than expected based on other areas in Haiti.<br>Fathers report believing that the health and well-being of all the village children is the shared responsibility of all the adults in the village.<br>It is unclear if there are inequalities in access or participation for fathers.<br><br>The Fathers Club project has been running since 1994 in Haiti, so can be considered very sustainable in this context. However, long term outcomes were not formally evaluated/reported.	Low resources and low development context of this intervention imply high reproducibility across contexts. The use of existing health agents improves scalability also, however in contexts without a private/voluntary health foundation to provide support and organise reproducing and scaling may be more difficult.

For peer review only - http://bmjopen.bmj.com/site/about/guidelines.xhtml

Thuita et al. (2015); Mukuria et al. (2016)	International aid agency funded the project, which engaged volunteer community health workers (CHWs). These CHWs form community health committees and elect their leaders. In functional units, CHWs and committees are actively providing and overseeing community health surveys.<br><br>Each dialogue group selected one of its members to serve as the group mentor, who was trained and assisted by a volunteer CHW. Feedback was provided as well. Before each meeting, mentors selected the discussion topic based on member interest and then facilitated discussions and activities with group members.<br><br>Tea allowance was provided to all groups during meetings, however, fathers preferred money (100 ksh/ 1 USD).	Formative research designed key messages for fathers and grandmothers in households in western Kenya. Peer dialogue groups were used to facilitate behaviour change among fathers and grandmothers by helping them gain new knowledge, share experiences and reflections, and apply communication and problem-solving skills that in turn would facilitate behaviour change in mothers.<br><br>Existing health facilities were used to host events. Trained CHWs conducted home visits with study participants in both the intervention and comparison groups and shared oral and written complementary feeding and maternal nutrition messages. In the intervention areas, fathers or grandmothers were invited to participate in this visit.	Dialogue-based groups were used to engage infants' fathers and grandmothers to support optimal infant feeding practices. Group members participated in role plays, problem solving activities, storytelling, and cooking demonstrations; grandmothers also composed songs promoting recommended practices. They showcased what they were learning through songs, skits, dances, and testimonials from fathers, mothers, grandmothers, CHWs and children, at Community Bazaars.	Family-centred approach, through engaging fathers and grandmothers of infants to improve their knowledge of optimal infant feeding practices and to encourage provision of social support to mothers could help improve some feeding practices. It is unclear whether all groups were engaged in the activities. Arts were used for community mobilization activities.<br><br>The use of local infrastructure is promising for sustainability of the intervention. The bazaars are low cost also.	Low cost and resource usage. Builds on existing infrastructure.

For peer review only - http://bmjopen.bmj.com/site/about/guidelines.xhtml

Winter et al. (2021)	World Vision and Sesame Workshop collaboratively developed the WASH UP! program. WASH UP! combines a 12-week educational curriculum created by Sesame Workshop targeting students in grades 1-4 with school WASH infrastructure improvements carried out by World Vision. Teacher training is conducted by Sesame Workshop over the course of 3 days, and materials distribution and infrastructure improvements are funded and implemented by World Vision. The curriculum includes 12 sessions, each of which is approximately 30-40 min long and uses child-friendly pedagogical strategies such as play-based learning.<br><br>The twelve sessions were designed to be conducted before, during, or after normal school hours, at the discretion of the teacher. A learning object was distributed to a random sub-set of five of the 12 schools. Teachers provided the learning object to students with the instruction to take it home and discuss a key message about handwashing from the WASH UP! curriculum with their caregivers.	helping families to establish and maintain handwashing stations close to the place of child feeding rather than focusing on the message 'wash your hands with soap'.<br><br>Mass media and community mobilization were used to create an enabling environment by changing perceptions about using soap as the norm. Communication materials and a TV spot focused on convenience - establishing a handwashing close to the place of food preparation. A training session on handwashing stations was also inserted in the national IYCF training manual.	Activities include listening to the teacher read from a story book, playing interactive games, structured discussion of key curriculum messages, and watching two 10-min videos.	No evidence of effectiveness of children as agents of change.<br><br>Disruption to teacher's schedules and workloads were noted, this also was an image due to potential reputational damage for World Vision within such communities where they work. This necessitated some adaptation of scheduling of intervention delivery. Projectors and videos were included as part of the curricular materials, but three of the twelve schools did not have electricity, making one of the twelve sessions much more challenging to administer	Low cost, low resource (is theatre less expensive than video? Likely more engaging, more easily made participatory and local)

For peer review only - http://bmjopen.bmj.com/site/about/guidelines.xhtml

RAMESES CHECKLIST

List of items to be included when reporting a realist synthesis		Page number
TITLE	1 In the title, identify the document as a realist synthesis or review	1
ABSTRACT	2 While acknowledging publication requirements and house style, abstracts should ideally contain brief details of: the study's background, review question or objectives; search strategy; methods of selection, appraisal, analysis and synthesis of sources; main results; and implications for practice.	1
INTRODUCTIO N	3 Rationale for review<br>Explain why the review is needed and what it is likely to contribute to existing understanding of the topic area.	4-5
	4 Objectives and focus of review<br>State the objective(s) of the review and/or the review question(s). Define and provide a rationale for the focus of the review.	5-6
METHODS	5 Changes in the review process<br>Any changes made to the review process that was initially planned should be briefly described and justified.	6
	6 Rationale for using realist synthesis<br>Explain why realist synthesis was considered the most appropriate method to use.	5
	7 Scoping the literature<br>Describe and justify the initial process of exploratory scoping of the literature.	6
	8 Searching processes<br>While considering specific requirements of the journal or other publication outlet, state and provide a rationale for how the iterative searching was done. Provide details on all the sources accessed for information in the review. Where searching in electronic databases has taken place, the details should include, for example, name of database, search terms, dates of coverage and date last searched. If individuals familiar with the relevant literature and/or topic area were contacted, indicate how they were identified and selected.	6
	9 Selection and appraisal of documents<br>Explain how judgements were made about including and excluding data from documents, and justify these.	7
	10 Data extraction<br>Describe and explain which data or information were extracted from the included documents and justify this selection.	7-8
	11 Analysis and synthesis processes<br>Describe the analysis and synthesis processes in detail. This section should include information on the constructs analyzed and describe the analytic process.	8
RESULTS	12 Document flow diagram<br>Provide details on the number of documents assessed for eligibility and included in the review with reasons for exclusion at each stage as well as an indication of their source of origin (for example, from searching databases, reference lists and so on). You may consider using the example templates (which are likely to need modification to suit the data) that are provided.	Supplementar y Figure 1
	13 Document characteristics<br>Provide information on the characteristics of the documents included in the review.	9-10
	14 Main findings<br>Present the key findings with a specific focus on theory building and testing.	10-16
DISCUSSION	15 Summary of findings<br>Summarize the main findings, taking into account the review's objective(s), research question(s), focus and intended audience(s).	16-17
	16 Strengths, limitations and future research directions<br>Discuss both the strengths of the review and its limitations. These should include (but need not be restricted to) (a) consideration of all the steps in the review process and (b) comment on the overall strength of evidence supporting the explanatory insights which emerged. The limitations identified may point to areas where further work is needed.	21
	17 Comparison with existing literature<br>Where applicable, compare and contrast the review's findings with the existing literature (for example, other reviews) on the same topic.	17-21
	18 Conclusion and recommendations<br>List the main implications of the findings and place these in the context of other relevant literature. If appropriate, offer recommendations for policy and practice.	21-23
	19 Funding<br>Provide details of funding source (if any) for the review, the role played by the funder (if any) and any conflicts of interests of the reviewers.	23

For peer review only

BMJ Open

Co-produced, arts interventions for nurturing care (0-5 years) in Low- and Middle-Income Countries (LMICs): a realist review.

Journal:	*BMJ Open*
Manuscript ID	bmjopen-2023-083093.R2
Article Type:	Original research
Date Submitted by the Author:	17-Apr-2024

Complete List of Authors:	Gale, Nicola; University of Birmingham<br>Ahmed, Kalim; University of Birmingham<br>Diarra, Niélé ; University of Bamako<br>Manaseki-Holland, Semira; University of Birmingham College of Medical and Dental Sciences, Department of Public Health, Epidemiology and Biostatistics<br>Asamane, Evans; University of Birmingham<br>Sidibé, Cheick; University of Bamako<br>Touré, Ousmane; University of Bamako<br>Wilson, Michael; Loughborough University<br>Griffiths, Paula; Loughborough University, School of Sport Exercise and Health Sciences

**Primary Subject Heading**:	Global health
Secondary Subject Heading:	Health policy, Paediatrics, Public health
Keywords:	Public health < INFECTIOUS DISEASES, Community child health < PAEDIATRICS, Systematic Review

SCHOLARONE™
Manuscripts

I, the Submitting Author has the right to grant and does grant on behalf of all authors of the Work (as defined in the below author licence), an exclusive licence and/or a non-exclusive licence for contributions from authors who are: i) UK Crown employees; ii) where BMJ has agreed a CC-BY licence shall apply, and/or iii) in accordance with the terms applicable for US Federal Government officers or employees acting as part of their official duties; on a worldwide, perpetual, irrevocable, royalty-free basis to BMJ Publishing Group Ltd ("BMJ") its licensees and where the relevant Journal is co-owned by BMJ to the co-owners of the Journal, to publish the Work in this journal and any other BMJ products and to exploit all rights, as set out in our licence.

The Submitting Author accepts and understands that any supply made under these terms is made by BMJ to the Submitting Author unless you are acting as an employee on behalf of your employer or a postgraduate student of an affiliated institution which is paying any applicable article publishing charge ("APC") for Open Access articles. Where the Submitting Author wishes to make the Work available on an Open Access basis (and intends to pay the relevant APC), the terms of reuse of such Open Access shall be governed by a Creative Commons licence – details of these licences and which Creative Commons licence will apply to this Work are set out in our licence referred to above.

Other than as permitted in any relevant BMJ Author's Self Archiving Policies, I confirm this Work has not been accepted for publication elsewhere, is not being considered for publication elsewhere and does not duplicate material already published. I confirm all authors consent to publication of this Work and authorise the granting of this licence.

For peer review only

TITLE

Co-produced, arts interventions for nurturing care (0-5 years) in Low- and Middle-Income Countries (LMICs): a realist review.

ABSTRACT

Objectives: Community-based arts interventions have the potential to support contextually relevant nurturing care programmes and policies that adapt to different settings. Understanding the distinctive features of using the arts in local, culturally-specific ways in Low and Middle Income Countries (LMICs); how this varies by context; and gaining a better understanding of the perspectives on desirable outcomes for communities is important evidence that this review generates.

Design: We conducted a realist review of papers that covered outcomes related to child health or development (0-5years) AND arts-based approaches AND community-based, participatory approaches AND based in LMICs using a range of databases and other networks. A coding framework was developed covering context, intervention, outcomes, mechanisms, study, sustainability, transferability, and scalability.

Results: The included papers reported 18 unique interventions. Interventions covered 14 countries, with evidence lacking for South America, Arab countries, and parts of Africa. Lead authors came from mostly clinical science-based disciplines and from institutions in a different country to the country/countries studied. Intended outcomes from interventions included clinical, health systems/organisation, changes in practices/behaviours/knowledge/attitudes, and wider social and educational goals. We identified three demi-regularities (semi-predictable patterns or pathways of programme functioning): participatory design based on valuing different sources of expertise; dynamic adaptation of intervention to context; and community participation in arts-based approaches.

For peer review only - http://bmjopen.bmj.com/site/about/guidelines.xhtml

Conclusions: Our findings suggest that arts-based, nurturing care interventions have greater potential when they include local knowledge, embed into existing infrastructures, and there is a clear plan for ongoing resourcing of the intervention. Studies with better documentation of the lessons learned, regarding the intervention delivery process and the power dynamics involved, are needed to better understand what works, for whom and in which contexts.

STRENGTHS AND LIMITATIONS OF THE STUDY

- Systematic search of the international literature ensured good coverage of the field.
- Multi-disciplinary team authorship ensured realist synthesis methods could capture what works, for whom and in what circumstances.
- Searches were conducted in English so the review may have regional biases, as well as positive results publication bias.
- Inclusion of studies that report interventions meant that relevant contextual or conceptual papers, books or chapters, more common in the arts disciplines were not included.

BACKGROUND

This article reports the findings from a realist review and synthesis of the published literature on community-based arts interventions targeted at improving health and development of 0-5 year olds in low and middle-income countries (LMICs).

Nurturing care:

UNICEF's nurturing care framework provides a structure through which countries or communities can support parents and caregivers to provide an environment that ensures optimal health, nutrition and development for infants and young children and in which these individuals can live free from

For peer review only - http://bmjopen.bmj.com/site/about/guidelines.xhtml

threats and have opportunities to develop and learn using responsive interaction [1]. More than 5 million children did not survive to their fifth birthday in 2020 [2]. Furthermore, 43% (over 250 million) infants and young children in LMICs do not achieve their developmental potential for reasons including poverty, undernutrition and a lack of appropriate responsive and nurturing care and simulation [3,4]. Appropriate nurturing care has the potential to reduce mortality and morbidity and improve early childhood development [5]. To support the core elements of the nurturing care framework, programmes and policies are needed that are contextually relevant and take account of the requirements and different needs of all communities, because nurturing care is heavily embedded in local cultures and those seeking to implement health or child development interventions are influenced by local political and economic circumstances [6,7].

Power and participation:

Community-based interventions are often co-produced with local communities. The term co-production is highly debated, but broadly refers to a collaborative and reciprocal process of exchange between actors of differing backgrounds, to generate outcomes uniquely embedded in the different perspectives shared [8]. There are often significant challenges in implementing co-production in practice, such as knowledge and power asymmetries between those collaborating. Often power dynamics in these collaborations shift away from local expertise to professional expertise meaning that not all voices are heard [9-14]. Such power dynamics may produce interventions which are not sensitive to the specific issues, barriers and affordances of the context in which the intervention is implemented.

Many have argued that to overcome challenges and realise the purported benefits of co-production, collaborators must move beyond a ‘tick box’ or process-driven approach to involvement to partnerships that enable partnership synergy [11]. This is built through mutual trust and understanding between parties and takes into account contextual factors [15,16]. Careful

For peer review only

For peer review only - http://bmjopen.bmj.com/site/about/guidelines.xhtml

consideration of how interventions may draw on and influence local infrastructures and relationships is also important, to build sustainable collaborations [17,18].

Arts-based approaches:

Applied arts is an umbrella term that refers to work that seeks to use the arts as a tool to be applied to a social issue; other commonly used terms in the literature are Arts for Development, Arts for Social Change or Participatory Arts [19,20]. Arts-based interventions are usually carried out in group settings where they are used to undertake collective processing and/or for the communication of new information such as health advice, e.g. through dramatic or musical performances, bazaar event days with activities, competitions and prizes. Applied arts approaches are distinct from therapeutic approaches, such as Art Therapy, which are associated with improving individual health directly [21].

When practiced through local arts traditions, an intervention is embedded into local culture rather than imposed from outside, which is typically thought to make the intervention more relevant, coherent and engaging to community members including using existing community strengths [22]. When both performer and audience are involved in a live performance it can act to bind communities with a sense of togetherness, increased trust and common goals [22,23].

Rationale for review and objectives:

Given the growing global interest in arts-based approaches to health and child development interventions, there is a clear need to understand whether they are effective and, if so, how they work. There is growing understanding of the effectiveness of arts-based approaches [23,24-30] , and much scholarship in this field from the arts disciplines engages with issues such the artistry or process of artistic performance or creativity, or the role of artistic practice in interrogating concepts or cultures [31,32], but arts scholarship tends to focus less on evaluating interventions *per se.* Systematic reviews of the effectiveness of arts-based interventions in child health and development in LMICs are lacking.

For peer review only

Realist evaluation methodologies aim to ensure that context is incorporated in any analysis of effectiveness, using the refrain, 'what works, for whom, and in what circumstances?' [33-35]. Interventions that seek to address issues of improving nurturing care are often complex, addressing many layers of social and cultural practice, and influenced by political and economic context, so oversimplistic assessments that deem such interventions categorically 'effective' or not are problematic. The nuanced realist evaluation of arts-based nurturing care interventions in LMICs provided in this review will provide evidence to inform policy and intervention design to improve nurturing care in some of the world's most vulnerable contexts. It will also explore the sustainability of interventions and generalisability of findings, and specifically whether interventions might be reproducible in other contexts (usually with some adaptations to context), or scalable to a wider population.

To address these gaps, we conducted the first realist review and synthesis of the published and grey literature on community-based arts interventions targeted at improving health and development for 0–5-year-olds in Low- and Middle-Income Countries (LMICs). We identify (i) possible intervention mechanisms focusing on the distinctive feature of using the arts in local, culturally specific ways; (ii) relevant issues of contextual difference and (iii) a broad perspective on desirable outcomes for communities, rather than focusing solely on medical outcomes. Realist review is an effective method for analysing complex interventions that are highly context-dependent [33,34]. This can help inform future research as well as policy and intervention design and implementation in this field, by identifying what works, for whom and in what circumstances, and what elements are likely to make interventions more sustainable, transferable, or scalable. Additional resources about realist reviews can be found the RAMESES Project website.

The overarching review questions are:

i. What kinds of co-produced, arts-based, nurturing care interventions have worked in LMICs, for whom and in what circumstances?

For peer review only - http://bmjopen.bmj.com/site/about/guidelines.xhtml

ii. To what extent are successful interventions sustainable, transferable, or scalable?

METHODS

We applied the RAMESES quality and reporting guidelines [35].

Scoping the literature:

We worked with a team in the UK and Mali, including the intended users of the review, to clarify focus and prioritize research questions. We presented our initial ideas and later our preliminary findings to the MaaCiwara project steering committee. MaaCiwara is an ongoing study with collaborations between institutions in Mali and the UK examining the impact of an intervention in Mali for nurturing care, with significant arts-based elements [36]. Our preliminary objectives for this review were to identify examples of arts-based interventions co-produced between communities affected by poor child health, nutrition, and development outcomes and those involved in the public health/health promotion sector in LMICs and/or research organisations; to develop an understanding of the theoretical frameworks that inform these interventions or can help explain their effects, and to understand the mechanisms that contribute to success for these interventions. As the review progressed, we introduced a second research question focused on the long-term potential of these sorts of interventions to have an impact within the original context and in other similar ones. The findings from this review have been used to inform the data collection and analysis strategy for the primary empirical research.

Search strategy:

We searched for peer-reviewed journal articles that covered outcomes related to child health or development (0-5years) AND arts-based approaches AND community-based, participatory approaches AND based in LMICs (Supplementary Table 1). An experienced information specialist performed these searches. These terms were used to search EBSCO, OVID, ProQuest, Scopus, Web of Science and Child Adolescence and Education Studies databases.

For peer review only - http://bmjopen.bmj.com/site/about/guidelines.xhtml

Selection and appraisal of studies:

KA and NHD independently screened titles and abstracts for relevance. Where there was uncertainty, further discussions took place with PG and NG and we refined our relevance criteria in line with the outcomes of these discussions (Supplementary Table 2). The gradual refinement of these criteria helped focus the review questions and ensured coherent conceptualizations of the research area between research team members.

Data Extraction:

Given the wide range of intended outcomes and evaluation methods used, we did not attempt to classify in any categorical way whether or not an intervention had been effective, rather we attempted to unravel whether the papers were able to say something about 'what works, for whom and in what circumstances' and whether improvements could be sustained. We did this by identifying and analysing context-mechanism-outcome configurations from information that was available in the papers.

To address the first research question, the team developed an initial coding framework:

- **Context:** Country-Region, Socio-Cultural, Economic-Developmental, Legal-Political, Geographical-Physical
- **Intervention:** Agents, Co-production, Arts-based Methods, Dates, Design, Implementation
- **Outcomes:** Impact on Primary Outcome; Impact on Secondary Outcomes, Unintended outcomes/consequences.
- **Mechanisms:** Observed-Evidenced; Theorised
- **Study:** Aims, Constructs-Variables, Date, Limitations, Sampling, Theoretical Framework, Study design/Methods, First Author Location
- **Paths:** Interaction between Context/Intervention, Context/Outcomes, Intervention/Outcomes, & Context + Intervention → Outcomes

For peer review only - http://bmjopen.bmj.com/site/about/guidelines.xhtml

The ‘paths’ extraction was based on anything in the paper that spoke direct to the issues of the Context-Mechanism-Outcome configuration [33], even if a realist approach was not used. After agreeing the approach, papers were coded and data were extracted independently by KA and NHD, who then had a series of discussions to resolve differences.

To address the second review question, a second round of data extraction was conducted by NG and PG on reported or potential issues of:

- **Sustainability**
- **Transferability**
- **Scalability**

Data Analysis and Synthesis:

Our process for generating theories and adjudicating between them involved four stages involving NG, KA and PG meeting regularly, with emerging themes discussed in wider team meetings. First, we worked to compare and contrast the different interventions and the evaluation methods used, with specific reference to the ways in which the interventions focused on the key concepts of our research question, i.e. ‘co-production’ and ‘community arts-based’. Second, we looked for demi-regularities in the data [37] in order to generate a set of propositions about the social mechanisms at play in these interventions. Our discussions cohered around three themes for further exploration: developing sustainable partnerships; tailoring interventions to local context, and using arts-based approaches that engage with social norms. Third, we appraised each intervention with a series of questions centred on these three themes (Supplementary Table 3), in order to answer the question ‘what works, for whom and in what circumstances?’ Finally, we considered whether the interventions were reported to be or likely to be sustainable, transferable and/or scalable.

Patient and public involvement:

No patients or members of the public were involved in this literature review.

For peer review only - http://bmjopen.bmj.com/site/about/guidelines.xhtml

RESULTS

Study Characteristics:

Our database searches generated 940 unique results (Supplementary Table 4). After an initial screening of titles and abstracts, 175 results remained. At full text screening, 16 were deemed relevant to the review. Additionally, 7 papers from our informal search and consultation with the wider reference group were identified which met these inclusion and exclusion criteria and were added to our sample. Therefore, 23 papers were included in the review, which reported findings from 18 separate interventions (process overview in Supplementary Figure 1). In some cases, formative research or baseline characteristics were covered separately from evaluations of the effectiveness of the interventions resulting in multiple papers per intervention. The 18 interventions are summarized in Supplementary Table 5.

Global Coverage: These interventions covered 14 different countries, with the majority focussing on the Asia Pacific region (Bangladesh (n=3), Cambodia (n=2), Haiti, India, Malaysia, Nepal (n=2), and Vietnam), followed by the African region (Ethiopia, Kenya, The Gambia, Nigeria (n=2), Uganda, and Zambia (n=2)), and one paper from South America (Peru). This shows that the English language evidence base is not providing much evidence for the South American or Arab regions, as well as large parts of Africa.

Authorship Location and Disciplines: Given the focus on co-production in the review and the interdisciplinary nature of the interventions, there was a notable imbalance in the pattern of authorship. It was often unclear whether the projects had been initiated by those leading the publication or by others, such as local teams, but out of 23 papers, 17 have first authors affiliated to an institution in a different country to the country or countries being studied in the paper. In addition, most academic outputs were led from the health sciences and only one paper was led by

For peer review only - http://bmjopen.bmj.com/site/about/guidelines.xhtml

researchers in the arts and humanities and two by social scientists. One paper was led by a team in Civil and Environmental Engineering.

Intended outcomes: There were a range of different intended outcomes from interventions, from the clinical (e.g. maternal mortality rates, malaria rates, anthropometry), the organisational (e.g. utilisation of services), to changes in practices/behaviours (e.g. changed dietary practices, exclusive breastfeeding, sharing of messages within families), and changes in knowledge or attitudes (e.g. dissemination of messages, health information retention) and wider social and educational goals (e.g. women's empowerment, school readiness).

Evaluation methods: A range of qualitative and quantitative methods were used to evaluate the interventions, including cluster randomised controlled trials or impact assessments (non-blinded), quasi-experimental studies, a prospective cohort study, pre-post outcome evaluations (with or without control), cross-sectional surveys, interviews, focus groups and digital story creation. In a few cases, the focus was on descriptive case studies or process descriptions, rather than on evaluating outcomes.

Main Findings:

We identified three main demi-regularities: participatory design based on valuing different sources of expertise; dynamic adaptation of intervention to context; and community participation in arts-based approaches. These three pathways were unevenly realised across the different interventions described and evaluated in the papers (Supplementary Table 6) but can serve as a basis for future studies, or for those designing new or adapting interventions to reflect on.

Programme Theory 1: Participatory design based on valuing different sources of expertise

While our inclusion criteria required some element of 'co-production', there was a huge range of approaches, including those that were clearly researcher-led, with limited involvement of local people, local artists or other stakeholders, those that were driven by the interests of funders,

For peer review only

particularly NGOs, and those where local people, local artists or local researchers drove the projects forward. In some cases, the co-production elements were primarily enacted during the design stages [38], while others were mainly at the implementation stage through the adaptation of an existing intervention to local context [39]. Others provided continuing opportunities for communities to engage throughout the length of the programme [40].

Co-production models ranged from very light touch co-production models to co-production that started at the beginning of the project and followed throughout the programme of activities. At the lighter end was a model where community health volunteers talked with local communities in discussion groups to get their involvement in the intervention, but where the intervention was designed externally to the community [41]. Other lighter touch co-production models included a programme of activities developed outside of the community but delivered with local artists/ drama groups to engage communities in the production of the intervention success [42] or a model where an existing intervention was adapted with local community input [43]. At the other end of the spectrum, some projects used co-production models that started from the beginning of the project to design the intervention in partnership with the community [38], or where communities develop the materials and stories that drive the intervention's focus messages [44], or where the intervention was framed around collective learning between artist and the community [40].

There were some limitations in the reporting of co-production approaches. Often there was very little critical reflexive elaboration about the power structures in which the interventions were being designed and implemented, or what the challenges and benefits of harnessing community power structures were (such as who was included and who was not in the process). Another area that was underdeveloped was to what extent the resourcing of the intervention or the evaluation influenced the co-production. It is likely that there were pragmatic requirements to deliver public health interventions for funders, combined with potent power dynamics in communities, which could intensify the exclusion of already 'hard to reach' groups.

For peer review only - http://bmjopen.bmj.com/site/about/guidelines.xhtml

Programme Theory 2: Tailoring of intervention to context

All papers recognised to some degree the need to develop interventions what were tailored to the local context and employed a range of different methods to do so. These ranged from formal formative research processes that used literature reviewing, as well as qualitative (interviews, visual methods) and quantitative methods (surveys, analysis of routine or existing data sets, media audits) to understand the pre-existing social norms and behavioural patterns related to nurturing care [43,45,46], or to identify key messages/changes to focus on. In other studies, a pilot or series of pilots were used to refine the intervention, drawing on feedback from those delivering, receiving, and funding the interventions [38].

Some interventions were built around existing community groups, such as 'Fathers Clubs' in rural Haiti, where the social groupings were already well established and health messages were incorporated into these in a way that was led by the fathers themselves, with support from health workers [47], or the women's groups in rural Zambia [45]. In addition, existing infrastructure was often used, such as health facilities or community meeting spaces [41,48].

Some papers acknowledged the importance of enabling or constraining environments, both physical (such as the location of handwashing stations or providing spaces for infant feeding) and not just focusing on health education messages. The importance of the social environment (such as the need to change perceptions about using soap in mass media campaigns) [49] was also identified. These approaches supplemented or complemented the arts components.

A significant limitation in the data was that authors did not always report on the opportunities for feedback from stakeholders, and so it is unclear whether those opportunities existed or not. Even where feedback opportunities are mentioned, it is not always clear what impact the feedback had on the subsequent delivery of the intervention, if any. Where performing arts were part of the intervention, there was very little reported in papers on how performing arts practitioners

For peer review only - http://bmjopen.bmj.com/site/about/guidelines.xhtml

contributed to the overall design or content of the performance, so it is hard to judge the extent to which they influenced the artistic approaches taken.

Potentially relevant to the context was other similar or related public health or NGO programmes that were being undertaken in the region, and any impact they could have on outcomes, but these were rarely reported.

Programme Theory 3: Community participation in arts-based approaches

Types of arts-based activities were wide-ranging and included: storytelling, story-acting, visual cues, interactive role-play, posters, comic books, nursery rhymes and songs, puppet shows, drama, drawing, singing competitions, animations, stories, tv promotions, games, dancing, fashion shows, comedy sketches, speeches, professional singing performances, street drama, skits (sketches), kitchen makeovers, decorating kitchens, letter exchanges, family drama, folk songs, cookery demonstrations, photo displays, Kalajarta (folk theatre), rupakas (musical dramas), writing scripts, community videos, TV spots with mini-dramas of intervention messages, radio messaging, brochure design, digital storytelling, drumming, testimonials, cartoon films, mass media campaigns, game based and music based education, recitals, storybook reading, interactive games, and watching videos.

Less than half of the interventions delivered the arts components in a way that included specialist arts practitioners, such as local drama groups, traditional communicators, traditional theatre groups, songwriters, photographers, and other artists. Most interventions delivered the arts components through or with other members of the community, such as community health workers (normally with basic education), parents (mostly mothers) and wider community groups. In some cases, workers from NGOs were used to deliver intervention materials. It was much less common for papers to mention local or national government agents as involved in intervention delivery.

For peer review only - http://bmjopen.bmj.com/site/about/guidelines.xhtml

For peer review only

Very few papers commented on their theories or principles of how the arts were expected to support the intervention, with the exception of Olaide's paper (written by a Performing Arts scholar) which noted that it drew on Boal's concept of 'forum theatre' which is part of a broader concept of 'theatre of the oppressed', which is a widely used tool particularly in India, and eastern and southern Africa to facilitate social change [50]. Most studies were reported by health scientists and in health journals so in very few cases was a detailed understanding of the process of arts production or performance interrogated in a critical or scholarly way. The intended mechanisms of change remained implicit, the arts were reported as a 'black box' conceptually and practically (no knowledge of the internal workings).

Other limitations to the reporting of the arts elements of the interventions included few detailed analyses of who was involved in the activities, how they were invited, and which parts of the communities may have been excluded for cultural, social, political, or economic reasons.

Finally, there was little consideration of how the arts activities aligned or not with broader community activities or norms, such as the provision of hospitality.

To what extent were the interventions sustainable?

Approximately half of the studies did not record long-term outcomes which makes it difficult to judge their sustainability. There are of course issues with measuring effectiveness long-term, such as leakage of intervention into control groups [45,51]. Key to sustainability is whether change can be maintained after the withdrawal of the research team [52].

A number of the studies claimed that the engagement of local arts meant that the studies would be more sustainable. The reasons given were that the arts interventions promoted willingness to engage [53] or included celebration as a motivation for continuing [51]. Where interventions drew on local resources, this was often considered to make them more sustainable or low cost [40,43,48,51,54,55]. Others argued that the use of arts would be better for promoting

For peer review only

retention/memory of the intervention messages [51,56] and that arts-based interventions help to create a positive attitude and contribute to people being more friendly to each other to promote sustainability [40]. Positive motivational drivers particularly embedded in arts-based messaging was seen to be more effective than negative drivers such as issuing defecation fines [39].

Other themes on claims for sustainability were that the diverse and intensive nature of activities over time promoted sustainability [39], and that interventions were more realistic to implement when they were embedded in local infrastructure and resources so that people can access the intervention [44,45]. It was, however, also noted that ensuring that those delivering the intervention are not distracted from their other duties when drawing on local infrastructure was also important, as well as ensuring appropriate additional incentivisation for involvement [45,49]. Local infrastructure issues, such as a lack of electricity or equipment, made the delivery of materials such as videos challenging and affected sustainability [49]. A final issue in sustainability was whether other members of the family and community (beyond the mother) were brought in to engage with the intervention [41] because broader family support was reported to bring longer-term sustainability of messaging.

To what extent were interventions transferable?

Systematic participatory approaches simplify the process of contextual adaptation[53] enabling the process of developing and delivering the intervention to be replicated (even if the content is different). Interventions that use existing staff, e.g. teachers or community health workers [38,47], to deliver the intervention find that this promotes transferability into contexts where the health or education systems [55] or infrastructure [44,45] are similar and these professionals or paraprofessionals receive broadly similar training. However, there is always a risk that staff groups in different contexts may not buy into the intervention. In addition, varying relationships between governments and NGOs were a limitation to transferability in some contexts [52].

To what extent were interventions scalable?

For peer review only - http://bmjopen.bmj.com/site/about/guidelines.xhtml

Small-scale interventions can limit the potential for scalability[39] particularly where they are reliant on individual motivation and momentum. Broad campaigns that capture large audiences quickly are more likely to be scalable, but the specificity of messaging can be challenging [57], which may limit the effectiveness or traction of the messages. Elements of group interaction also maximise the efficient use of resources [53], making the intervention more scalable, as well as having the potential to enhance community ties. A further point raised on scalability of arts-based interventions was when tried and tested health intervention materials were used alongside arts-based approaches leading to improved buy in for scaling [58].

Major limitations around scalability within LMICs are cost and resource, with many of these interventions seeking to minimize the resource burden on government or communities [40,41,43,47,52,55,56]. Examples of this are using local storytelling [59] or theatre [56, 58] which are cheaper than the production costs of video making [49]. Some costs can be prohibitive for scaling e.g. TV ads [52] and cameras on smartphones [44]. For this reason, NGO support and finances can be critical requirements for scalability [46,47,54-56, 60].

DISCUSSION

Summary of findings:

We identified three main programme theories, suggesting the importance of (i) participatory design based on valuing different sources of expertise; (ii) tailoring of the intervention to the local political and cultural context, and (iii) community participation in arts-based approaches. We found mixed evidence on sustainability of interventions, but that those embedded in local infrastructure and culture were more likely to be sustainable. We found that transferability of the interventions to other contexts, depended on the social and political organisation of health and child development activities (such as schools and community health workers). We found that the scalability of the community-arts interventions was variable, depending on the scale and levels of integration of the

For peer review only - http://bmjopen.bmj.com/site/about/guidelines.xhtml

For peer review only

arts approaches within wider programmes of health and child development work as well as the availability of financial resources.

Contribution to the literature (empirical):

Our findings on community-based arts interventions targeted at improving health and development outcomes for 0-5 year olds in Low and Middle Income Countries (LMICs) have revealed a number of gaps in current knowledge and understanding of what works for whom in which contexts.

The literature on interventions currently lacks input and leadership from academics working in the arts, humanities, and the social sciences. The lack of inclusion of arts scholars means there is a lack of theory integrated into discussions on how the arts were expected to support the interventions delivered, and it is unclear what was actually done in practice with local artists. Arts scholarship does engage with some of these issues [31] but not always in an interdisciplinary way where it is linked to outcomes of interventions (hence not meeting our review inclusion criteria). The lack of social science input likely leads to a dominant focus of the literature on outcomes as opposed to the processes used to achieve these. While there are literatures that suggest that the arts have the potential to break down power dynamics and provide communities a sense of ownership of the intervention with audiences owning the art or the performance [61-63], these critical approaches were not used to interpret the findings for these interventions. This means that opportunities to learn about what works for whom and in which contexts were missed.

The lack of local lead authors of studies and insufficient studies from some regions including West and Southern Africa and South America means that contextual understanding is often lacking in the literature reviewed and this gap is particularly big for the regions with no pre-existing evidence.

The majority of the studies included in this review used a model of co-production that involved bringing in communities to adapt existing interventions at a later stage of the development process as opposed to including communities from conceptualisation. The minority of studies that did work with communities throughout the process to co-produce were able to document benefits including

For peer review only

For peer review only - http://bmjopen.bmj.com/site/about/guidelines.xhtml

time to build trust, community capacity, empowering local leadership, and developing a true collaboration. These factors were all relevant for intervention sustainability because they may empower the community to take the intervention forward after the study ends. Overall, these elements were poorly reported, although this may reflect the focus and limited words counts in target journals.

Studies that entered communities early to co-design were better placed to draw on existing health systems including community health workers, social infrastructures such as fathers' or mothers' groups, and community facilities such as community spaces. Using existing infrastructures saves investment in time and resources to build and grow social systems and staffing infrastructures to support these types of intervention and supports sustainability. Studies that did use existing structures reported the importance of the level of adequate functioning of these systems to positively influence intervention outcomes. Ongoing training of workers in the system was also reported to be important to maintain the success of intervention messaging and to support intervention sustainability beyond the study. This review also identified studies that did not report interaction with existing community structures such as health systems which was a missed opportunity for joined up thinking and promoting intervention sustainability.

Contribution (theoretical)

The interrelatedness and interdependence of the three themes (co-production, local adaptation, and arts-based delivery) underpins the theoretical contribution of this review. While co-production and local adaptation offer two elements of 'scaffolding' that are necessary but not always sufficient for change, our findings suggest that the arts-based delivery approach can act as a bridge for overcoming some of the contextual and political challenges of delivering nurturing care interventions in LMICs. The use of realist methods for synthesizing the literature allowed us to explore multiple and intersecting mechanisms of action, and to explore those mechanisms within different contexts. It allowed us to see that the success of the arts elements is dependent on

For peer review only - http://bmjopen.bmj.com/site/about/guidelines.xhtml

effective collaboration and adapting to context, and to see that the arts can enhance and enact the benefits of collaborative working and careful local adaptation processes. Nevertheless the lack of explicit reporting in papers on the mechanisms through which the arts work to enact change means that we are left with limited knowledge about what works for whom in what context. For instance we have learned little about audience involvement in the arts, cultural embeddedness of the arts, how the arts works most effectively with the public health services, how the arts messages can be effectively shaped by those who know the health elements, the role of knowledge and background in the health topic of the artists, inclusion or inclusion of certain population groups through the arts, the role of the arts venues, and how arts interact with social norms. All of these would be important to gain understanding of the true role of the arts in facilitating nurturing care.

There is evidence to suggest the importance of working toward synergy between partners[11] in both goals and processes [64], and of including local people, community leaders, public health and education professionals, policy-makers, and researchers, as well as funders of nurturing care interventions, in order to achieve more than each could alone [65]. Our findings supported this through stressing the value of regular formal [53] or informal feedback opportunities [40] and regular participatory meetings to support sustainability [47]. This builds trust [15,66] and transparency [67,68].

When collaborations also acknowledge that interventions must be tailored to context in both the design and implementation phases, more can be achieved. Complementing or integrating with existing educational and health infrastructures and human resources was identified as being key, as was an understanding of cultural and social norms [39,42,49] for sustainable change.

However, a key risk to effective co-production and tailoring to local context is the dysregulating effect of power imbalances. Enabling empowerment of those with less historical or cultural power in collaborations is a vital element of success [16]. The review has highlighted how challenging it is for collaborations to achieve high levels of balance and equity in contributions, and how rare it is for

For peer review only

these issues to be explicitly or critically reported. Our analysis can make an additional theoretical contribution here by identifying the potential for arts-based approaches to act as a bridge for some of these challenges of power imbalances through practical and political pathways.

First, *practically*, our findings suggest that engagement with community arts increases the potential effectiveness of the intervention in achieving (shared) goals of improved health and child development. Both the arts and community aspects support engagement in a shared experience of performance and through the congruence of local arts with local culture. Our findings suggest that there are two elements to this: increasing motivation to engage, and shifting long standing social norms. Positive motivation is engendered though things like a festival-like atmosphere, diversity of art forms, and the focus on positive impacts of change. The social dimensions of motivation may be particularly encouraged by communal experiences which contribute to group binding [22,23]. The use of local arts, local performers and organisers [56,58] and even local stories [42] increases the sense of ownership [40]. Interventions that are able to acknowledge and then shift long-established social norms from within are much more likely to be sustainable, rather than introducing external ideas. While the studies in the review rarely explicitly commented on social norms, our analysis was able to identify possible elements of the individual, social, material and institutional domains [69] in which social norms operate. The active engagement and the shared memories of an event or experience were considered to promote discussion of intervention materials, improve its retention individually and, importantly, its diffusion through social networks, reaching beyond intervention participants [51,56].

Second, *politically,* our findings suggest that these sorts of community arts-based delivery approaches, when combined with co-production and local adaptation, have the potential to disrupt some of the neo-colonial tendencies in global health research and practice. This involves shifting the balance of power away from Western biomedical models, towards communities and their historical and cultural modes of artistic expression and cultural re/production. Culturally embedded arts as

For peer review only - http://bmjopen.bmj.com/site/about/guidelines.xhtml

opposed to non-culturally embedded arts have the most potential to achieve this outcome. Even the frequent delivery of these interventions in local languages suggests they are at least partially outwith the surveillance of global health researchers. While in some cases attempts to address power imbalances were evident, such as minority outreach and platforming during performances [59]; the targeting of women's empowerment through agricultural work and education [45]; targeting father and grandmother involvement in nurturing care practices [41,48], or setting up partnership agreements [70], in many cases this was not addressed explicitly in the papers.

Strengths and limitations of the review

The review only captures published peer-reviewed journal articles. This likely results in a positive results publication bias and a lack of reports on arts-based interventions (grey literature). While we did not explicitly exclude French and Spanish articles (we had the language capacity in the team), we conducted the searches in English only. Despite searching a wide range of databases, we have identified that the papers that have been included are focussed on health sciences rather than the arts/social science parts of the research. Many arts scholars publish in monographs or edited collections which are not always effectively captured through database searches.

Recommendations for further research

There is a significant gap in the international literature, for studies that are led by researchers from LMICs and/or by those in the arts, humanities and social sciences. Bringing these voices into the research would enable better integration of the theoretical underpinnings regarding the potential success of arts-based intervention processes in nurturing care interventions. It would also facilitate better understanding of intervention processes and cultural context of these. Specific steps could be considered to ensure this can happen, such as leadership from LMICs, devolving funding decisions, and supporting and funding projects led from the arts, humanities and social sciences as well as the health and clinical sciences. Methods for ongoing reflective evaluation of the quality and equity in collaborations would be useful. Contextual issues may be relevant, including sources of funding

For peer review only - http://bmjopen.bmj.com/site/about/guidelines.xhtml

being weighted toward health funders and the motivation for academics to publish in high impact journals, which tend to be more STEMM-focused. Finally, reporting in journals of interventions with arts should include better documentation of their learning regarding the process of intervention delivery and the power dynamics involved in this.

Recommendations for policy and practice

Nurturing care interventions are complex involving the health system, early education providers, social workers, families, extended families and community leadership structures. As we have shown in this review there are attempts taking place to incorporate the practical and political power of the arts into intervention design to empower communities to own nurturing care interventions, to facilitate message sharing and to embed intervention messaging into the culture of communities. Our findings suggest that these types of intervention have the potential for greater success in being contextually relevant when they include local knowledge including artists, are evaluated by teams that include those with experience in the arts, include collaborative work between artists and public health experts to adapt and deliver the intervention, and where local researchers take a lead or active role in the intervention delivery, evaluation and reporting.

There is also evidence in this review that where arts-based interventions embed into existing infrastructures (e.g. health services) and there is a clear plan for ongoing resource for training of those delivering the intervention that sustainability is likely. To better understand what works for whom in which contexts when designing nurturing care arts-based interventions there is a need for studies to better document their learning regarding the process of intervention delivery and the power dynamics involved in this.

CONCLUSIONS

The findings of this realist review contribute to our understanding of the potential impact of co-produced, arts interventions for nurturing care in LMICs. Using arts approaches had the potential to act as a bridge for some of the challenges of power imbalances between researchers, health and

For peer review only

For peer review only - http://bmjopen.bmj.com/site/about/guidelines.xhtml

education professionals, and communities, that tailoring interventions to context and participatory design could not achieve alone. Interdisciplinary investigations integrating insights from arts scholarship and from the public health fields could support future research.

Footnotes:

Acknowledgements: In addition to our core research team, we would like to thank Rachel Posaner for her work developing and executing the search strategies for this paper. We would also like to thank the wider MaaCiwara team for their support.

Contributors: NG: Securing funding; study design; data collection, extraction and analysis; manuscript drafting; manuscript development and review. KA: Study design; data collection, extraction and analysis; manuscript drafting; manuscript development and review. NHD: Data collection, extraction and analysis; manuscript development and review. SMH: Securing funding; manuscript development and review. AE: Manuscript development and review. CS: Securing funding; manuscript development and review. OT: Securing funding; manuscript development and review. MW: Manuscript development and review. PG: Securing funding; study design; data collection, extraction and analysis; manuscript drafting; manuscript development and review.

Funding: This research was funded by Medical Research Council (MRC), UK Research and Innovation (UKRI) Global Challenges Research Fund (GCRF) MR/T030011/1. The funder of this study has no role in the design, conduct, collection of data, analysis or writing of outputs.

Competing interests: None declared.

Provenance and peer review: Not commissioned; externally peer reviewed.

Data sharing statement: All papers included in the review are available on the journal websites.

ETHICS APPROVAL

For peer review only

For peer review only - http://bmjopen.bmj.com/site/about/guidelines.xhtml

562 Ethical approval was not required for this literature review.

563 **REFERENCES**

564 1. Britto PR, Lye SJ, Proulx K, *et al.* Nurturing care: promoting early childhood development. 565 *The Lancet*. 2017;389:91–102. doi: 10.1016/S0140-6736(16)31390-3

566 2. UNICEF. Levels and trends in child mortality. United Nations Inter-agency Group for Child 567 Mortality Estimation 2021. https://data.unicef.org/resources/levels-and-trends-in-child-mortality/ 568 (accessed 21 August 2023)

569 3. Lu C, Cuartas J, Fink G, *et al.* Inequalities in early childhood care and development in 570 low/middle-income countries: 2010–2018. *BMJ Global Health*. 2020;5: doi:10.1136/bmjgh-2020- 571 002314

572 4. Richter LM, Daelmans B, Lombardi J, *et al.* Investing in the foundation of sustainable 573 development: pathways to scale up for early childhood development. *Lancet*. 2017;389:103–18. 574 doi:10.1016/S0140-6736(16)31698-1

575 5. UN. Sustainable Development Goals. 2018. https://www.who.int/europe/about-us/our- 576 work/sustainable-development-goals (accessed 21 August 2023)

577 6. Barnish MS, Tan SY, Taeihagh A, *et al.* Linking political exposures to child and maternal 578 health outcomes: a realist review. *BMC Public Health*. 2021;21:127.

579 7. Barnish MS, Tan SY, Robinson S, *et al.* A realist synthesis to develop an explanatory model of 580 how policy instruments impact child and maternal health outcomes. *Social Science & Medicine*. 581 2023;339:116402.

582 8. Durose C, Perry B, Richardson L. Is co-production a 'good' concept? Three responses. 583 *Futures*. 2022;142: doi:10.1016/j.futures.2022.102999

For peer review only - http://bmjopen.bmj.com/site/about/guidelines.xhtml

9. Egid BR, Roura M, Aktar B, *et al.* ‘You want to deal with power while riding on power’: global perspectives on power in participatory health research and co-production approaches. *BMJ Global Health*. 2021;6: doi:10.1136/bmjgh-2021-006978

10. Yassi A, Spiegel JB, Lockhart K, *et al.* Ethics in Community-University-Artist Partnered Research: Tensions, Contradictions and Gaps Identified in an ‘Arts for Social Change’ Project. *J Acad Ethics*. 2016;14:199–220. doi:10.1007/s10805-016-9257-7

11. Keneth, Bamuturaki. Participatory Research in Theatre for Development: An Evaluative Paradigm of the Walukuba Project in Eastern Uganda. *Consciousness, Literature and the Arts*. 2016;17.

12. Goncalo JA, Duguid MM. Follow the crowd in a new direction: When conformity pressure facilitates group creativity (and when it does not). *Organizational Behavior and Human Decision Processes*. 2012;118:14–23. doi: 10.1016/j.obhdp.2011.12.004

13. Bechtoldt MN, De Dreu CKW, Nijstad BA, *et al.* Motivated information processing, social tuning, and group creativity. *Journal of Personality and Social Psychology*. 2010;99:622–37. Doi: 10.1037/a0019386

14. Schulz AJ, Israel BA, Lantz P. Instrument for evaluating dimensions of group dynamics within community-based participatory research partnerships. *Evaluation and Program Planning*. 2003;26:249–62. doi:10.1016/S0149-7189(03)00029-6

15. Jagosh J, Bush PL, Salsberg J, *et al.* A realist evaluation of community-based participatory research: partnership synergy, trust building and related ripple effects. *BMC Public Health*. 2015;15:725. doi:10.1186/s12889-015-1949-1

16. George AS, Mehra V, Scott K, *et al.* Community Participation in Health Systems Research: A Systematic Review Assessing the State of Research, the Nature of Interventions Involved and the Features of Engagement with Communities. *PLOS ONE*. 2015; doi: 10.1371/journal.pone.0141091

For peer review only

17. Kohrt BA, Asher L, Bhardwaj A, *et al.* The Role of Communities in Mental Health Care in Low- and Middle-Income Countries: A Meta-Review of Components and Competencies. *International Journal of Environmental Research and Public Health*. 2018;15:1279. doi:10.3390/ijerph15061279

18. Greenhalgh T, Jackson C, Shaw S, *et al.* Achieving Research Impact Through Co-creation in Community-Based Health Services: Literature Review and Case Study. *The Milbank Quarterly*. 2016;94:392–429. doi:10.1111/1468-0009.12197

19. Stupples P. Creative contributions: The role of the arts and the cultural sector in development. *Progress in Development Studies*. 2014;14:115–30.

20. Cooke P, Soria-Donlan I. *Participatory Arts in International Development*. 1st ed. Routledge 2019.

21. Slayton SC, D'Archer J, Kaplan F. Outcome Studies on the Efficacy of Art Therapy: A Review of Findings. *Art Therapy*. 2010;27:108–18.

22. Stewart L, McConnell BB, Darboe B, *et al.* Social singing, culture and health: interdisciplinary insights from the CHIME project for perinatal mental health in The Gambia. *Health Promotion International*. 2022;37:i18–25.doi: 10.1093/heapro/daab210

23. Faigin D, Stein C. Community-Based Theater and Adults with Psychiatric Disabilities: Social Activism, Performance and Community Engagement. *American journal of community psychology*. 2014;55. doi:10.1007/s10464-014-9695-6

24. Barnish MS, Barran SM. A systematic review of active group-based dance, singing, music therapy and theatrical interventions for quality of life, functional communication, speech, motor function and cognitive status in people with Parkinson's disease. *BMC Neurology*. 2020;20:371.

25. Barnish MS, Nelson-Horne RV. Group-based active artistic interventions for adults with primary anxiety and depression: a systematic review. *BMJ Open*. 2023;13:e069310.

For peer review only

For peer review only - http://bmjopen.bmj.com/site/about/guidelines.xhtml

26. Logie CH, Dias LV, Jenkinson J, *et al.* Exploring the Potential of Participatory Theatre to Reduce Stigma and Promote Health Equity for Lesbian, Gay, Bisexual, and Transgender (LGBT) People in Swaziland and Lesotho. *Health Educ Behav*. 2019;46:146–56. doi: 10.1177/1090198118760682

27. Torrissen W, Stickley T. Participatory theatre and mental health recovery: a narrative inquiry. *Perspect Public Health*. 2018;138:47–54. doi:10.1177/1757913917723944

28. Gallant K, Litwiller F, Hamilton-Hinch B, *et al.* Community-Based Experiential Education: Making It Meaningful to Students Means Making It Meaningful for Everyone. *SCHOLE: A Journal of Leisure Studies and Recreation Education*. 2017;32:146–57. doi:10.18666/SCHOLE-2017-V32-I2-3

29. Putland C. Lost in Translation: The Question of Evidence Linking Community-based Arts and Health Promotion. *J Health Psychol*. 2008;13:265–76. doi:10.1177/1359105307086706

30. Rossiter K, Kontos P, Colantonio A, *et al.* Staging data: Theatre as a tool for analysis and knowledge transfer in health research. *Social Science & Medicine*. 2008;66:130–46.doi: 10.1016/j.socscimed.2007.07.021

31. Baxter V, Low K. *Applied Theatre: Performing Health and Wellbeing*. 1st ed. Bloomsbury Publishing 2017.

32. Sextou P. A Theatre-in-Education Study of Multicultural Understanding in Hellas. *Youth Theatre Journal*. 2006;20:77–93.

33. Pawson R, Tilley N. An introduction to scientific realist evaluation. *Evaluation for the 21st century: A handbook*. 1997;405–18.

34. Jagosh J, Pluye P, Wong G, *et al.* Critical reflections on realist review: insights from customizing the methodology to the needs of participatory research assessment. *Research Synthesis Methods*. 2014;5:131–41. doi:10.1002/jrsm.1099

For peer review only - http://bmjopen.bmj.com/site/about/guidelines.xhtml

35. Wong G, Greenhalgh T, Westhorp G, *et al.* RAMESES publication standards: realist syntheses. *BMC Medicine*. 2013;11:21. doi:10.1186/1741-7015-11-21

36. Asamane EA, Quinn L, Watson SI, *et al.* Protocol for a parallel group, two-arm, superiority cluster randomised trial to evaluate a community-level complementary-food safety and hygiene and nutrition intervention in Mali: the MaaCiwara study (version 1.3; 10 November 2022). *Trials*. 2023;24:68.

37. Sayer A. *Realism and Social Science*. London: SAGE Publications Ltd 2000. doi: 10.4135/9781446218730

38. Gautam OP, Schmidt WP, Cairncross S, *et al.* Trial of a Novel Intervention to Improve Multiple Food Hygiene Behaviors in Nepal. *Am J Trop Med Hyg*. 2017;96:1415–26.

39. McMichael C, Robinson P. Drivers of sustained hygiene behaviour change: A case study from mid-western Nepal. *Social science and medicine*. 2016;163:28.

40. Olaide NT. Maternal mortality and theatre intervention: A case study of Oluyole local government in Ibadan of Nigeria. *US-China Foreign Language*. 2010;8:52–62.

41. Thuita FM, Martin SL, Ndegwa K, *et al.* Engaging fathers and grandmothers to improve maternal and child dietary practices: Planning a community-based study in western Kenya. *African Journal of Food, Agriculture, Nutrition and Development*. 2015;15:10386–405. doi:10.4314/ajfand.v15i5

42. Chea Nguon, Lek Dysoley, Chan Davoeung, *et al.* Art and theatre for health in rural Cambodia. *Global Bioethics*. 2018;29. doi:10.1080/11287462.2017.1411762

43. Buba Manjang, Hemming K, Bradley C, *et al.* Promoting hygienic weaning food handling practices through a community-based programme: intervention implementation and baseline

For peer review only

For peer review only - http://bmjopen.bmj.com/site/about/guidelines.xhtml

characteristics for a cluster randomised controlled trial in rural Gambia. *BMJ Open*. 2018;8. doi:10.1136/bmjopen-2017-017573

44. Limaye NP, Rivas-Nieto AC, Carcamo CP, *et al.* Nuestras Historias- Designing a novel digital story intervention through participatory methods to improve maternal and child health in the Peruvian Amazon. *PLoS ONE*. 2018;13:e0205673.

45. Kumar N, Nguyen PH, Harris J, *et al.* What it takes: evidence from a nutrition- and gender-sensitive agriculture intervention in rural Zambia. *Journal of Development Effectiveness*. 2018;10:341–72.

46. Nguyen PH, Frongillo EA, Kim SS, *et al.* Information Diffusion and Social Norms Are Associated with Infant and Young Child Feeding Practices in Bangladesh. *The Journal of Nutrition*. 2019;149:2034–45.

47. Sloand E, Gebrian B. Fathers clubs to improve child health in rural Haiti. *Public Health Nurs*. 2006;23:46–51.

48. Mukuria AG, Martin SL, Egondi T, *et al.* Role of Social Support in Improving Infant Feeding Practices in Western Kenya: A Quasi-Experimental Study. *Glob Health Sci Pract*. 2016;4:55–72.

49. Winter JC, Darmstadt GL, Lee SJ, *et al.* The potential of school-based WASH programming to support children as agents of change in rural Zambian households. *BMC Public Health*. 2021;21:1–13.

50. Brahma J, Pavarala V, Belavadi V. Driving Social Change Through Forum Theatre: A Study of Jana Sanskriti in West Bengal, India. *Asia Pacific Media Educator*. 2019;29:164–77.

51. Manaseki-Holland S, Manjang B, Hemming K, *et al.* Effects on childhood infections of promoting safe and hygienic complementary-food handling practices through a community-based programme: A cluster randomised controlled trial in a rural area of The Gambia. *PLoS Medicine*. 2021;18:1–24.

For peer review only

For peer review only - http://bmjopen.bmj.com/site/about/guidelines.xhtml

52. Kim SS, Nguyen PH, Tran LM, *et al.* Large-Scale Social and Behavior Change Communication Interventions Have Sustained Impacts on Infant and Young Child Feeding Knowledge and Practices: Results of a 2-Year Follow-Up Study in Bangladesh. *The Journal of Nutrition*. 2018;148:1605–14.

53. Akter F, Rahman M, Pitchik HO, *et al.* Adaptation and Integration of Psychosocial Stimulation, Maternal Mental Health and Nutritional Interventions for Pregnant and Lactating Women in Rural Bangladesh. *Int J Environ Res Public Health*. 2020;17:6233.

54. Chea Nguon, Lek Dysoley, Chan Davoeung, *et al.* Art and theatre for health in rural Cambodia. *Global Bioethics*. 2018;29. doi:10.1080/11287462.2017.1411762

55. Flax VL, Negerie M, Ibrahim AU, *et al.* Integrating group counseling, cell phone messaging, and participant-generated songs and dramas into a microcredit program increases Nigerian women's adherence to international breastfeeding recommendations. *Journal of Nutrition*. 2014;144:1120–4.

56. Ghosh SK, Patil RR, Tiwari S, *et al.* A community-based health education programme for bio-environmental control of malaria through folk theatre (Kalajatha) in rural India. *Malar J*. 2006;5:123.

57. Sanghvi T, Jimerson A, Hajeebhoy N, *et al.* Tailoring Communication Strategies to Improve Infant and Young Child Feeding Practices in Different Country Settings. *Food Nutr Bull*. 2013;34:S169–80.

58. Callery JJ, Sanann N, Tripura R, *et al.* Engaging ethnic minority communities through performance and arts: health education in Cambodian forest villages. *Int Health*. 2020;13:188–95.

59. Goodman G, Dent VF. A Story Grows in Rural Uganda: Studying the Effectiveness of the Storytelling/Story-Acting (STSA) Play Intervention on Ugandan Preschoolers' School Readiness Skills. *Journal of Infant, Child & Adolescent Psychotherapy*. 2019;18:288–306.

60. Sanghvi T, Haque R, Roy S, *et al.* Achieving behaviour change at scale: Alive & Thrive's infant and young child feeding programme in Bangladesh. *Maternal & Child Nutrition*. 2016;12:141–54.

For peer review only - http://bmjopen.bmj.com/site/about/guidelines.xhtml

61. Kuppers P. Community arts practices: Improvising being-together. *The Community Performance Reader*. Routledge 2007.

62. Kay A. Art and community development: the role the arts have in regenerating communities. *Community Development Journal*. 2000;35:414–24. doi:10.1093/cdj/35.4.414

63. Lowe S. Creating Community: Art for Community Development. *Journal of Contemporary Ethnography*. 2000;29:357–86. doi:10.1177/089124100129023945

64. Kwan BM, Brownson RC, Glasgow RE, *et al.* Designing for Dissemination and Sustainability to Promote Equitable Impacts on Health. *Annual Review of Public Health*. 2022;43:331–53. doi:10.1146/annurev-publhealth-052220-112457

65. Coombe CM, Chandanabhumma PP, Bhardwaj P, *et al.* A Participatory, Mixed Methods Approach to Define and Measure Partnership Synergy in Long-standing Equity-focused CBPR Partnerships. *Am J Community Psychol*. 2020; doi:10.1002/ajcp.12447.

66. Jones J, Barry MM. Exploring the relationship between synergy and partnership functioning factors in health promotion partnerships. *Health Promotion International*. 2011;26:408–20. doi:10.1093/heapro/dar002

67. de Peralta AM, Smithwick J, Torres ME, *et al.* Perceptions and determinants of partnership trust in the context of Community-Based Participatory Research. *Participatory Research*. 2020;13.

68. Charles J. How Partnership Trust can Facilitate and Result from CBPR: An Assessment of Situational, Organizational, and Institutional Related Factors. *EIJ*. 2021;5. doi:10.23880/EIJ-16000186

69. Cislaghi B, Heise L. Using social norms theory for health promotion in low-income countries. *Health Promotion International*. 2019;34:616–23. doi:10.1093/heapro/day017

70. Gautam OP, Curtis V. Food Hygiene Practices of Rural Women and Microbial Risk for Children: Formative Research in Nepal. *Am J Trop Med Hyg*. 2021;105:1383–95.

For peer review only - http://bmjopen.bmj.com/site/about/guidelines.xhtml

For peer review only

For peer review only - http://bmjopen.bmj.com/site/about/guidelines.xhtml

"Co-produced, arts interventions for nurturing care (0-5 years) in Low- and Middle-Income Countries (LMICs): a realist review."

Gale et. al (2024) BMJ OPEN

Supplementary materials

Supplementary Table 1: Search Strategy and Terms

Database	Outcomes	Arts	Community	LMIC
ProQuest – ASSIA and Education databases	In all fields:<br><br>('Child health' OR 'infant health' OR 'baby health' OR 'early years health' OR 'nurturing care' OR 'child care' OR 'child feeding' OR 'breastfeeding' OR 'hygiene' OR 'diarrhoea' OR 'sanitation' OR 'maternal health' OR 'maternal care' OR 'paternal health' OR 'paternal care' OR 'child development' OR 'child education' OR 'child wellbeing' OR 'maternal wellbeing' OR 'maternal mental health' OR 'child mental health' OR 'social cohesion' OR 'community cohesion' OR 'trust' OR 'community support' OR 'community development' OR 'social norms' OR 'health practices' OR 'care practices') AND	In abstract:<br><br>('Arts' OR 'drama' OR 'theatre' OR 'theater' or 'performative' OR 'creative' OR ' music' OR 'story' AND NOT 'antiretroviral') AND	In abstract:<br><br>('Community engagement' OR 'community mobilisation' OR 'community mobilization' OR 'community intervention' OR 'community-led' OR 'community campaign' OR 'group mobilisation' OR 'group mobilization' or 'participatory' or 'co-production' OR 'coproduction' OR 'coproduce' OR co-produce' OR 'mobilisation' OR 'mobilization') AND	In all fields:<br><br>('Low-income country' OR 'middle-income country' OR 'low income country' OR 'middle income country' OR 'LMIC' OR 'developing country' OR 'global south' OR Afghanistan OR 'Albania' OR 'Algeria' OR 'Angola' OR 'Antigua'<br>....<br>OR 'Yemen' OR 'Zambia' OR 'Zimbabwe'<br>)

For peer review only - http://bmjopen.bmj.com/site/about/guidelines.xhtml

SCOPUS	As above	As above	As above	As above but with countries selected in the filter by region or country option.
OVID: HMIC and Medline	As above	As above	As above	As above, but used OVID filter for country.
Web of Science	As above	As above	As above	As above, but Web of Science restricts the amount of keywords you can add to search query so filtered by country or region.
Child Education & Adolescence Studies	As above	As above	As above	As above, but for country used EBSCO filter.

For peer review only

Supplementary Table 2: Relevant Criteria for Inclusion

Include	Exclude
Study type:<br>- Performs and reports an intervention, whether in an randomized control trial (RCT) design or in other forms (Quantitative or qualitative)<br>- Theoretical or critical articulation of community-based arts-based interventions	Not opinions, letters or editorials
Intervention is co-produced (can be led by community or public health, or equally balanced)	Conducted over 20 years ago
Focuses on early years water/food sanitation (0-5) or child development	Any language other than English, French, Spanish
Intervention takes place in LMIC	***Added after discussion:***<br>Health or nurturing care not a primary outcome, but as an unintended (if positive) outcome
Intervention is arts-based	Maternal health only as outcome
Added after discussion:<br>Intervention is arts-based, toward public health and normative behaviour change	'Community health' does not contain measure(s) using specific to under 5s
	Theory only (though useful for background section)
	Arts/story-based methodological evaluation only
	Arts-based intervention is individual therapy (no participatory aspect)

For peer review only - http://bmjopen.bmj.com/site/about/guidelines.xhtml

Supplementary Table 3: Prompt questions used for appraisal of the interventions during data synthesis

appraise **co-production** by:	appraise **contextual adaptation** by:	appraise **arts-based implementation** by:
Whose input was sought/heard at which stage?	How was the context attended to by different phases or forms of contextual learning (e.g. observations, focus groups)?	What type of dramatic arts were utilised and who delivered/facilitated their use?
Was there balanced and reciprocal participation of stakeholders, researchers, local agents and community members in design/ implementation decisions?	Was there feedback (formal or informal) between design and implementation phases?	Was the intervention flexible? in the room? Was it adaptable logistically? i.e. time and place of delivery (to improve attendance)
Were any tensions or differences in goals noted between partners? If so, were causes and/or resolutions explored?	Was there explicit incorporation of local knowledge, local infrastructure or networks, local history and social/ economic/ geographic/ political differences or inequalities?<br>Note: beyond assuming this would happen automatically through participation of local people	Were any community groups excluded from participation? Were any groups notably less engaged, untrusting or sceptical upon delivery? Did any drop-out?
Intermediary-outcome hypotheses/ mid-range theories:<br>Is there evidence these brought about partnership synergy, partnership trust, targeting of equitably representative health/care/development issues, in a manner that are broadly supported and seen as effective. And, if so, to what extent?	*Intermediary-outcome hypotheses/ mid-range theories:*<br>Is there evidence these brought about effective, targeted health/care/ development information, and sustainable transmission of such information, equitable targeting of health/care issues. And, if so, to what extent?	*Intermediary-outcome hypotheses/ mid-range theories:*<br>Is there evidence these brought about explicit or experiential knowledge of health information, shared/communal experience of health information delivery, engaging with or challenging social norms, community alignment/ social binding or synergy, affective impact of health information delivery (e.g. motivation, shame, or empowerment). And, if so, to what extent?

For peer review only - http://bmjopen.bmj.com/site/about/guidelines.xhtml

Supplementary Table 4: Search results breakdown

DB search engine	Database(s)	Results
EBSCO	Anthropology Plus, ASSIA, CINAHL Plus, EconLit, Education Abstracts (H.W. Wilson), Ergonomics Abstracts, ERIC, Humanities Abstracts (H.W. Wilson), Humanities Full Text (H.W. Wilson)	516
OVID	APA PsycArticles, Embase, HMIC, CAB Abstracts and Global Health, Ovid MEDLINE(R) and In-Process, APA PsycInfo, Social Policy and Practice, BooksOvid, journalsOVID	211
Proquest	Arts & Humanities Database, Biological Science Database,East & South Asia Database, East Europe, Central Europe Database, Education Collection, Environmental Science Database, Health & Medical Collection, India Database, International Bibliography of the Social Sciences (IBSS), Middle East & Africa Database, Nursing & Allied Health Database, Political Science Database, Psychology Database, Public Health Database, Religion Database, Social Science Database, Sociology Collection	568
Scopus		266
Web of Science		42
Child Adolescence and Education Studies		38
	Total	1641
	Duplicates removed	940
	Titles/abstracts screening	175

For peer review only

Supplementary Figure 1: Search strategy process overview

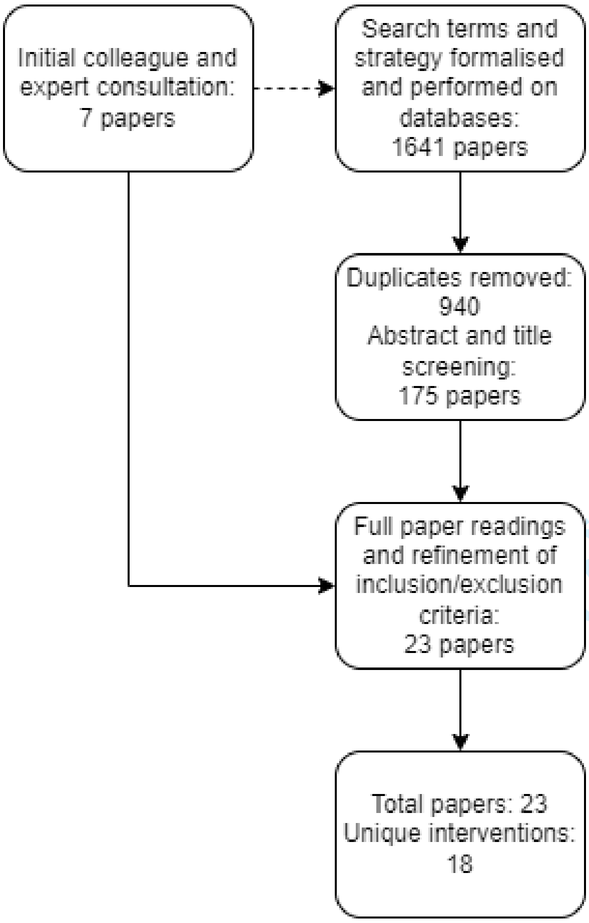

For peer review only - http://bmjopen.bmj.com/site/about/guidelines.xhtml

Supplementary Table 5: Summary of included papers

Author (Date)	Inter-vention Date	Title (s)	Country of intervention	Country of first author	Primary Discipline	Evaluation methods used	Intended Nurturing Care/Health Outcomes	Main Findings
Akter et al. (2020)	2017	Adaptation and Integration of Psychosocial Stimulation, Maternal Mental Health and Nutritional Interventions for Pregnant and Lactating Women in Rural Bangladesh	Bangladesh	Bangladesh	Public Health (Infectious Disease)	Qualitative evaluation (interviews) of a multi-phase adaptation process for an intervention.	Early childhood development	Most participants reported willingness to attend the sessions if extended for 1 year, and recommended additional visual cues and interactive role-play activities to make the sessions more engaging. Participants and community health workers found it difficult to understand the concept of "unhealthy thoughts" in the curriculum. This component was then revised to include a simplified behavior-focused story. Community health workers reported difficulty balancing the required content of the integrated curriculum but were able to manage after the contents were reduced. The revised intervention is likely feasible to deliver to a group of pregnant and lactating mothers in a low-resource setting.
Al-Delaimy et al. (2014)	2012-2013	Developing and evaluating health education learning package (HELP) to control soil-transmitted helminth infections among Orang Asli children in Malaysia	Malaysia	Iraq	Public health (Health Education & Community Medicine)	Mixed-methods approach: literature review, community discussions & impact evaluation of an open-label controlled intervention trial	Knowledge about soil-transmitted helminth infections	The developed package consists of a half day workshop for teachers, a teacher's guide book to STH infections, posters, a comic book, a music video, a puppet show, drawing activities and an aid kit. The package was well-received with effective contributions being made by teachers, children and their parents. The incidence rates of hookworm infection at different assessment points were significantly lower among children in the intervention school compared to those in the control school. Similarly, the intensity of trichuriasis, ascariasis and hookworm infections were found to be significantly lower among children in the HELP group compared to those in the control group ($P < 0.05$). Moreover, the package significantly improved the knowledge, attitude and practices (KAP) of Orang Asli people and the knowledge of teachers towards STH infections.

For peer review only - http://bmjopen.bmj.com/site/about/guidelines.xhtml

Callery et al. (2020)	2018	Engaging ethnic minority communities through performance and arts: health education in Cambodian forest villages	Cambodia	Thailand	Tropical Medicine	Qualitative evaluation (interviews)	Health information retention; assessing contextual-appropriateness	Respondents were able to recall the key health messages about malaria, antenatal care and infant vaccination. They also showed good awareness of malaria transmission and prevention and described how they enjoyed the events and appreciated the efforts of the project team. In isolated communities in Cambodia, a health education programme harnessing performance and arts engaged the whole community and its messages were readily recalled and prompted reflection.
Flax et al. (2014)	2011-2012	Integrating Group Counseling, Cell Phone Messaging, and Participant-Generated Songs and Dramas into a Microcredit Program Increases Nigerian Women's Adherence to International Breastfeeding Recommendations	Nigeria	US	Public Health Nutrition	Cluster randomised control trial evaluation of an intervention, interviews, and survey	Early breastfeeding initiation; exclusive breastfeeding	Among the clients who completed the final survey (n = 390), the odds of exclusive breastfeeding to 6 mo (OR: 2.4; 95% CI: 1.4, 4.0) and timely breastfeeding initiation (OR: 2.6; 95% CI: 1.6, 4.1) were increased in the intervention vs. control arm. Delayed introduction of water explained most of the increase in exclusive breastfeeding among clients receiving the intervention. In conclusion, a breastfeeding promotion intervention integrated into microcredit increased the likelihood that women adopted recommended breastfeeding practices. This intervention could be scaled up in Nigeria, where local organizations provide microcredit to >500,000 clients. Furthermore, the intervention could be adopted more widely given that >150 million women, many of childbearing age, are involved in microfinance globally.
Goodman and Dent (2019)	2014	A Story Grows in Rural Uganda: Studying the Effectiveness of the Storytelling/Story-Acting (STSA) Play Intervention on Ugandan Preschoolers' School Readiness Skills	Uganda	US	Psychotherapy	Randomised control trial and qualitative evaluation (caregiver interviews)	School readiness skills	Overall, participants benefited significantly from a story-reading activity with or without STSA. When examining both groups together (N = 121 post-intervention), school readiness skills significantly improved. Caregiver variables also predicted these three child outcome variables at baseline, suggesting that caregivers play a significant role in the development of their children's school readiness skills.

For peer review only

Gautam et al. (2017) Gautam and Curtis (2021)	2012-2013	Trial of a Novel Intervention to Improve Multiple Food Hygiene Behaviors in Nepal<br>Food Hygiene Practices of Rural Women and Microbial Risk for Children: Formative Research in Nepal	Nepal	UK	Tropical Medicine	Cluster randomised control trial evaluation of an intervention; Process evaluation and mixed-method analysis of an intervention adaptation process	Food hygiene behaviors	The five targeted food hygiene behaviors were rare at baseline (composite performance of all five behaviors in intervention 1%[standard deviation (SD)= 2%] and in control groups 2%[SD= 2%]). Six weeks after the intervention, the target behaviors were more common in the intervention than in the control group (43%[SD= 14%] versus 2%[SD= 2%], P= 0.02) during follow-up. The intervention appeared to be equally effective in improving all five behaviors in all intervention clusters. This study shows that a theory-driven, systematic approach employing emotional motivators and modifying behavior settings was capable of substantially improving multiple food hygiene behaviors in Nepal.
Ghosh et al. (2006)	2001	A community-based health education programme for bio-environmental control of malaria through folk theatre (Kalajatha) in rural India	India	India	Epidemiology (Malaria research)	Impact assessment (interviews assessing knowledge and behaviour) comparing intervention with control villages	Control of malaria	The exposed respondents had significant increase in knowledge and change in attitude about malaria and its control strategies, especially on bio-environmental measures ($p < 0.001$). They could easily associate clean water with anopheline breeding and the role of larvivorous fish in malaria control. In 2002, the local community actively co-operated and participated in releasing larvivorous fish, which subsequently resulted in a noteworthy reduction of malaria cases. Immediate behavioural changes, especially maintenance of general sanitation and hygiene did not improve as much as expected.
Kim et al. (2018)	2010-2014	Large-Scale Social and Behavior Change Communication Interventions Have Sustained Impacts on Infant and Young Child Feeding Knowledge and Practices: Results of a 2-Year Follow-Up Study in Bangladesh	Bangladesh	US	Nutrition	Cluster randomized nonblinded impact evaluation and comparison between more and less intensive intervention packages, via cross-sectional household survey	Infant and young child feeding knowledge and practices	In intensive areas, exposure to IPC decreased slightly between endline and follow-up (88.9% to 77.2%); exposure to CM activities decreased significantly (29.3% to 3.6%); and MM exposure was mostly unchanged (28.1–69.1% across 7 TV spots). Exposure to interventions did not expand in nonintensive areas. Most IYCF indicators in intensive areas declined from endline to follow-up, but remained higher than at baseline. Large differential improvements of 12–17 percentage points in intensive, compared with nonintensive areas, between baseline and follow-up remained for early initiation of and exclusive breastfeeding, timely introduction of foods, and consumption of iron-rich foods. Differential impact in breastfeeding knowledge remained between baseline

For peer review only

								and follow-up; complementary feeding knowledge increased similarly in both groups.
Kumar et al. (2018)	2011-2015	What it takes: evidence from a nutrition- and gender-sensitive agriculture intervention in rural Zambia	Zambia	USA	Food policy	Cluster-randomised, controlled, non-blinded, impact evaluation (cross-sectional household survey and child stunting measurements)	Child undernutrition (via women's empowerment, IYCF knowledge and practices and child anthropometry)	The RAIN project had positive effects on women's empowerment, IYCF knowledge, child morbidity and weight-for-height z-scores, but had little impacts on IYCF practices, and no impact on stunting. Strengthening programme implementation and fostering higher participation rates could support greater impacts on child nutrition outcomes.
Limaye et al. (2018)	2017	Nuestras Historias-Designing a novel digital story intervention through participatory methods to improve maternal and child health in the Peruvian Amazon	Peru	USA	Pediatrics	sequential exploratory mixed-method study design (qualitative: process of digital story curriculum creation; quantitative: acceptability)	Maternal mortality rates	According to the PhotoVoice workshops, pregnancy-related problems included: lack of partner support, domestic violence, early pregnancies, difficulty attending prenatal appointments, and complications during pregnancy and delivery. Over 30 stories on these themes were recorded. Seven were selected based on clarity, thematic relevance, and narrative quality and were edited by a professional filmmaker. The acceptability survey showed that local participants found the digital stories novel (M = 89.4%, F = 83.3%), relatable (M = 89.4%, F = 93.2%), educational (M = 91.5%, F = 93.3%) and shareable (M = 100%, F = 100%). Over 90% of respondents rated the digital stories as "Excellent" or "Good", found the videos "Useful" and considered them "Relevant" to their communities.

For peer review only - http://bmjopen.bmj.com/site/about/guidelines.xhtml

Manjang, et al. (2018) Manaseki-Holland et al. (2021)	2014-2015	Promoting hygienic weaning food handling practices through a community-based programme: intervention implementation and baseline characteristics for a cluster randomised controlled trial in rural Gambia.<br>Effects on childhood infections of promoting safe and hygienic complementary-food handling practices through a community-based programme: A cluster randomised controlled trial in a rural area of The Gambia	The Gambia	The Gambia/ UK	Public Health/ Paediatrics	Cluster randomised control trial	Complementary-food contamination and disease rates	For the primary outcome, the rate was 662/4,351(incidence rate [IR] = 0.15) in control villages versus 2,861/4,378 (IR = 0.65) in intervention villages (adjusted incidence rate ratio [aIRR] = 4.44, 95% CI 3.62–5.44, p < 0.001), and at 32 months the aIRR was 1.17 (95% CI 1.07–1.29, p = 0.001). We found that low-cost and culturally embedded behaviour change interventions were acceptable to communities and led to short- and long-term improvements in complementary-food safety and hygiene practices, and reported diarrhoea and acute respiratory tract infections.
McMichael & Robinson (2016)	2008-2012	Drivers of sustained hygiene behaviour change: A case study from mid-western Nepal	Nepal	Australia	Geography	Qualitative case-study evaluation (interviews, group discussions, drawings and household observation)	Water and sanitation-related diseases	Findings indicate the intervention has supported development of new norms around hygiene behaviours. Key drivers of sustained hygiene behaviour were habit formation, emotional drivers (e.g. disgust, affiliation), and collective action and civic pride; key constraints included water scarcity and socio-economic disadvantage. Identifying and responding to the drivers and constraints of hygiene behaviour change in specific contexts is critical to sustained behaviour change and population health impact.

For peer review only - http://bmjopen.bmj.com/site/about/guidelines.xhtml

Nguon et al. (2018)	2016-17	Art and theatre for health in rural Cambodia	Cambodia	Cambodia	Epidemiology (Parasitology)	Description & Reflection on lessons learnt	Dissemination of key health messages and supplementing existing engagement activities conducted by local authorities	We learnt the following lessons from our experience in Cambodia: involving local people including children from the beginning of the project and throughout the process is important; messages should be kept simple; it is necessary to take into consideration practical issues such as location and timing of the activities; and that the project should offer something unique to communities.
Olaide (2010)	Not stated	Maternal mortality and theatre intervention: A case study of Oluyole	Nigeria	Nigeria	Performing Arts	Descriptive case study evaluation	Maternal mortality rates	The paper discusses the practice of theatre for development as an information and enter-educative medium to the people most of whom are western educationally disadvantaged.

For peer review only - http://bmjopen.bmj.com/site/about/guidelines.xhtml

Sanghvi et al. (2013) Sanghvi et al. (2016) Nguyen et al. (2019)	2010-2016	Tailoring communication strategies to improve infant and young child feeding practices in different country settings; Achieving behaviour change at scale: Alive & Thrive's infant and young child feeding programme in Bangladesh; Information Diffusion and Social Norms Are Associated with Infant and Young Child Feeding Practices in Bangladesh	Bangladesh Ethiopia & Vietnam	USA	International Development	Cross country comparison of intervention design, adaptation phases and outcomes; Process description of multi-method intervention design and scale-up phases and evaluation via preliminary comparisons of intensive vs non-intensive treatment groups; Household surveys - analysis of paths from intervention exposure to IYCF practices through networks, diffusion, and norms (multiple regression analysis)	Exclusive breastfeeding and complementary feeding practices	Key messages: Well-designed and well-implemented large-scale interventions that combine interpersonal counselling, community mobilization, advocacy, mass communication and strategic use of data have great potential to improve IYCF practices rapidly. Formative research and ongoing studies are essential to tailor strategies to the local context and to the perspectives of mothers, family members, influential community members and policymakers. Continued use of data to adjust programme elements is also central to the process. Scale-up can be facilitated through strategic selection of partners with existing community-based platforms and through mass media, where a high proportion of the target audience can be reached through communication channels such as broadcast media. Sustaining the impacts will involve commitments from government and capacity building. The next step for capacity building would involve understanding barriers and constraints and then coming up with appropriate strategies to address them. One of the limitations we experienced was rapid transition of staff in key positions of implementing agencies, in government leadership, donors and other stakeholders. There was a need for continued advocacy, orientation and teaching related to strategic programme design, behaviour change, effective implementation and use of data.

For peer review only

For peer review only - http://bmjopen.bmj.com/site/about/guidelines.xhtml

Sloand and Gebrian (2006)	1994-2005	Fathers Clubs to Improve Child Health in Rural Haiti	Haiti	US	Nursing	Literature Review	Child and family health	Public health practitioners continually face grave challenges when addressing the health care needs in less developed countries. One such case is Haiti, where crushing poverty, poor infrastructure, a failing economy, natural disasters, and chaotic sociopolitical conditions compound these challenges. Public health practitioners, including nurses, must seek creative, culturally-appropriate, low technologic approaches to improve the health of the children and families in the remote villages of Haiti. The institution and support of village fathers clubs is one approach that has been ongoing since 1994. Fathers meet together on a regular basis for health education, support, and community building activities. The curriculum is health-based and facilitated by nurses, with participation by young and old men alike. Participants and organizers believe that family and child health is improved as a result of the groups.
Thuita et al. (2015) Mukuria et al. (2016)	2010-12	Engaging fathers and grandmothers to improve maternal and child dietary practices: planning a community-based study in Western Kenya Role of Social Support in Improving Infant Feeding Practices in Western Kenya: A Quasi-Experimental Study	Kenya	Kenya/ USA	Public Health	Quasi-experimental, non-equivalent comparison group design with pre- and post-testing (surveys, formative assessment and process evaluation); Quasi-experimental, multi-method, multi-phase intervention description and evaluation (survey)	Maternal and child dietary practices; Complementary feeding practices	We surveyed 554 people at baseline (258 mothers, 165 grandmothers, and 131 fathers) and 509 participants at endline. The percentage of mothers who reported receiving 5 or more social support actions (of a possible 12) ranged from 58% to 66% at baseline in the 3 groups. By endline, the percentage had increased by 25.8 percentage points (P=.002) and 32.7 percentage points (P=.001) more in the father and the grandmother intervention group, respectively, than in the comparison group. As the number of social support actions increased in the 3 groups, the likelihood of a mother reporting that she had fed her infant the minimum number of meals in the past 24 hours also increased between baseline and endline (odds ratio [OR], 1.14; confidence interval [CI], 1.00 to 1.30; P=.047). When taking into account the interaction effects of intervention area and increasing social support over time, we found a significant association in the grandmother intervention area on dietary diversity (OR, 1.19; CI, 1.01 to 1.40; P=.04). No significant effects were found on minimum acceptable diet.

For peer review only

Winter et al. (2021)	2017-2018	The potential of school-based WASH programming to support children as agents of change in rural Zambian households	Zambia	USA	Civil and Environmental Engineering	Quasi-experimental, prospective cohort intervention impact evaluation (open and closed surveys)	Sharing of WASH-related messages (washing hands and using the latrine) with caregivers	Student knowledge increased significantly, but primarily among students in grade 1. Overall rates of students reporting that they shared messages from the curriculum with their caregivers rose from 7 to 23% ($p < 0.001$). Students in grade 4 were 5.2 times as likely as those in grade 1 to report sharing a WASH-related message with their caregivers (ARR = 5.2, 95% C.I. = (2.3, 8.9); $p < 0.001$). Although we measured only modest levels of student dissemination of WASH UP! messages from the school to the home, students in grade 4 showed significantly more promise as agents of change than those in grade 1.

For peer review only

Supplementary Table 6: Summary of analysis and review findings

Author (Date)	Co-production	Adaptation to context	Use of dramatic arts	What works, for whom, & in what circumstances? Was it sustainable?	Scalability/ Transferability
Akter et al. (2020)	Researcher-led process, with feedback from participants, community members and community health workers.	Three existing interventions were integrated and culturally adapted via researcher knowledge and situation analysis (direct observation).<br><br>Two models of delivery were piloted - one in 'courtyard groups' and the other in individual home visits. Used local school infrastructure for delivery.	Arts-based approaches were not part of the original intervention but added after community feedback. Health messages were conveyed through use of visual cues, interactive role play and storytelling.<br><br>Delivered to communities by community health workers.	Participants reported willingness to attend the sessions if they were extended for 1 year. Changes recommended included additional visual cues and interactive role-play activities to make the sessions more engaging, a simplified behaviour-focused story (rather than the concept of 'unhealthy thoughts'), and to reduce the amount of content.<br><br>The study did not collect long-term data.	Small scale limits generalisability.<br><br>A systematic participatory approach was taken in intervention design, and a revised intervention was proposed which was likely feasible to deliver to a group of pregnant and lactating mothers in a low-resource setting.<br><br>Group sessions were as effective as combined ones and therefore potentially more cost-effective and scalable.
Al-Delaimy et al. (2014)	Researcher-led process, with engagement of a range of academic disciplines (e.g. public health, parasitology, education), local public health and community development officials, as well as residents, community members and service providers.<br><br>Limited opportunities for feedback during or after delivery.	Local adaptation of health education messages, via literature review and direct observation (household survey) by researchers of the Orang Asli communities, responding to a history of 'failed' interventions in the region.	Posters/comic books, nursery rhymes and songs (lyrics on posters and songs on YouTube and school computers) and a puppet show were used alongside more traditional learning materials.<br><br>Delivered to child-participants by teachers, who were trained in relevant health content.	Knowledge and behaviour improved (with variable onset, longevity and extent), and school infrastructure was inclusive of range of children.<br><br>No long-term outcomes, beyond 6 months (where control group had lower infections, but below statistical significance threshold). Long-term funding unclear.	The intervention used teachers making it scalable, but how willing or able teachers are to help will vary between individuals and places. The arts elements depended on availability of school materials e.g. computers, so may not be reproducible in all contexts.

For peer review only - http://bmjopen.bmj.com/site/about/guidelines.xhtml

Callery et al. (2020)	Researcher-led and stakeholder-funded. Involvement of a range of stakeholders at different levels, including Deputy District Governor, commune and village leaders, primary school principals, health centre chiefs, the police chief and village malaria workers.	A existing intervention was adapted to local context via a large stakeholder orientation meeting (n=~50) at a community health centre, where the activities, target villages and topics for community engagement were discussed.<br><br>This was followed by additional local, informal adaptation in each village, through meeting with commune and village leaders, the principal of the local school and village malaria workers to discuss with them the activities and inviting the students to participate.<br><br>TV was used to promote the events and celebrate.	Intervention was delivered by a community engagement team and members of a professional theatre group experienced in community/ village drama.<br><br>Participatory art and theatre workshops were delivered to school children to prepare for a village performance, which included wider members of the community. The workshops with schoolchildren were facilitated by teachers, with parental consent.<br><br>The performance included games, singing and dancing, health quizzes, a fashion show (showcasing traditional costumes, dance and musical instruments) and a comedy sketch about malaria. The closing event involved speeches, competitions, performances from professional singers, local traditional dances and an amateur singing competition.	Qualitative interviews suggested that knowledge of health messages had improved, with general recall of messages but not all details. Some participants shared the messages with non-participants. Involvement of children was noted to boost investment of families and teachers (and to require mastery of health messages for the performance).<br><br>Ethnic minority groups were specifically engaged and asked to be special guests at arts events in other villages. Showcasing elements helped the participants and the audience to gain a sense of ownership of the performances and this element was particularly popular among the local Kaviet and Lao populations.<br><br>No long term outcomes reported. Unclear whether further funding would be required or available.	This intervention was an adapted reproduction of preceding interventions, so can be considered reproducible. No immediate barriers to scaling were noted.

For peer review only

For peer review only - http://bmjopen.bmj.com/site/about/guidelines.xhtml

Flax et al. (2014)	Researchers partnered with a US NGO (Partners for Development) and 4 local, Nigerian community-based organizations.<br><br>Community members were recruited into microcredit groups of 5-7 friends, neighbours, or relatives who guaranteed each other's loans.	The intervention was delivered within an established social/economic grouping - women's micro-credit groups - that met monthly.<br><br>It was unclear whether there was any local adaptation or feedback process to the intervention.	Small groups of residents living near each other (in the microcredit groups) were asked to perform one of the key messages in a song or dramatic form.	Knowledge and behaviour improved, and intervention groups did breastfeed more at 3 and 6 months.<br><br>No longer term outcomes documented.	The microcredit aspect of this intervention may be difficult without American developmental organisation support. Could also cause social disruption instead of solidarity if poorly managed. Other aspects are low-cost and use local organisations.
Goodman and Dent (2019)	Researcher-led process, whereby researchers identified the problem and designed the intervention then, using translators, trained the local project coordinators to deliver the intervention.	The team ensured that the intervention was low cost and used local school infrastructure for delivery.	Storytelling and story-acting were used in the intervention. The story development was entirely child-led and 'spontaneous', integrated into play.	No difference overall between intervention and control group (who did drawing instead of story-telling). Some evidence that lower-income students and girls benefitted more.<br><br>No long-term outcomes reported. It was unclear whether further funding would be required or available.	Storytelling is low-resource and but limited evidence of effectiveness. The use of local schools for delivery would be scalable.

For peer review only - http://bmjopen.bmj.com/site/about/guidelines.xhtml

Gautam et al. (2017) Gautam and Curtis (2021)	Researcher-led process, drawing on literature and theoretical models of behaviour change (Behaviour-Centered Design), but also engaging a range of stakeholders and local communities. Government and non-governmental organizations' advised that the intervention adapted from preceding interventions in Mali and India would be replicable and scalable in Nepal. A local creative team was assembled to design the intervention. Local women with a similar profile to Nepal's ubiquitous Female Community Health Volunteers (FCHVs) an intervention package was designed and delivered, with a local creative team with expertise in marketing, design, innovation, program development, and behaviour change.	The intervention design was informed by formative research conducted by 4 local research field data collectors and five local women from different castes/ethnic groups using handheld cameras, and analysed by the research team. Following this group discussions with community members and health officials.<br><br>Several pilots took place for feedback from those delivering the intervention and the communities. Final feedback was from government officials and NGO stakeholders.	Local rallies, folk songs, games, rewards, storytelling, drama, cookery demonstrations and "kitchen makeover parties" were explicitly linked to the motivational drivers of behaviour identified in the guiding programme theory (Affiliation, Disgust, and Status).	The intervention changed five key behaviours observed in formative research to improve the microbiological environment. Women who engaged undertook public pledging and were celebrated with a public display of photos. The intervention was effective in differing socio-economic settings, although it is unclear if there were inequalities within settings in the women who did and did not participate.<br><br>No long term outcomes observed.	That the intervention was equally effective across targeted behaviours and across clusters is a positive indicator that the improvements are reproducible. Health workers found that it was feasible to deliver the intervention on top of their existing workload, suggesting potential scalability.

For peer review only

For peer review only - http://bmjopen.bmj.com/site/about/guidelines.xhtml

Ghosh et al. (2006)	Led by an intersectoral committee (involving ten governmental and non-governmental organisations), and delivered by local agents. Funded by the government, whose ministers provided visibility for the programme. Religious leaders contributed by offering free accommodation and hospitality for the period of one month as a token of solidarity in the fight against malaria. Local press and radio helped in wider dissemination of health education messages.<br><br>Local and regional organisations supported the intervention, e.g. the education department deputed five teachers and the Child and Women's Welfare Department deputed ten Anganwadi workers for one month.	Informal adaptation through a two-day visit from the intervention team prior to the final event.<br><br>Unclear if there were opportunities for feedback once the intervention had started.	Local educators, artists, religious leaders and community members collaborated to deliver the intervention.<br><br>Kalajatha is a popular, traditional art form of folk theatre depicting various life processes of a local socio-cultural setting . A local scriptwriter wrote 8 songs, two dramas and 4 rupakas (musical dramas) based on various aspects of malaria. These were then translated into skits using local dialects, musical styles and theatre traditions.<br><br>Thirty local artists (15 males and 15 females) from different occupational background were selected. Female artists were involved in the team, which resulted in better engagement from the women in the community.	Immediate behaviour changes were not evident but knowledge and attitudes of participants improved.<br><br>No long term outcomes were directly observed, but in a subsequent malaria prevention study via biocontrol in the area the year after this intervention, a dramatic improvement in malaria incidence was observed and in the study area people still refer to the Kalajatha events and the messages delivered to them, suggesting long term knowledge retention.	This intervention was implemented through and with existing local and regional organisations. The theatre performances themselves should not be highly expensive and require few materials.

For peer review only

Kim et al. (2018)	Donor-led initiative, working with researchers (from outside the country). The focus of the intervention shifted somewhat with new donors, who scaled up and adapted the programme. This is a top-down change (or feedback process).<br><br>The community-based infection and prevention control work was delivered by a international development organisation based in Bangladesh. Local health workers/volunteers/artists and regional/local infrastructures and organisations were used to deliver Infant and Young Child Feeding (IYCF) messages in regular meetings, community video and theatre shows, as well as radio and TV.	Broad consideration of context in intervention design (media, situation analysis, communications) concept testing, leading to creative development of prototype materials, (6) material pretests followed by revisions<br><br>The campaign implementation was subjected to ongoing monitoring, which was reported to lead to early and mid-course adjustments. However, evidence of bottom up changes/feedback incorporation in substantive content, or form of its delivery were not explicit.	Arts were only one component of a comprehensive programme of health promotion. Community video and theatre shows focused on infant and young child feeding. TV spots were broadcast nationally and regionally with intervention relevant messages.	This study offers evidence of decreased yet sustained impacts on IYCF practices even under modifications, suggesting program drift. Continued exposure infection prevention and control messages and sustained impacts on infant and young child feeding practices indicate lasting benefits from these interventions, as they underwent major scale-up and adaptations after termination of initial external donor support. At 2 y after endline, intervention exposure had decreased, but home visits by frontline workers remained moderately high in the intensive areas and significantly higher compared with the non intensive areas.<br><br>The programme is reliant on significant funding from external (non-governmental and internation) organisations, so sustainability is dependent on this continuing.	This project involved lots of government and non-government organisations. The practices of community arts-based health messaging is reproducible given low cost when working alongside existing organisations and with existing infrastructures.<br><br>In other contexts, where perhaps there was not be a broad political consensus on health priorities, expensive aspects of the current intervention may not be replicable, such as the TV ads. Reproducing this kind of comprehensive project at scale is challenging and demands significant collaboration.

For peer review only

Kumar et al. (2018)	International humanitarian organisation established partnerships with relevant government line ministries and with local implementing NGOs who were to deliver programme content across the different project components. Evaluation was conducted by International Food Policy Research Institute.	Used existing community health volunteers and women's groups, and existing government IYCF guidelines and materials to implement a gender-sensitive homestead food production programme with nutrition behaviour change communication.<br><br>Contextual issues were discussed in the paper and and initial gender analysis informed the intervention, but it is unclear whether there was any further adaptation informed by stakeholders.	Drama-based approaches were part of broader range of activities, including agricultural interventions (home gardening, providing seeds and equipment), and behaviour change components (group activities, radio messaging, posters, brochures) and women's empowerment (supply of fuel-saving stoves and fuel trees as labour-saving technologies). Community sensitisations were undertaken with assistance from drama groups, particularly around gender equality and its importance for improved nutrition. It is unclear whether these were participatory in delivery.	Positive effects were observed on women's empowerment, IYCF knowledge, child morbidity and weight-for-height z-scores, but little impact was seen on IYCF practices, and no impact on stunting. Areas that could be strengthened were programme implementation and fostering higher participation rates.<br><br>Long term outcomes were not observed.	The project was initially funded by international research organisations. It used local infrastructure and at times altered it, or offered incentives for participation (e.g. bicycles and/or agricultural inputs). The processes of adaptation to context (in design and delivery) are not very explicit.
Limaye et al. (2018)	Design of a digital story curriculum was participatory: the researcher ran three community-based PhotoVoice workshops with Community Health Workers and women who were invited by the CHWs; then they were involved in participatory digital storytelling activities. Stories were selected by the research team, who worked with a professional filmmaker and a professional midwife to develop these stories into a final digital story curriculum, where each story was combined with question-based teaching prompts.	The programme worked with existing community health workers, and the curriculum was iteratively developed with a variety of stakeholders.<br><br>There were limited opportunities later in the project for local storytellers to influence the final product (due to limitations of needing to process the digital stories in a city).	Photography (photo-voice) and community-based digital storytelling activities were used to inform the development of the curriculum. Participants were encouraged to think of personal stories and recorded themselves on mobile devices narrating their stories. Then participants did a storyboarding activity, in which they broke down the components of their story, and determined what types of photos would fit with each component of the story.	The digital story curriculum was highly acceptable to the target population, both men and women. They were considered novel, relatable, educational, and shareable.<br><br>Long term outcomes were not observed.	Solar-powered mobile phones with cameras are becoming cheaper and so the participatory process for designing a curriculum in this way is perhaps likely more replicable over time. Reproducibility was not investigated directly, but if able to utilise existing networks of local health workers and secure funding, there are no obvious issues.

For peer review only - http://bmjopen.bmj.com/site/about/guidelines.xhtml

Manjang, et al. (2018) Manaseki-Holland et al. (2021)	Local researcher-led, with wide consultation with health promotion agencies who were represented on a Local Scientific Advisory Committee in the Gambia (MOH, Unicef, WHO, University of the Gambia, National Nutrition Agency (NANA) and the MRC Gambia).<br><br>Locally, traditional communicators, community leaders and traditional birth attendants or other experienced women/ grandmothers were engaged. Women's achievements were celebrated individually (as MaaChampions) and communally (through villages achieving MaaChampion status).	The intervention was based on theoretical models of behaviour change and findings from previous similar intervention studies (in Nepal and India) and were adapted to the local context, based on formative research and consultation with stakeholders. The intervention material was translated into the three local languages (Mandinka, Wolof and Fula), field-tested with mothers and villagers and piloted iteratively by the intervention team.<br><br>TV was used to promote the events.<br><br>Women's networks were used to convey the messages 'onward' from the intervention, finding more volunteers. These women visited the families between the visits from the wider team to help establish the practices within the cultural norms of the community.	Performing arts (using culturally ingrained styles of drama and songs), animation, and stories.<br><br>Delivery team included traditional communicators (male and female, who use traditional African drumming, singing and acting), public health officers, a driver and a trained female volunteer, usually a traditional birth attendant (TBA).<br><br>Villagers reported fond memories of a 'joyous' programme with songs and activities of traditional performing artists, and they 'treasured' the achievements of MaaChampions in their family and community.	After intervention, behaviours improved (higher soap use in domestic kitchens and latrines) and there was a significant reduction in reported diarrhoea episodes and hospital admissions in the rainy season. No information was available about any inequalities in its effect.<br><br>Improved knowledge of the key behaviour messages was sustained at 6 months.<br><br>Qualitative data at 32 months suggested that mothers in intervention villages felt it their duty to inform mothers about complementary-food safety and hygiene practices when visiting other villages.	Cost-minimising tips were discussed toward replicability. Local structures and personnel used, with a low cost intervention.<br><br>This study builds on a smaller cluster RCTs of similar interventions in Nepal and India. This suggests the potential for reproducibility across different LMICs, and scope for scaling.<br><br>This low-cost intervention was not only acceptable to the mothers and the communities at large but was, at least in part, self-sustaining since new mothers reported practicing the behaviours at 32 months.

For peer review only - http://bmjopen.bmj.com/site/about/guidelines.xhtml

McMichael & Robinson (2016)	Researcher-led, in partnership with Australian and Nepal Red Cross. Two non-local researchers led the data collection with the assistance of a Nepali bilingual research assistant and two Nepali interpreters (one male, one female). Local residents were involved in some elements, e.g. children in participatory hygiene education and activities, group/committee formation (e.g. junior/youth Red Cross circles, women's groups, Water User Committees) and employment of local Village Health Motivators to conduct community-based hygiene education and home visits. Significant changes were made as a result of community feedback: for example, the original intervention employed Community-Led Total Sanitation triggering tools to create demand for latrines and improved hygiene (e.g. "walk of shame", flagging faeces) which were found to be 'difficult' and removed when all used the toilets. Positive motivational drivers were reported to be more effective.	Feedback was sought in focus group discussions and in depth-interviews. These were used to evaluate intervention impact. Ongoing program monitoring was noted but is unclear how this may have impacted adaptation beyond the informal adaptation through participatory activities.<br><br>Arts-based elements used as part of a broader intervention programme addressing different dimensions of the socio-economic and public health context: including provision of partially subsidised hardware; group/committee formation; hygiene and sanitation education; distribution of WASH information and education materials; employment of local Village Health Motivators to conduct community-based hygiene education and home visits; peer-led WASH awareness raising in schools/community; hygiene and sanitation campaigns; faeces calculation and fining of open defecation; and establishment of community funds that provide small loans for toilet construction.	Children and community members were engaged through the arts to raise awareness of WASH - through street drama and song.	Qualitative evidence showed changes in hygiene knowledge, behaviour and development of new social norms around hygiene. There was some anecdotal evidence of improvements in health.<br><br>Contextual limitations included the prohibitive cost of further latrine construction, and an inconsistent water supply which limited flushing and handwashing practice.<br><br>This paper presents qualitative reports on the interventions impact two years after beginning. The project's impact appears to have been supported by the diverse and intensive behaviour change activities that were repeated over a long period time by peers as well as community leaders.	Small-scale and intensive nature limits the applicability of these findings to large-scale WASH interventions.<br><br>Latrine construction could not persist due to the cost.

For peer review only - http://bmjopen.bmj.com/site/about/guidelines.xhtml

Nguon et al. (2018)	Led by local researchers, in partnership with the provincial health authorities, who selected and trained a local drama group to deliver the intervention based on a competitive process.<br><br>Community members were involved through attending the workshops, which led to the local tailoring of content for the public performances, and in quizzes/competitions.<br><br>High trust and synergy was reported between performers and the community.	This project (a pilot science-arts initiative) supplemented existing engagement activities conducted by local authorities and a community engagement team supporting a malaria elimination study (clinicaltrials.gov NCT01872702).<br><br>There was no formative research or formal adaptation process, but workshops were held in each village by the drama team, to adapt the performance using real stories of locals' experiences with malaria.	In each village there was a two and a half-day workshop led by a team consisting of local drama groups and health workers. There was a 'memorable' public performance on the third evening, incorporating local legends and real stories from the community. Community members, including children, were engaged using comedy, drama, drawing, singing competitions and quizzes about the intervention messages with prizes offered.	The drama storylines and health messages were remembered across the groups, and there was media interest in the project.<br><br>Attendance was lower proportionally in larger villages.<br><br>No long-term outcomes reported.	Intervention attracted large audiences in the villages, and offered something unique to remote, rural villages - especially involvement of professionals and the opportunity for children to be performing on stage alongside professional actors and seeing themselves in newspapers and on television. This is likely reproducible in other contexts, but not necessarily scalable as it would need to be tailored.

For peer review only

Olaide (2010)	Researchers, local drama groups, participants, stakeholders included government, health/medical and environmental establishment figures were involved in the design process.<br><br>The drama was intended to become the 'theatre of the people, by the people and for the people' (based on the Boalian concept of forum theatre) which aims not to find the 'best' solution but to produce a variety of options that could be used in the type of situation.	Communal research was conducted early in the process and identified that the most appropriate approach would be an 'inside-out approach' which would enable facilitators to interact within the community, gaining their trust and confidence and identify a 'joint panacea to the problems'.<br><br>The first step was the involvement of an existing and popular community-based theatre group in the dramatic endeavour data analysis, scenario development, improvisation rehearsal, performance and post-performance discussion. Also involved are some individuals within the community in the play presentation and the relevant themes were discussed after the performance in a live Q&A the programme took three days to put together.	Theatre performances, storytelling, later involved were drumming, singing and dancing which also made use of the songs that were known to them, most especially the nursing mothers and the pregnant women.<br><br>The performance comprised of 5 different but complementing sketches that were based on the causes and preventive measures of maternal mortality. The sketches were spiced up and linked together with different maternal songs that were folkloric, especially to the community members of the local government area.	The study appears to have caught the attention of and galvanised the community around issues of maternal mortality based on the qualitative reports cited in the paper.<br><br>The cost as with other local theatre groups should be low.<br><br>Long term outcomes were not observed.	No obvious barriers to scalability or reproduction were noted given low cost of theatre and success of other similar interventions.

For peer review only - http://bmjopen.bmj.com/site/about/guidelines.xhtml

Sanghvi et al. (2013)<br>Sanghvi et al. (2016)<br>Nguyen et al. (2019)	Partnerships with stakeholders were vital to expand the program's reach and to sustain program activities after initial funding ended. Partners were engaged in the research and design process to give implementing partners ownership of the strategy.<br><br>Government staff were trained by national and master trainers who were in turn trained by Alive and Thrive (A&T). The programme also launched a mass media campaign through inserts in newspapers (local journalists).	In each of the countries, Alive & Thrive convened stakeholders to review research to: (1) identify a few priority IYCF practices that would be the focus of the activities; (2) assess the degree to which select behaviours were feasible from family members' point of view and identified the conditions.<br><br>For all three countries, ProPAN methods were adapted and supplemented with additional tools, such as focus group discussions to gather information from frontline heath workers, fathers, grandmothers, and community leaders. The situational analysis in each country involved stakeholder consultations and reviews of existing data sets and reports to identify strategic choices and gaps in the data. Media audits, conducted by media research firms, guided two decisions: how much effort to devote to implementing or improving the Code of Marketing of Breast-Milk Substitutes, and what media channels to use for reaching different audience segments.	In all three countries, the mass media campaign containing emotionally appealing mini-dramas with various messages to spread the perception of recommended practices as normative was designed for a run of at least 2 years to build up audience recognition and recall. The campaign's television ads were loaded onto DVDs and shown on screens in outlying areas with low electricity, using portable generators, and the frontline worker training included a session using the television spots. Rural communities in media dark areas engaged through other mediums such as theatre.	Local adaptation, engagement of multiple stakeholders and scaling up were key elements of these projects. Combining face-to-face interventions for mothers with social mobilization and mass media was effective in improving IYCF practices.<br><br>The broad campaigns may both help and hinder sustainability. Helpful in that broad audiences through information diffusion can help transform norms quickly for many. A hinderance in the complexity of messaging with locally adapted schedules, content as well as their organisation. Partnerships with local organisations potential helped sustain the programmes after initial funding.	The campaign was delivered at a large scale, reaching an estimated 8.5 million. Scale-up was achieved through streamlining of tools and strategies, government branding, phased expansion through a local non-governmental implementing partner with an extensive community-based platform – and nationwide mainstreaming through multiple non-governmental organization and government programmes. The process has also been replicated in three countries. Without external (international) funding the scale of this study may not be replicable.

For peer review only - http://bmjopen.bmj.com/site/about/guidelines.xhtml

Sloand and Gebrian (2006)	Haitian Health Foundation (HHF), which is supported by both private donations and public funding, instituted fathers clubs in 1994. The fathers meet regularly, usually weekly or every 2 to 3 weeks, to learn about a wide range of child and family health topics and the related household- and family-level solutions. They discuss how to support their wives in the care of their children. They share family problems and help each other when needed. Meetings were described as 'very participatory'. The meetings are led by the fathers but attended by health agents (community health workers) and often a nurse who supervises the health agent. Together, they provide health education and support to the fathers.	Topics are decided by group discussion and fathers also engage in wider local infrastructure/project issues.	The focus of meetings is usually a child or family health issue, and discussion, songs, and skits are used to enhance learning. To reach out beyond the group, they help organise village health fairs with mothers' clubs or village health committees.	Fathers reported changes in knowledge, behaviour, social norms and skills, as well as health benefits (although these were not confirmed in observational studies). Father involvement is higher than expected based on other areas in Haiti.<br>Fathers report believing that the health and well-being of all the village children is the shared responsibility of all the adults in the village.<br>It is unclear if there are inequalities in access or participation for fathers.<br><br>The Fathers Club project has been running since 1994 in Haiti, so can be considered very sustainable in this context. However, long term outcomes were not formally evaluated/reported.	Low resources and low development context of this intervention imply high reproducibility across contexts. The use of existing health agents improves scalability also, however in contexts without a private/voluntary health foundation to provide support and organise reproducing and scaling may be more difficult.

For peer review only

Thuita et al. (2015); Mukuria et al. (2016)	International aid agency funded the project, which engaged volunteer community health workers (CHWs). These CHWs form community health committees and elect their leaders. In functional units, CHWs and committees are actively providing and overseeing community health surveys.<br><br>Each dialogue group selected one of its<br>members to serve as the group mentor, who was trained and assisted by a volunteer CHW. Feedback was provided as well. Before each meeting, mentors selected the discussion topic based on member interest and then facilitated discussions and activities with group members.<br><br>Tea allowance was provided to all groups during meetings, however, fathers preferred money (100 ksh/ 1 USD).	Formative research designed key messages for fathers and grandmothers in households in western Kenya. Peer dialogue groups were used to facilitate behaviour change among fathers and grandmothers by helping them gain new knowledge, share experiences and reflections, and apply communication and problem-solving skills that in turn would facilitate behaviour change in mothers.<br><br>Existing health facilities were used to host events. Trained CHWs conducted home visits with study participants in both the intervention and comparison groups and shared oral and written complementary feeding and maternal nutrition messages. In the intervention areas, fathers or grandmothers were invited to participate in this visit.	Dialogue-based groups were used to engage infants' fathers and grandmothers to support optimal infant feeding practices. Group members participated in role plays, problem solving activities, storytelling, and cooking demonstrations; grandmothers also composed songs promoting recommended practices. They showcased what they were learning through songs, skits, dances, and testimonials from fathers, mothers, grandmothers, CHWs and children, at Community Bazaars.	Family-centred approach, through engaging fathers and grandmothers of infants to improve their knowledge of optimal infant feeding practices and to encourage provision of social support to mothers could help improve some feeding practices. It is unclear whether all groups were engaged in the activities. Arts were used for community mobilization activities.<br><br>The use of local infrastructure is promising for sustainability of the intervention. The bazaars are low cost also.	Low cost and resource usage. Builds on existing infrastructure.

For peer review only - http://bmjopen.bmj.com/site/about/guidelines.xhtml

Winter et al. (2021)	World Vision and Sesame Workshop collaboratively developed the WASH UP! program. WASH UP! combines a 12-week educational curriculum created by Sesame Workshop targeting students in grades 1-4 with school WASH infrastructure improvements carried out by World Vision. Teacher training is conducted by Sesame Workshop over the course of 3 days, and materials distribution and infrastructure improvements are funded and implemented by World Vision. The curriculum includes 12 sessions, each of which is approximately 30-40 min long and uses child-friendly pedagogical strategies such as play-based learning.<br><br>The twelve sessions were designed to be conducted before, during, or after normal school hours, at the discretion of the teacher. A learning object was distributed to a random sub-set of five of the 12 schools. Teachers provided the learning object to students with the instruction to take it home and discuss a key message about handwashing from the WASH UP! curriculum with their caregivers.	helping families to establish and maintain handwashing stations close to the place of child feeding rather than focusing on the message 'wash your hands with soap'.<br><br>Mass media and community mobilization were used to create an enabling environment by changing perceptions about using soap as the norm. Communication materials and a TV spot focused on convenience - establishing a handwashing close to the place of food preparation. A training session on handwashing stations was also inserted in the national IYCF training manual.	Activities include listening to the teacher read from a story book, playing interactive games, structured discussion of key curriculum messages, and watching two 10-min videos.	No evidence of effectiveness of children as agents of change.<br><br>Disruption to teacher's schedules and workloads were noted, this also was an image due to potential reputational damage for World Vision within such communities where they work. This necessitated some adaptation of scheduling of intervention delivery. Projectors and videos were included as part of the curricular materials, but three of the twelve schools did not have electricity, making one of the twelve sessions much more challenging to administer	Low cost, low resource (is theatre less expensive than video? Likely more engaging, more easily made participatory and local)

For peer review only - http://bmjopen.bmj.com/site/about/guidelines.xhtml

RAMESES CHECKLIST

List of items to be included when reporting a realist synthesis		Page number
TITLE	1 In the title, identify the document as a realist synthesis or review	1
ABSTRACT	2 While acknowledging publication requirements and house style, abstracts should ideally contain brief details of: the study's background, review question or objectives; search strategy; methods of selection, appraisal, analysis and synthesis of sources; main results; and implications for practice.	1
INTRODUCTIO N	3 Rationale for review<br>Explain why the review is needed and what it is likely to contribute to existing understanding of the topic area.	4-5
	4 Objectives and focus of review<br>State the objective(s) of the review and/or the review question(s). Define and provide a rationale for the focus of the review.	5-6
METHODS	5 Changes in the review process<br>Any changes made to the review process that was initially planned should be briefly described and justified.	6
	6 Rationale for using realist synthesis<br>Explain why realist synthesis was considered the most appropriate method to use.	5
	7 Scoping the literature<br>Describe and justify the initial process of exploratory scoping of the literature.	6
	8 Searching processes<br>While considering specific requirements of the journal or other publication outlet, state and provide a rationale for how the iterative searching was done. Provide details on all the sources accessed for information in the review. Where searching in electronic databases has taken place, the details should include, for example, name of database, search terms, dates of coverage and date last searched. If individuals familiar with the relevant literature and/or topic area were contacted, indicate how they were identified and selected.	6
	9 Selection and appraisal of documents<br>Explain how judgements were made about including and excluding data from documents, and justify these.	7
	10 Data extraction<br>Describe and explain which data or information were extracted from the included documents and justify this selection.	7-8
	11 Analysis and synthesis processes<br>Describe the analysis and synthesis processes in detail. This section should include information on the constructs analyzed and describe the analytic process.	8
RESULTS	12 Document flow diagram<br>Provide details on the number of documents assessed for eligibility and included in the review with reasons for exclusion at each stage as well as an indication of their source of origin (for example, from searching databases, reference lists and so on). You may consider using the example templates (which are likely to need modification to suit the data) that are provided.	Supplementar y Figure 1
	13 Document characteristics<br>Provide information on the characteristics of the documents included in the review.	9-10
	14 Main findings<br>Present the key findings with a specific focus on theory building and testing.	10-16
DISCUSSION	15 Summary of findings<br>Summarize the main findings, taking into account the review's objective(s), research question(s), focus and intended audience(s).	16-17
	16 Strengths, limitations and future research directions<br>Discuss both the strengths of the review and its limitations. These should include (but need not be restricted to) (a) consideration of all the steps in the review process and (b) comment on the overall strength of evidence supporting the explanatory insights which emerged. The limitations identified may point to areas where further work is needed.	21
	17 Comparison with existing literature<br>Where applicable, compare and contrast the review's findings with the existing literature (for example, other reviews) on the same topic.	17-21
	18 Conclusion and recommendations<br>List the main implications of the findings and place these in the context of other relevant literature. If appropriate, offer recommendations for policy and practice.	21-23
	19 Funding<br>Provide details of funding source (if any) for the review, the role played by the funder (if any) and any conflicts of interests of the reviewers.	23

For peer review only
